# A Robust Analytical Pipeline for Genome-Wide Identification of the Genes Regulated by a Transcription Factor: Combinatorial Analysis Performed Using gSELEX-Seq and RNA-Seq

**DOI:** 10.1371/journal.pone.0159011

**Published:** 2016-07-13

**Authors:** Takaaki Kojima, Emi Kunitake, Kunio Ihara, Tetsuo Kobayashi, Hideo Nakano

**Affiliations:** 1 Department of Bioengineering Sciences, Graduate School of Bioagricultural Sciences, Nagoya University, Furo-cho, Chikusa-ku, Nagoya 464-8601, Japan; 2 Department of Biological Mechanisms and Functions, Graduate School of Bioagricultural Sciences, Nagoya University, Furo-cho, Chikusa-ku, Nagoya 464-8601, Japan; 3 Center for Gene Research, Nagoya University, Furo-cho, Chikusa-ku, Nagoya, 464-8602, Japan; Woosuk University, REPUBLIC OF KOREA

## Abstract

For identifying the genes that are regulated by a transcription factor (TF), we have established an analytical pipeline that combines genomic systematic evolution of ligands by exponential enrichment (gSELEX)-Seq and RNA-Seq. Here, SELEX was used to select DNA fragments from an *Aspergillus nidulans* genomic library that bound specifically to AmyR, a TF from *A*. *nidulans*. High-throughput sequencing data were obtained for the DNAs enriched through the selection, following which various *in silico* analyses were performed. Mapping reads to the genome revealed the binding motifs including the canonical AmyR-binding motif, CGGN_8_CGG, as well as the candidate promoters controlled by AmyR. In parallel, differentially expressed genes related to AmyR were identified by using RNA-Seq analysis with samples from *A*. *nidulans* WT and *amyR* deletant. By obtaining the intersecting set of genes detected using both gSELEX-Seq and RNA-Seq, the genes directly regulated by AmyR in *A*. *nidulans* can be identified with high reliability. This analytical pipeline is a robust platform for comprehensive genome-wide identification of the genes that are regulated by a target TF.

## Introduction

Transcription factors (TFs), which bind preferentially to certain DNA sequences, play the central role of transcriptional regulation in all organisms by interacting with *cis*-regulatory regions of DNA, such as promoters and enhancers [1]. Therefore, identifying the binding sites of a TF is crucial for analyzing the regulatory transcriptional networks of the TF. For identifying TF-binding sites, the method used most frequently is chromatin immunoprecipitation followed by sequencing (ChIP-Seq), which is performed after formaldehyde-mediated TF–DNA crosslinking. Therefore, this technique only provides a snapshot of TF binding that is obtained in a particular cell at the time of the formaldehyde crosslinking. Consequently, to identify most of the biologically relevant DNA-binding sites of a TF, the same analysis must be repeated under different cell-culture conditions [2].

Systematic evolution of ligands by exponential enrichment (SELEX) is an *in vitro* method for selecting the nucleic acids that can be bound specifically by a target of interest from an initially random sequence pool [3–5]. SELEX can also be used for the screening of the DNA-binding sequences of various TFs [5–8]. Recently, SELEX-Seq was developed as a high-throughput SELEX technique for characterizing the DNA-binding specificity of TFs by using high-throughput DNA sequencing [9–14]. The general procedure mainly consists of the following 3 steps: i) SELEX-based selection of dsDNAs bound by a target TF from a random dsDNA sequence pool; ii) high-throughput DNA sequencing of the selected dsDNAs; and iii) bioinformatics analyses of the obtained sequence data to identify the binding motifs [14].

Genomic SELEX (gSELEX)—SELEX performed using a library derived from genomic DNA—enables the isolation of reliable TF-binding sites and their direct mapping within the genome [15, 16]. For example, Reiss and Mobley determined the binding sites of PapX through SELEX-Seq performed using a uropathogenic *Escherichia coli* genomic library [17]. Moreover, a recent study showed that the “sequence environment,” which includes the DNA shape features around a consensus motif, can help guide TFs to their cognate binding sites [18]. This finding underscores the importance of screening a genomic library, and not a synthetic library, for the *in vitro* exploration of TF-binding sites.

In high-throughput studies of gene expression, high-throughput RNA-sequencing (RNA-Seq) technology is now gradually replacing microarrays; this is because RNA-Seq enables differentially expressed genes (DEGs) to be identified with a higher resolution than microarrays do [19]. To date, RNA-Seq has been used successfully to analyze the transcriptomes of various organisms ranging from yeast [20] to human [21]. The transcriptomes of *Aspergillus* spp. fungi have also been analyzed using RNA-Seq, and the relevant databases can be accessed online [22, 23]. However, when RNA-Seq is used for identifying the DEGs that are affected by a target TF, it is extremely challenging to determine whether the expression of the DEGs is directly or indirectly regulated by the TF. Conversely, SELEX-Seq provides information regarding the *in vitro* binding sequences of a target TF, but not all binding sequences identified in a genome might be related to the regulation of the TF in cells. Therefore, the genes regulated by a target TF should optimally be identified using both *in vivo* and *in vitro* analyses.

**Fig 1:**
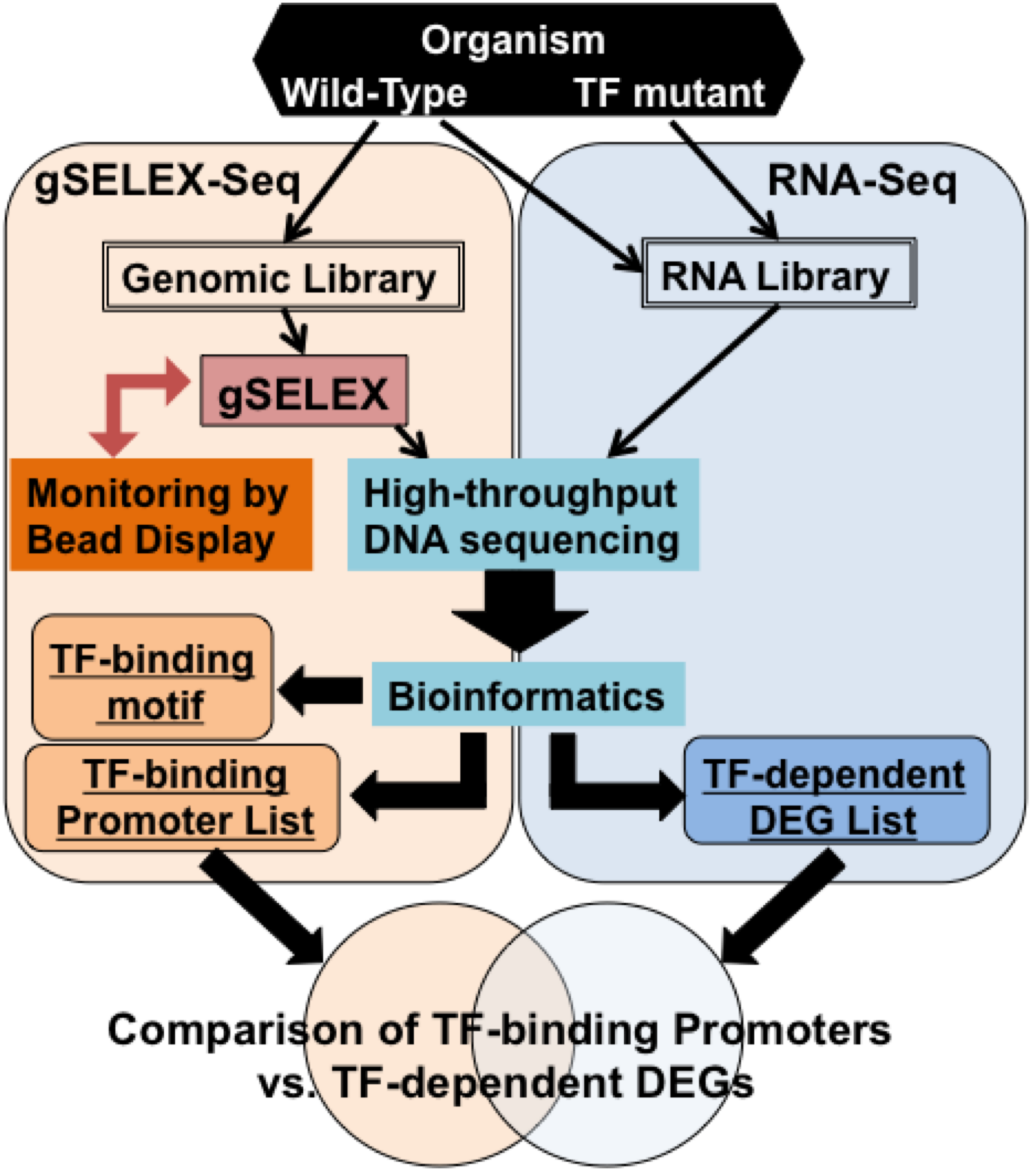
Flowchart of the combinatorial genome-wide analysis performed using gSELEX-Seq and RNA-Seq for identifying genes regulated by TFs.

Here, we report the establishment of a robust analytical pipeline combining gSELEX-Seq and RNA-Seq for the identification of several of the genes that are regulated by a TF (Fig 1). In this system, gSELEX is used for selecting the DNAs that a target TF specifically binds, following which high-throughput sequencing and bioinformatics analyses are performed. In parallel with the gSELEX-Seq procedure, RNA-Seq is used for identifying the DEGs modulated by the target TF. A comparison of the two profiles obtained enables genome-wide identification of the genes regulated by the TF.

AmyR is a fungal TF that activates transcription by binding to a CGGN_8_(C/A)GG sequence located within various amylase promoters [24–27]. We used our new analytical system to identify AmyR-regulated genes in the *A*. *nidulans* genome. In this analysis, the canonical binding motif, CGGN_8_CGG, was successfully read out from only a single round of the selection. Moreover, >2000 genes, including all 8 amylolytic genes that are currently known to be regulated by AmyR, were obtained as possible AmyR-dependent genes. However, AmyR is unlikely to regulate all of these genes in the *A*. *nidulans* genome, which suggests that, by itself, the information regarding the binding regions in the genome is insufficient for accurately understanding the AmyR regulation system. Next, we performed RNA-Seq in order to identify the DEGs modulated by AmyR following isomaltose induction in *A*. *nidulans*. The profiles obtained from the RNA-Seq analysis showed that 41 genes, including 7 of the 8 known AmyR-regulated amylolytic genes, were detected as AmyR-dependent and isomaltose-induction-dependent DEGs. The intersecting set of genes that were detected using both gSELEX-Seq and RNA-Seq contained 23 genes, including the 7 AmyR-regulated genes, which suggests that our analytical pipeline can serve as a robust platform for conducting transcriptome analysis.

## Materials and Methods

### Oligonucleotide primers

The sequences of the primers used in this study were the following: P1, 5'-CCACTACGCCTCCGCTTTCCTCTCTATG-3'; P2, 5'-CTGCCCCGGGTTCCTCATTCT-3'; P1-agdRIIp, 5'-CCACTACGCCTCCGCTTTCCTCTCTATGAAAGATCTGGTGGAGGCACTGCAAAATG-3'; P2-agdFBp, 5'-CTGCCCCGGGTTCCTCATTCTGGGGATCCTCGACTATAACAGCTTC-3'; P1-bio, 5'-Biotin-CCACTACGCCTCCGCTTTCCTCTCTATG-3'; and P2-Cy5, 5'-Cy5-CTGCCCCGGGTTCCTCATTCT-3'.

### Strains and growth conditions

Genomic DNA was prepared from *A*. *nidulans* ABPU1 cells (*biA1 pyrG89; wA3; argB2; pyroA4*) [25]. *A*. *nidulans* BPU7 (*biA1 pyrG89; wA3; pyroA4*) and *A*. *nidulans* Δ50 (*biA1 pyrG89; wA3; argB2; pyroA4; ΔamyR*::*argB*^*+*^) were used for mRNA extraction. The strain BPU7 was constructed by replacing the *argB2* allele of *A*. *nidulans* ABPU1 with *argB*^*+*^. The strain Δ50 is an *amyR* deletant described previously [25]. The strains were grown at 37°C in standard minimal medium (MM) supplemented with appropriate nutrients, as described previously [28]. *E*. *coli* JM109 and BL21 (DE3) were used for DNA manipulations and recombinant AmyR expression, respectively.

### Expression of recombinant AmyR in *E*. *coli*

A part of AmyR (residues 1–411; AmyR_1–411_) was expressed as a MalE (maltose-binding protein, MBP) fusion protein in *E*. *coli* by using the pMAL system as described by Kojima *et al*. [29], with a few modifications. The expression of MalE::AmyR_1–411_ was induced with isopropyl β-D-1-thiogalactopyranoside for 8 h at 16°C. After induction, the cells were harvested and washed with phosphate-buffered saline (PBS) (137 mM NaCl, 2.7 mM KCl, 10 mM Na_2_HPO_4_, 1.8 mM KH_2_PO_4_, pH 7.4), suspended in PBS containing 10 mM 2-mercaptoethanol (PBS/2-ME) and disrupted by sonicating on ice, and then centrifuged. The protein concentration of the obtained supernatant was determined as previously described [30], by using BSA as a standard. The concentration was adjusted to 200 μg/mL with PBS/2-ME for gSELEX (crude AmyR solution).

### Construction of an *A*. *nidulans* genomic library

Total DNA from *A*. *nidulans* ABPU1 was isolated using the cetyltrimethylammonium bromide method [31]. The extracted *A*. *nidulans* DNA was ultrasonically sheared to an average size of approximately 100 bp by using the DNA-shearing system M220 (Life Technologies, Carlsbad, CA, USA); the following shearing conditions were used: 20 cycles; bath temperature 5°C; duty cycle 10%, intensity 5; cycles/burst 100; time 60 s/cycle; and acoustic power 20 W. The sheared fragments were subsequently blunt-ended by using an End-It DNA End-Repair Kit (Epicentre, Madison, WI, USA) according to the manufacturer’s protocol. Linkers were prepared by annealing the corresponding primer-pairs: P1_Adaptor (5'-CCACTACGCC TCCGCTTTCC TCTCTATGGG CAGTCGGTGA T-3')/P1_Adaptor_comp (5'-ATCACCGACT GCCCATAGAG AGGAAAGCGG AGGCGTAGTG GTT-3') and P2_Adaptor_comp (5'-AGAGAATGAG GAACCCGGGG CAGTT-3')/P2_Adaptor (5'-CTGCCCCGGG TTCCTCATTC TCT-3'). Linker ligation was performed (for 2 h at 16°C) in a ligation mixture containing approximately 2 pmol of *A*. *nidulans* genomic fragments prepared using the method described above, 20 pmol each of the linker fragments, and the ligation mix (Takara Bio, Ostu, Japan) at twice the volume of the fragment mixture solution, in a total volume of 32.8 μL. After ethanol precipitation, the ligated products were size-fractionated on a 1.5% agarose gel. Bands of approximately 100–250 bp were excised using a spatula, and the DNA fragments were recovered by using a FastGene Gel/PCR Extraction Kit (Nippon Genetics, Tokyo, Japan) according to the manufacturer’s protocol. Next, 1 ng of this genomic library was amplified in a 20-μL PCR mixture containing 0.025 U/μL of *LA Taq* (Takara) and 0.25 μM each of the primers P1 and P2. The following temperature sequence was used: preheating at 94°C for 5 min, 12 cycles consisting of 94°C for 15 s, 62°C for 10 s, and 72°C for 4 s, followed by an additional extension at 72°C for 7 min. Amplicons were purified using a FastGene Gel/PCR Extraction Kit. The concentration of the library was assessed using a Quant-iT dsDNA Broad-Range Assay Kit (Invitrogen, Carlsbad, CA, USA), following the manufacturer’s instructions.

### gSELEX selection

The AmyR binding reaction was performed by mixing 20 ng of the *A*. *nidulans* genomic library with 100 μL of the 200 μg/mL crude AmyR solution and agitating the mixture for 30 min at room temperature. Next, 10 μL of amylose resin (New England BioLabs, Ipswich, MA, USA) was washed with 500 μL of MBP w/o EDTA buffer (200 mM NaCl, 20 mM Tris–HCl, 10 mM 2-mercaptoethanol, pH 7.5) and then the resin was suspended in a 1.5-mL tube in 900 μL of fresh MBP w/o EDTA buffer and mixed with the AmyR-binding reaction mixture. The suspension was mixed using a rotator for 1 h at 4°C, following which the resin was recovered by centrifuging the suspension at 300 × *g* for 1 min at 4°C. After removing as much of the supernatant as possible, the resin was suspended in 10 μL of MBP w/o EDTA elution buffer (200 mM NaCl, 20 mM Tris–HCl, 10 mM 2-mercaptoethanol, 20 mM maltose, pH 7.5) and the suspension was mixed using a rotator for 15 min at 4°C. Lastly, the supernatant was recovered after centrifugation at 300 × *g* for 1 min at 4°C.

The selected clones were amplified using a PCR reaction mixture (10 tubes × 20 μL) that included 0.025 U/μL *Ex Taq* DNA polymerase (Takara) and 0.25 μM primers (P1 and P2). The following program was used: preheating at 94°C for 30 s, followed by 14 cycles (in the first round) or 12 cycles (in the second round) of 94°C for 15 s, 62°C for 10 s, and 72°C for 3 s, and an additional extension at 72°C for 7 min. In the third round, the selected clones were amplified from 1 μL of the selected solution in 20 μL of the same PCR mixture by using the following PCR program: preheating at 94°C for 30 s; 10 cycles of 94°C for 15 s, 62°C for 10 s, and 72°C for 3 s; and a final extension at 72°C for 7 min. After purification (FastGene Gel/PCR Extraction Kit), the concentration of the library was assessed by using a Quant-iT dsDNA Broad-Range Assay Kit as per Invitrogen’s instructions.

### Analysis of relative AmyR-binding affinity by using bead display and flow cytometry

An agdAWT fragment containing the AmyR-binding sequence and an agdAΔ53 fragment containing a mutated AmyR-binding sequence were amplified from pBATWT and pBATΔ53, respectively [26], with the primer pairs P1-agdRIIp/P2-agdFBp, and then purified (FastGene Gel/PCR Extraction Kit).

The binding affinities of selected DNA pools were determined using bead display and flow cytometry [32]. The selected DNA fragments from gSELEX (from Rounds 0, 1, 2, and 3), agdAWT, and agdAΔ53 were PCR-amplified using the primers P1-bio and P2-Cy5, and the 6 amplicons were purified using a FastGene Gel/PCR Extraction Kit. The relative binding affinity of MalE::AmyR_1–411_ was examined as described by Wang *et al*. [32], with some modifications. We added approximately 150 ng of the biotin-labeled fragments separately onto 1.2 × 10^6^ M-280 streptavidin-coated beads (Dynabeads M-280 Streptavidin; Life Technologies, Carlsbad, CA, USA) and examined the relative AmyR-binding activity in each pool by performing flow cytometry (JSAN; Bay Bioscience, Kobe, Japan) and analyzing the data by using FlowJo software (Treestar, Ashland, OR, USA).

### DNA sequencing and data analysis in gSELEX-Seq

Each selected pool was used to generate Illumina paired-end sequencing libraries by using an NEBNext Ultra DNA Library Prep Kit for Illumina (New England BioLabs) and NEBNext Multiplex Oligos for Illumina (Index Primers Set1, New England BioLabs) according to the manufacturer’s instructions. The products were purified using the Agencourt AMPure XP system (Beckman Coulter, Brea, CA, USA), and the pools were sequenced using an Illumina HiSeq 2000 sequencer (BGI Japan, Kobe, Japan). All sequencing data will be made available under controlled access through the DNA Databank of Japan (DDBJ; accession number DRA004716).

The 5' and 3' adapters were stripped from the reads by using *Cutadapt* (v1.7.1) with the following parameters: -b CCACTACGCCTCCGCTTTCCTCTCTATGGGCAGTCGGTGAT -a ATCACCGACTGCCCATAGAGAGGAAAGCGGAGGCGTAGTGG -b CTGCCCCGGGTTCCTCATTCTCT -a AGAGAATGAGGAACCCGGGGCAG -O 15. The trimmed paired-end reads were mapped with *Bowtie* (v2) onto the *A*. *nidulans* genome [A_nidulans_FGSC_A4_current_chromosomes.fasta (http://www.aspgd.org)] with default settings. Peaks were called using *MACS* (v1.4.2) [33] with default settings except for the following options: -f BAM -g 32000000. Once the peaks were ranked based on fold-enrichment, the peak interval data were converted to the interval data of 50-bp sequences, which were cut out in each direction from the summit position by using *BEDTools* (v2.17.0) with the following parameters: bedtools slop -l 24 -r 24. The sequence data were extracted using the fastaFromBed utility in *BEDTools*. Motifs were identified by using *MEME* (v 4.10.2) with the following parameters: -dna -maxsize 500000 -nmotifs 5 -revcomp -maxw 20.

The possible promoters regulated by AmyR were annotated as follows: The *A*. *nidulans* upstream1000 dataset, which contains the 1000-bp region upstream of all of the predicted *A*. *nidulans* genes, was obtained using A_nidulans_FGSC_A4_current_orf_genomic_1000.fasta (http://www.aspgd.org). The 50-bp sequences obtained from the third round of selection were annotated using *A*. *nidulans* upstream1000 by local BLAST, by using the following parameters: blastn -evalue 10 -outfmt 6.

### Total RNA preparation

Total RNA was prepared from *A*. *nidulans* BPU7 and *A*. *nidulans* Δ50. The strains were grown in standard MM [28], containing 1% glycerol as the sole carbon source, at 37°C for 24 h. The mycelia were collected through filtration and washed in the same medium. Subsequently, 0.2 g (wet weight) of the mycelia were transferred to 20 mL of fresh MM containing 1% glycerol, with or without 0.1% isomaltose, the inducer of α-amylase production, and incubated at 37°C for 4 h. After induction, the mycelia were harvested, frozen in liquid nitrogen, and ground to a fine powder with an SK-mill (Tokken, Chiba, Japan). Total RNA was extracted using TRIzol Reagent (Thermo Fisher Scientific, Waltham, MA, USA), according to the manufacturer’s instructions, and then treated with a TURBO DNA-free Kit (Thermo Fisher Scientific) to remove DNA from the RNA preparations. RNA was isolated 3 separate times for each strain and condition, and a total of 12 RNA samples were then used for the next step. The concentration of total RNA was determined using a Qubit fluorometer and an RNA Assay Kit (Life Technologies). The integrity of the total RNA was determined by using an Agilent 2100 Bioanalyzer and performing an RNA Pico 6000 chip assay, in accordance with the manufacturer’s instructions (Agilent Technologies, Santa Clara, CA, USA). In all assayed samples, the RNA integrity number (RIN) was >8.0, which indicated that all samples were in good condition. From 10-μg total-RNA samples, poly(A) RNA was enriched using an mRNA Purification Kit (Magnosphere UltraPure, Takara), according to the manufacturer’s protocol.

### Library construction, MiSeq sequencing, and data analysis in RNA-Seq

From the obtained mRNAs, cDNA libraries were constructed using an NEBNext Ultra Directional RNA Library Prep Kit for Illumina. The 12 samples were discriminated using multiplex oligonucleotide DNAs (New England BioLabs). The final constructed libraries were quantified using the Qubit fluorometer and the average fragment sizes were determined by analyzing 1 μL of the libraries on the Agilent Bioanalyzer 2100 by using a High-Sensitivity DNA LabChip. Each library was mixed in equal amounts to contain a total of 4 nM cDNA. To denature the DNA, NaOH solution was added at a volume equal to that of the DNA libraries, following which 100-fold dilutions were performed using HT1 buffer (Illumina) to obtain the DNA libraries at 20 pM. The DNA libraries were further diluted with HT1 buffer to 15 pM at a total volume of 1 mL, and then loaded into the cartridge for MiSeq and sequenced as multiplex two-read libraries for 168 cycles (including 8 additional cycles for each of the index reads) according to the manufacturer’s protocol (Illumina). All sequencing data will be made available under controlled access through the DNA Databank of Japan (DDBJ; accession number DRA004717).

The DNA sequences obtained were mapped onto the reference genomic sequence of *A*. *nidulans* open reading frames (ORFs) [A_nidulans_FGSC_A4_current_orf_coding.fasta (http://www.aspgd.org)] by using the default settings. Further analysis and normalization were performed using *SeqMonk* (http://www.bioinformatics.bbsrc.ac.uk/projects/seqmonk/). DEG lists were generated using a statistical significance test (*P* < 0.05) and *DESeq2* and *EdgeR* software.

## Results and Discussion

*In vitro* characterization of the DNA-binding sites of TFs neither requires a culturing step nor depends on particular cell types or growth conditions. Therefore, *in vitro* technologies provide highly valuable support for the *in vivo* identification of TF-binding sites and are suitable for high-throughput analysis. SELEX-Seq is a high-throughput method that is suited for systematically characterizing the DNA-binding specificities of TFs. In a standard SELEX-Seq strategy, DNA targets are selected using an electrophoresis mobility shift assay (EMSA) [9, 14, 34] or affinity immobilization performed with streptavidin/streptavidin-binding peptide [10, 11] or MBP/amylose resin [9]. In this study, we used MBP/amylose resin to isolate the protein-bound DNA because MBP, which is frequently used as a fusion tag to improve protein solubility, enables soluble expression of AmyR_1-411_ in *E*. *coli* [26, 29].

**Fig 2:**
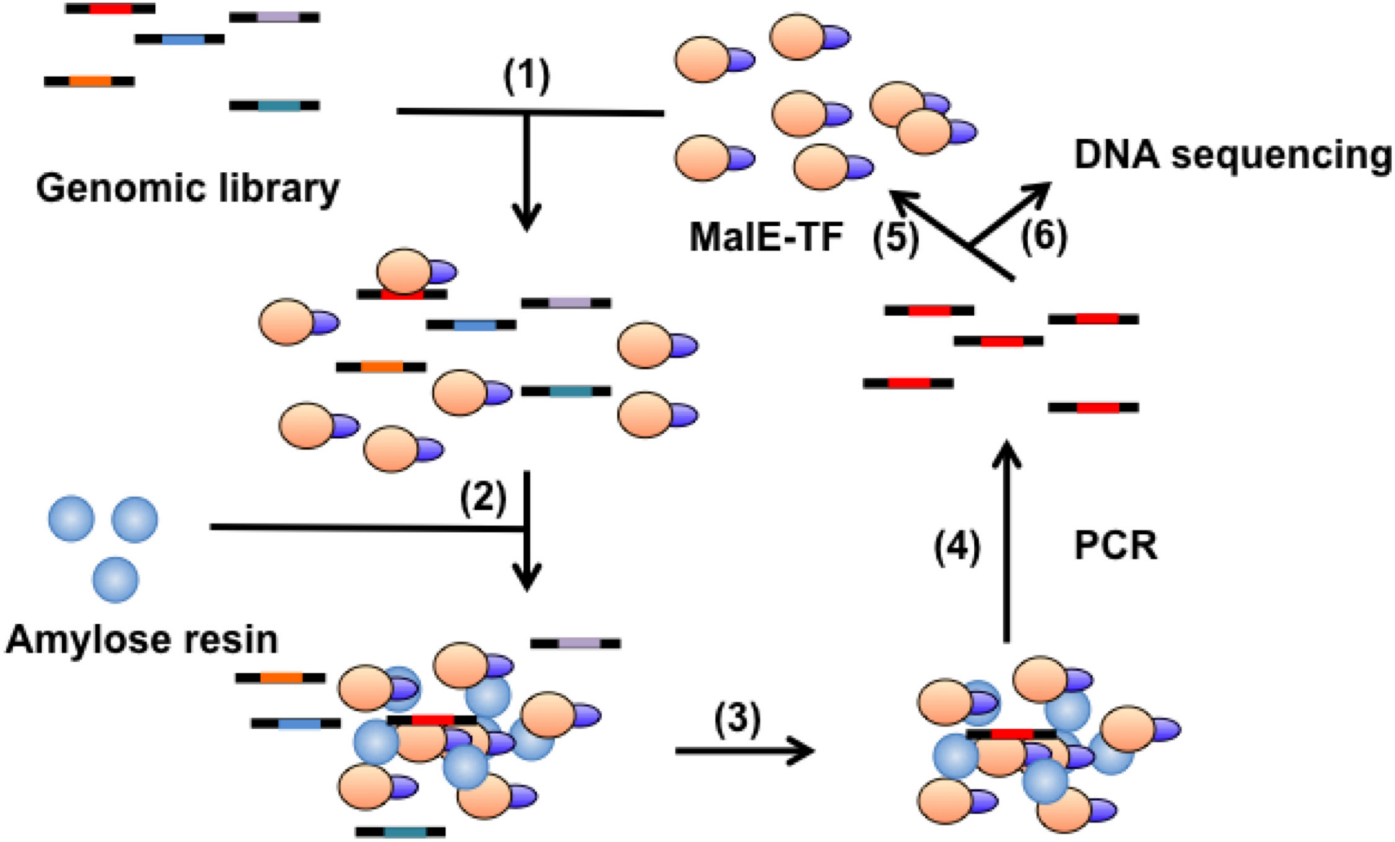
Schematic presentation of gSELEX-Seq used for selecting TF-binding sites in a genome. (1) A MalE-tagged TF is added to a genomic library mixture and the DNA-binding reaction of the TF is performed. (2) Amylose resin is added. (3) The MalE-tagged TF-amylose-resin complex is recovered. (4) The DNA fragments bound by the TF are amplified using PCR. This recovered DNA pool is used in a subsequent selection round (5) or high-throughput DNA sequencing (6).

**Fig 3:**
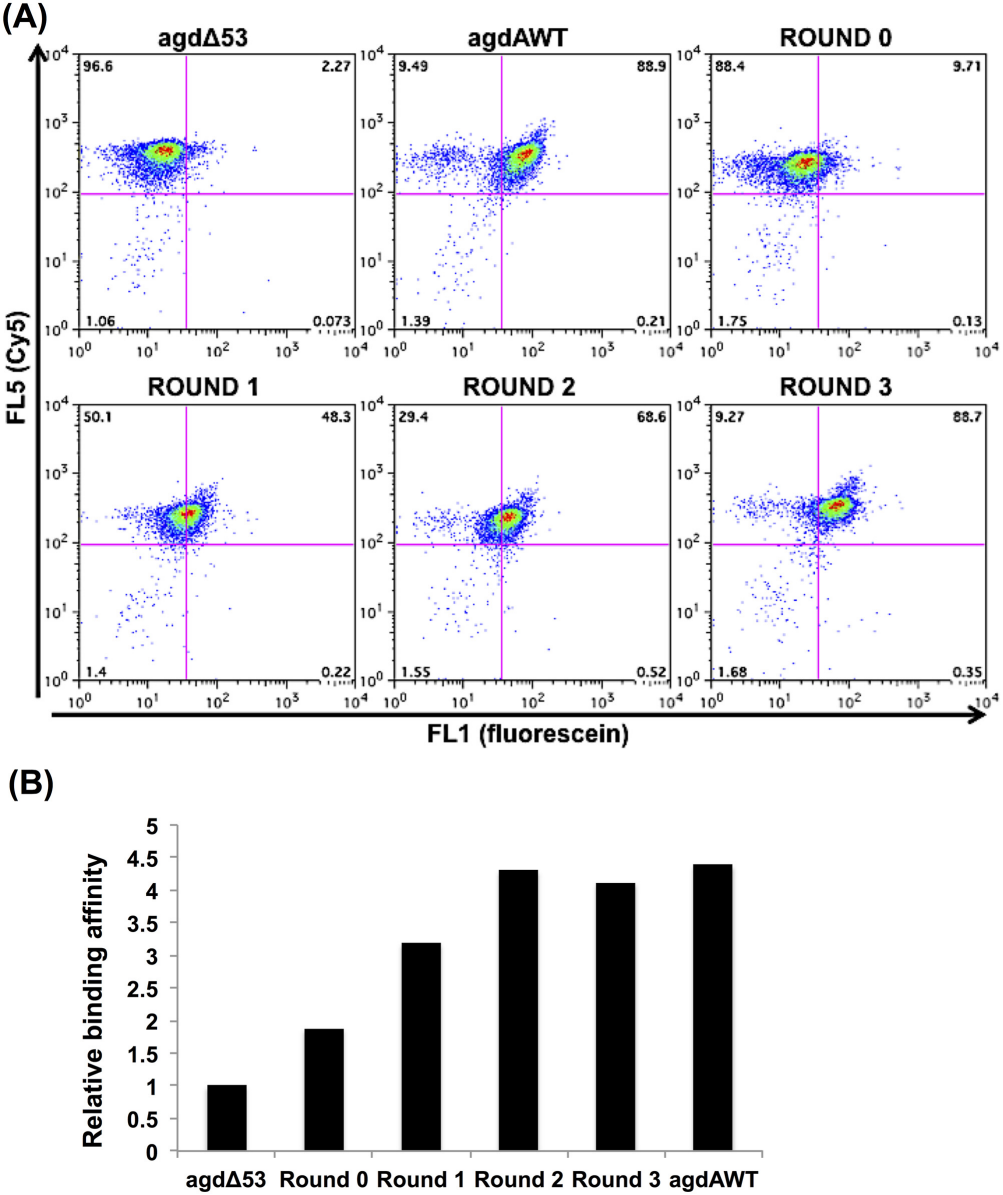
Flow cytometric analysis of selected DNA pools from gSELEX by using bead display. (A) Dot-plot of log fluorescence analysis. X-axis: quantified fluorescence intensity detected within the FL1 (fluorescein) channel; Y-axis: quantified fluorescence intensity detected within the FL5 (Cy5) channel. (B) Relative binding affinities measured against AmyR. The binding affinity was defined as the geometric mean of the intensity of FL1 divided by that of FL5 and the binding affinity of agdAΔ53 (a mutant of AmyR-binding DNA) against AmyR, which was set as 1.

First, we employed SELEX in an attempt to generate direct AmyR-binding profiles across the genome by using an *A*. *nidulans* genomic library (Fig 2). *A*. *nidulans* genomic DNA was fragmented to approximately 100 bp, ligated with linkers at both ends, and amplified using PCR. Next, this genomic library was used in 3 rounds of gSELEX selection against MalE::AmyR_1–411_. The pools from each selection round were labeled with biotin and Cy5 by using PCR and immobilized onto streptavidin-coated beads. Each set of these beads was next incubated with MalE::AmyR_1–411_, immunostained with a fluorescein-labeled anti-MBP antibody, and analyzed using flow cytometry to monitor the progress of the selection process (Fig 3). The fluorescein intensity increased with each round of selection (Fig 3A), although the relative binding activity was saturated at the agdAWT level by the second round (Fig 3B). The results suggest that the DNA fragments exhibiting high binding affinity for AmyR were successfully enriched using gSELEX.

Monitoring the bulk binding affinity in the selected pools is vital for assessing the quality of the library, optimizing the selection conditions, and evaluating the degree of enrichment of protein-bound DNA. An EMSA, which is frequently used to select protein-bound DNA for SELEX-Seq, can be used to directly monitor the complex formation as a shifted mobility [14]. By contrast, bead display used with flow cytometry allows the monitoring of the binding activity by measuring a fluorescent signal quantitatively [32].

**Fig 4:**
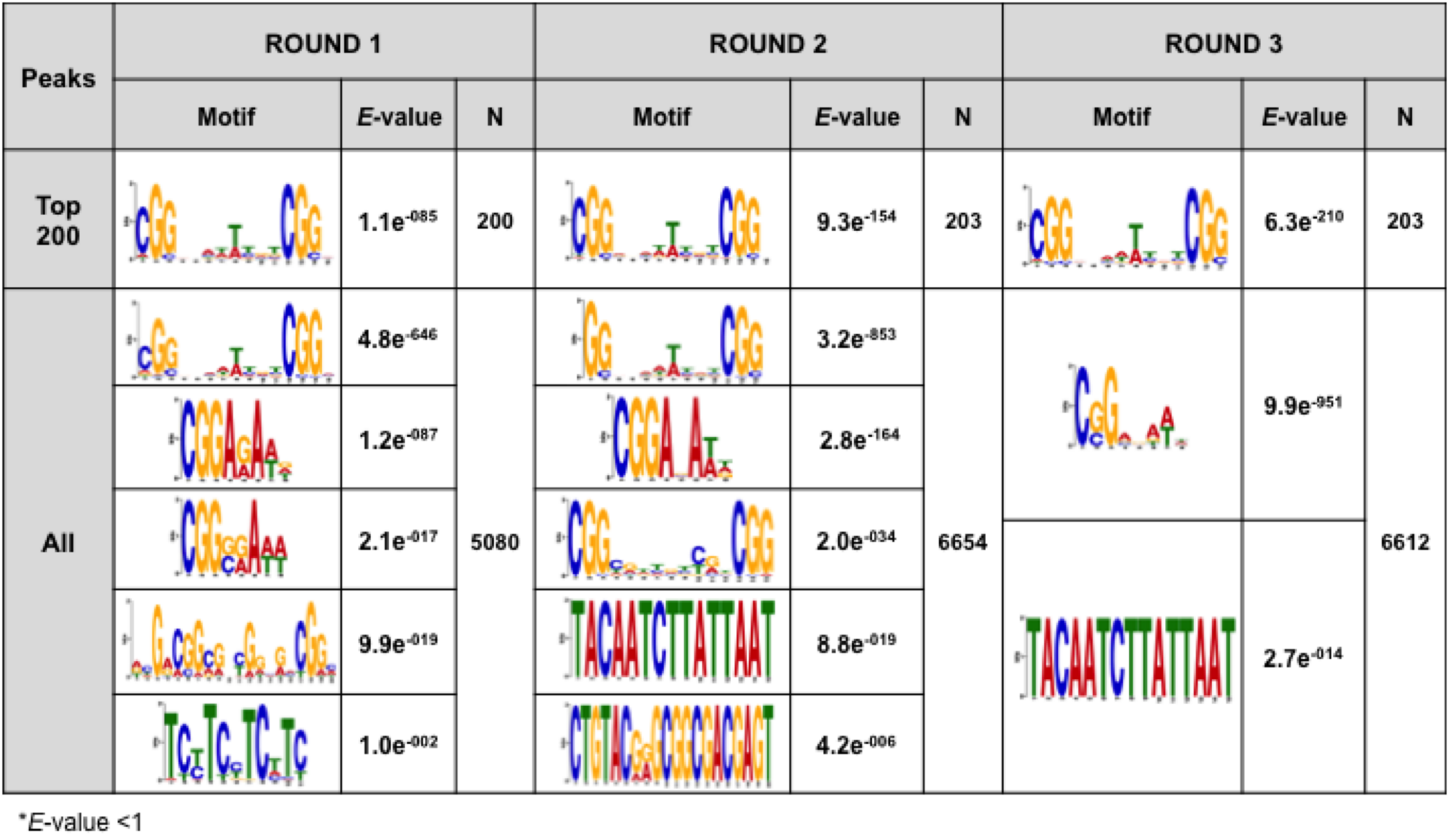
Analysis of AmyR-binding motifs. From the sequence of the peaks, 50-bp tags were extracted, and this was followed by *de novo* motif analysis of the AmyR-binding site with either all the extracted 50-bp tags or the top 200 tags ranked according to fold-enrichment. Only motifs featuring *E*-value < 1 are shown here. Motifs were identified using *MEME* (v 4.10.2).

All the DNA pools selected from the *A*. *nidulans* genomic library were sequenced using an Illumina HiSeq 2000 system for genome-wide identification of sites associated with AmyR. After the sequencing tags were mapped to the *A*. *nidulans* genome and the peaks with high numbers of tags were detected, 50 bp were extracted from the sequences of the peaks, following which *de novo* motif analysis of AmyR-binding sites was performed using either all the extracted 50-bp tags or the top 200 tags ranked according to fold-enrichment. Following the first round of selection, the canonical binding motif, CGGN_8_CGG, was clearly detected in a set containing the top 200 tags extracted (Fig 4). These results indicate that AmyR-binding sites were appropriately selected as early as after the first round of selection. Here, the fifth T in the N_8_ region was preferentially preserved in all the detected CGGN_8_CGG sequences. These results coincide well with the findings of our previous studies, in which AmyR-binding sites were screened using bead display [29, 32]. Moreover, other motifs containing a single CGG triplet were observed with the use of all tags. These results indicate that certain binding motifs exhibiting a low affinity for a target TF might also be identified using this method, because AmyR weakly binds to a single CGG triplet [26, 29, 32]. Conversely, the CGGN_8_CGG motif was not observed with all the tags in Rounds 2 and 3. We speculate that the AmyR concentration might have been extremely high in the binding reaction, and the high concentration could potentially facilitate the enrichment of fragments containing binding motifs that exhibit a low affinity for AmyR. Thus, in the case of all of the tags in Round 3, the binding motifs could have converged to a single CGG triplet. Supporting this view, the first motif observed in Round 3 with all tags showed very low *E*-value (Fig 4).

Each 50-bp tag from Round 3 was annotated using *A*. *nidulans* upstream1000, which contains the sequences of the regions 1000 bp upstream of the protein-coding sequence of *A*. *nidulans* genes. Notably, >70% of yeast transcriptional-regulator binding sites lie between 100 and 500 bp upstream of protein-coding sequences [35]. After the classification based on fold-enrichment, 2292 promoters were identified in the 1000-bp upstream region of *A*. *nidulans* genes, and 2263 distinct promoter regions were listed as candidate promoters under the control of AmyR (Table 1 and S1 Table). Previously, Nakamura *et al*. identified 8 amylolytic AmyR-regulated genes (*agdA*, *agdB*, *agdE*, *agdF*, *amyA*, *amyB*, *amyF*, and *glaB*) by using semi-quantitative RT-PCR analysis [36], and all of these genes were included among the candidates (Table 1 and S1 Table). Furthermore, the detected summits of the peaks were located in or near the CGGN_8_CGG motif in all 8 promoter regions of the previously reported amylolytic genes (S1 Fig); this indicates that the 8 upstream regions containing the CGGN_8_CGG motif were all preferentially selected when gSELEX was used. Our results agree well with the findings of the previous report [36], and thus underscore the robustness of our gSELEX-based selection system.

**Table 1:** *A*. *nidulans* promoter regions selected using gSELEX-Seq.

Group	Fold enrichment*	Selected in this study	Identified as gene regulated by AmyR in a previous study [36]
**1**	**12 and more**	**365**	***agdE*, *amyB*, *agdB*, *agdF***
**2**	**10 to 11.99**	**321**	***amyF*, *amyA***
**3**	**8 to 9.99**	**434**	***agdA***
**4**	**7 to 7.99**	**267**	
**5**	**6 to 6.99**	**313**	***glaB***
**6**	**5 to 5.99**	**304**	
**7**	**less than 5**	**288**	
**Total**	2292 (2263)**		

*Fold-enrichment values were obtained using *MACS* (v1.4.2).

**Value inside parenthesis indicates the number of distinct promoter sequences selected using gSELEX.

In the aforementioned selection, 2263 genes were obtained, but it is highly unlikely that AmyR regulates all of these genes in *A*. *nidulans*; thus, we speculate that the list includes several false-positive results. Furthermore, in gSELEX, the effect of chromosome structures in cells, for example the effect of methylation, is not considered. Therefore, the information gathered on binding regions from the results of gSELEX-Seq is, by itself, insufficient for accurately understanding the TF regulation system.

**Fig 5:**
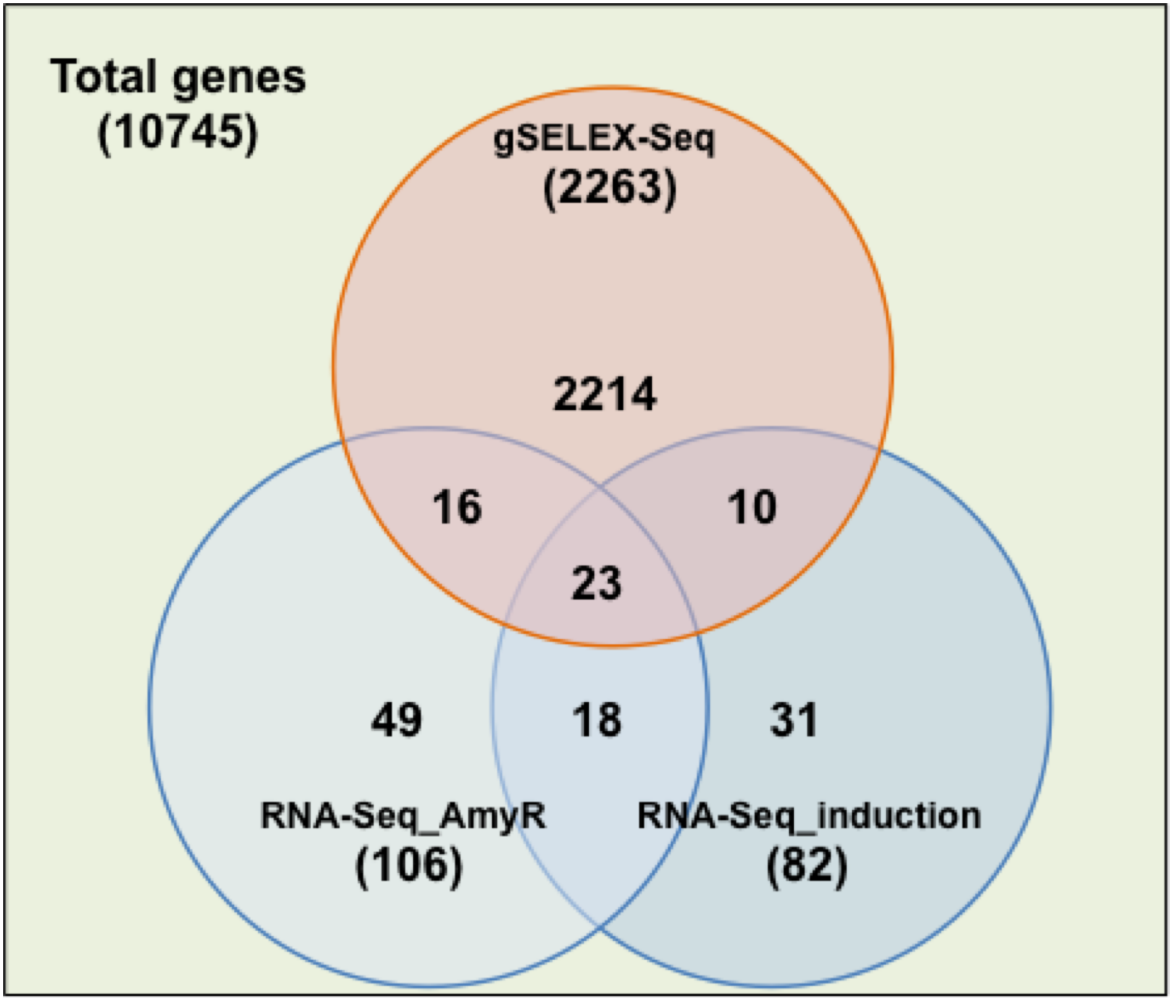
Venn diagram of the numbers of AmyR-related genes obtained from gSELEX-Seq and RNA-Seq. gSELEX-Seq: genes under the control of candidate AmyR-regulated promoters obtained using gSELEX; RNA-Seq_AmyR: DEGs in isomaltose treated BPU7 compared to treated Δ50 identified using RNA-Seq; RNA-Seq_induction: DEGs in isomaltose treated BPU7 compared to untreated one identified using RNA-Seq; Total genes: total genes in *A*. *nidulans* analyzed in this study. Values in parentheses indicate the total number of genes in each set.

Next, RNA-Seq analysis was performed using poly(A)-selected RNA samples from *A*. *nidulans* WT (BPU7) and an *amyR* deletant (Δ50), with or without isomaltose induction (S2 Fig and S2 Table). DEGs were detected and ranked based on the *P* values obtained by performing a statistical significance test, with filtering, by using *DESeq2* and *EdgeR* software; 106 genes were identified as AmyR-dependent DEGs, differentially expressed genes in isomaltose treated BPU7 compared to treated Δ50, and 82 genes were identified as induction-dependent DEGs, differentially expressed genes in isomaltose treated BPU7 compared to untreated one (S3 Table). By combining the two sets, we identified 41 genes—including *agdA*, *agdB*, *agdE*, *agdF*, *amyA*, *amyB*, and *amyF*—for which the response was both AmyR- and induction-dependent (Fig 5 and S4 Table). However, *glaB*, a glucoamylase gene that is induced by isomaltose and shows AmyR-dependence [36], was not included among the 41 DEGs that we identified. We speculate that this difference might be due to the induction time used: here, mycelia were harvested after induction for 4 h, whereas AmyR-dependent induction was detected at 3 h after isomaltose addition in the previous study [36].

The 41 DEGs were also compared with the promoter candidates obtained from gSELEX, and these candidates were found to include a subset 23 DEGs (Fig 5 and S4 Table). Thus, the percentage of this selection, 56% (23/41), was significantly higher than 21%, the percentage calculated for the number in the gSELEX-Seq dataset (2263) relative to the number in the dataset of Total genes (10745) (*p* < 0.05 Chi^2^ test). Notably, the promoter regions of most of the DEGs contained >1 CGGN_8_CGG motif (S4 Table). Although no CGGN_8_CGG motif was present in the 1000-bp upstream regions of AN0732, AN1797, AN3996, AN4586, AN8928, and AN9340, each summit of the detected peak was located in or near sequences similar to the CGGN_8_CGG motif (data not shown).

In RNA-Seq analysis, all identified DEGs should be genes that are affected, either primarily or secondarily, by the expression of the target TF. Thus, the TF would be expected to directly regulate only a subset of the DEGs. Our results strongly indicate that the 23 identified DEGs are regulated directly by AmyR; these 23 DEGs included all aforementioned amylolytic genes except *glaB* and 16 genes newly identified as AmyR-regulated genes (Fig 5 and S4 Table). Among the 16 genes, AN7662, AN8928, AN9340, and AN10081 have been well-characterized as a putative heme-containing metalloreductase (*freA*) [37], a putative ATP-binding cassette (ABC) transporter (*atrA*) [38], alpha-trehalase (*treA*) [39], and an alpha-ketoglutarate-dependent xanthine dioxygenase (*xanA*) [40], respectively (S4 Table). Intriguingly, *treA* is required for growth on trehalose used as a carbon source [39], which suggests that AmyR might be also involved in trehalose metabolism. The AmyR-dependency of these genes could be further confirmed by other methods such as quantitative RT-PCR.

Conversely, 18 DEGs, including *amyR*, were not included among the promoter candidates identified using gSELEX-Seq (Fig 5). One possibility is that AmyR indirectly regulates the expression of the 17 DEGs other than *amyR*. Another possibility is that the affinity between AmyR and the promoter regions is low. Notably, 12/17 DEGs contained no CGGN_8_CGG motif in the promoter regions (S4 Table), and AmyR binds—with comparatively lower affinity—to DNA sequences similar to this binding motif [29].

The expression profiles of AmyR-dependent genes in *Aspergillus* species have been widely reported [36, 41–44]. Yuan *et al*. conducted microarray analysis on an *A*. *niger amyR* deletant and identified AmyR-dependent and maltose-induced genes [42]. Coutinho *et al*. generated subsets consisting of putative amylolytic, pectinolytic, and xylanolytic/cellulolytic ORFs from 3 *Aspergillus* species, and analyzed them for the presence of AmyR-binding motifs [43]. These previous studies suggest the possibility that AmyR plays multiple and complex roles in *Aspergillus* species. The knowledge obtained in this study might facilitate an elucidation of the detailed functions of AmyR.

Generally, combined *in vitro*/*in vivo* methods can improve the insights obtained from each analysis. Dittmar et al. have developed a method for analysis of posttranscriptional network regulated by RNA binding proteins, ESPR1 and ESPR2, the epithelium-specific splicing regulatory proteins, combining using RNA-Seq and SELEX-Seq [45].

In this study, we successfully devised a novel transcriptome-analysis system that combines gSELEX-Seq and RNA-Seq. In this system, gSELEX-Seq provides information on the binding motifs of a target TF, as well as the candidate promoters controlled directly by the TF. Conversely, RNA-Seq is used for identifying the genes affected by the expression of a target TF. As mentioned above, the genes downstream of the candidate promoters from gSELEX-Seq and the DEGs from RNA-Seq can include false-positive results. Although it may be difficult to completely rule out the selection of false-positive genes, by obtaining the intersecting set of genes detected using both gSELEX-Seq and RNA-Seq, the genes regulated by a target TF can be identified with exceptionally high reliability.

In principle, this analysis can be applied to a wide variety of TFs from various organisms, including human, in order to identify several of the binding sites of the TFs and the genes that the TFs regulate across the genome. Since AmyR target genes are not well determined yet, it might be difficult to correctly evaluate our system based on only this study’s results. To expand the utility of the approach, we are currently employing this method in the identification of the target genes regulated by diverse TFs, such as TFs from plants and insects. In conclusion, this pipeline combining gSELEX-Seq and RNA-Seq is clearly a powerful tool for transcriptome analysis.

## Supporting Information

S1 Fig Summits of peaks detected using gSELEX-Seq in the promoter regions of the 8 amylolytic genes. gSELEX-Seq peaks were detected using *MACS* (v1.4.2). Blue squares indicate the summits of the peaks. Red square frames indicate CGGN8CGG.
(TIFF)

S2 Fig Scatter plots of relative expression values obtained using RNA-Seq. RNA-Seq analysis was performed using poly(A)-selected RNA samples from *A*. *nidulans* WT (BPU7) and an *amyR* deletant (Δ50), with or without isomaltose induction.
(TIFF)

S1 Table *A*. *nidulans* promoter regions selected by gSELEX-Seq. (DOCX)

S2 Table Correlation matrix of the expression level of *A*. *nidulans* genes detected using RNA-Seq. (DOCX)

S3 Table DEGs detected in RNA-Seq using RNA from *A*. *nidulans* BPU7 and Δ50, with or without induction by isomaltose. (DOCX)

S4 Table Overview of 41 genes detected as AmyR- and isomaltose induction-dependent DEGs. (DOCX)

## References

[1] Todeschini AL, Georges A, Veitia RA. Transcription factors: specific DNA binding and specific gene regulation. Trends Genet. 2014;30(6):211–9. 10.1016/j.tig.2014.04.002 . 24774859

[2] Wang J, Lu J, Gu G, Liu Y. In vitro DNA-binding profile of transcription factors: methods and new insights. J Endocrinol. 2011;210(1):15–27. 10.1530/JOE-11-0010 . 21389103

[3] Tuerk C, Gold L. Systematic evolution of ligands by exponential enrichment: RNA ligands to bacteriophage T4 DNA polymerase. Science. 1990;249(4968):505–10. . 2200121 10.1126/science.2200121

[4] Ellington AD, Szostak JW. In vitro selection of RNA molecules that bind specific ligands. Nature. 1990;346(6287):818–22. 10.1038/346818a0 . 1697402

[5] Oliphant AR, Brandl CJ, Struhl K. Defining the sequence specificity of DNA-binding proteins by selecting binding sites from random-sequence oligonucleotides: analysis of yeast GCN4 protein. Mol Cell Biol. 1989;9(7):2944–9. 2674675 10.1128/mcb.9.7.2944-2949.1989 PMC362762

[6] Papoulas O, Williams NG, Kingston RE. DNA binding activities of c-Myc purified from eukaryotic cells. J Biol Chem. 1992;267(15):10470–80. . 1587829

[7] Mitsui K, Tokuzawa Y, Itoh H, Segawa K, Murakami M, Takahashi K, et al The homeoprotein Nanog is required for maintenance of pluripotency in mouse epiblast and ES cells. Cell. 2003;113(5):631–42. . 12787504 10.1016/s0092-8674(03)00393-3

[8] Roulet E, Busso S, Camargo AA, Simpson AJ, Mermod N, Bucher P. High-throughput SELEX SAGE method for quantitative modeling of transcription-factor binding sites. Nat Biotechnol. 2002;20(8):831–5. 10.1038/nbt718 . 12101405

[9] Zykovich A, Korf I, Segal DJ. Bind-n-Seq: high-throughput analysis of in vitro protein-DNA interactions using massively parallel sequencing. Nucleic Acids Res. 2009;37(22):e151 10.1093/nar/gkp802 19843614 PMC2794170

[10] Jolma A, Kivioja T, Toivonen J, Cheng L, Wei G, Enge M, et al Multiplexed massively parallel SELEX for characterization of human transcription factor binding specificities. Genome Res. 2010;20(6):861–73. 10.1101/gr.100552.109 20378718 PMC2877582

[11] Jolma A, Yan J, Whitington T, Toivonen J, Nitta KR, Rastas P, et al DNA-binding specificities of human transcription factors. Cell. 2013;152(1–2):327–39. 10.1016/j.cell.2012.12.009 . 23332764

[12] Slattery M, Riley T, Liu P, Abe N, Gomez-Alcala P, Dror I, et al Cofactor binding evokes latent differences in DNA binding specificity between Hox proteins. Cell. 2011;147(6):1270–82. 10.1016/j.cell.2011.10.053 22153072 PMC3319069

[13] Wong D, Teixeira A, Oikonomopoulos S, Humburg P, Lone IN, Saliba D, et al Extensive characterization of NF-κB binding uncovers non-canonical motifs and advances the interpretation of genetic functional traits. Genome Biol. 2011;12(7):R70 10.1186/gb-2011-12-7-r70 21801342 PMC3218832

[14] Gu G, Wang T, Yang Y, Xu X, Wang J. An improved SELEX-Seq strategy for characterizing DNA-binding specificity of transcription factor: NF-κB as an example. PLoS One. 2013;8(10):e76109 10.1371/journal.pone.0076109 24130762 PMC3794954

[15] Shimada T, Fujita N, Maeda M, Ishihama A. Systematic search for the Cra-binding promoters using genomic SELEX system. Genes Cells. 2005;10(9):907–18. 10.1111/j.1365-2443.2005.00888.x . 16115199

[16] Singer BS, Shtatland T, Brown D, Gold L. Libraries for genomic SELEX. Nucleic Acids Res. 1997;25(4):781–6. 9016629 10.1093/nar/25.4.781 PMC146522

[17] Reiss DJ, Mobley HL. Determination of target sequence bound by PapX, repressor of bacterial motility, in flhD promoter using systematic evolution of ligands by exponential enrichment (SELEX) and high throughput sequencing. J Biol Chem. 2011;286(52):44726–38. 10.1074/jbc.M111.290684 22039053 PMC3247938

[18] Dror I, Golan T, Levy C, Rohs R, Mandel-Gutfreund Y. A widespread role of the motif environment in transcription factor binding across diverse protein families. Genome Res. 2015;25(9):1268–80. 10.1101/gr.184671.114 26160164 PMC4561487

[19] Rahmatallah Y, Emmert-Streib F, Glazko G. Gene set analysis approaches for RNA-seq data: performance evaluation and application guideline. Brief Bioinform. 2015 10.1093/bib/bbv069 . 26342128 PMC4870397

[20] Nagalakshmi U, Wang Z, Waern K, Shou C, Raha D, Gerstein M, et al The transcriptional landscape of the yeast genome defined by RNA sequencing. Science. 2008;320(5881):1344–9. 10.1126/science.1158441 18451266 PMC2951732

[21] Pan Q, Shai O, Lee LJ, Frey BJ, Blencowe BJ. Deep surveying of alternative splicing complexity in the human transcriptome by high-throughput sequencing. Nat Genet. 2008;40(12):1413–5. 10.1038/ng.259 . 18978789

[22] Wang B, Guo G, Wang C, Lin Y, Wang X, Zhao M, et al Survey of the transcriptome of Aspergillus oryzae via massively parallel mRNA sequencing. Nucleic Acids Res. 2010;38(15):5075–87. 10.1093/nar/gkq256 20392818 PMC2926611

[23] Cerqueira GC, Arnaud MB, Inglis DO, Skrzypek MS, Binkley G, Simison M, et al The Aspergillus Genome Database: multispecies curation and incorporation of RNA-Seq data to improve structural gene annotations. Nucleic Acids Res. 2014;42(Database issue):D705–10. 10.1093/nar/gkt1029 24194595 PMC3965050

[24] Petersen KL, Lehmbeck J, Christensen T. A new transcriptional activator for amylase genes in Aspergillus. Mol Gen Genet. 1999;262(4–5):668–76. . 10628849 10.1007/s004380051129

[25] Tani S, Katsuyama Y, Hayashi T, Suzuki H, Kato M, Gomi K, et al Characterization of the amyR gene encoding a transcriptional activator for the amylase genes in Aspergillus nidulans. Curr Genet. 2001;39(1):10–5. . 11318101 10.1007/s002940000175

[26] Tani S, Itoh T, Kato M, Kobayashi T, Tsukagoshi N. In vivo and in vitro analyses of the AmyR binding site of the Aspergillus nidulans agdA promoter; requirement of the CGG direct repeat for induction and high affinity binding of AmyR. Biosci Biotechnol Biochem. 2001;65(7):1568–74. . 11515540 10.1271/bbb.65.1568

[27] Ito T, Tani S, Itoh T, Tsukagoshi N, Kato M, Kobayashi T. Mode of AmyR binding to the CGGN8AGG sequence in the Aspergillus oryzae taaG2 promoter. Biosci Biotechnol Biochem. 2004;68(9):1906–11. . 15388966 10.1271/bbb.68.1906

[28] Rowlands RT, Turner G. Nuclear and extranuclear inheritance of oligomycin resistance in Aspergillus nidulans. Mol Gen Genet. 1973;126(3):201–16. . 4593756 10.1007/BF00267531

[29] Kojima T, Hashimoto Y, Kato M, Kobayashi T, Nakano H. High-throughput screening of DNA binding sites for transcription factor AmyR from Aspergillus nidulans using DNA beads display system. J Biosci Bioeng. 2010;109(6):519–25. S1389-1723(09)01056-1 [pii] 10.1016/j.jbiosc.2009.11.024 . 20471587

[30] Bradford MM. A rapid and sensitive method for the quantitation of microgram quantities of protein utilizing the principle of protein-dye binding. Anal Biochem. 1976;72:248–54. . 942051 10.1016/0003-2697(76)90527-3

[31] DYER P, NICHOLSON P, REZANOOR H, LUCAS J, PEBERDY J. 2-ALLELE HETEROTHALLISM IN TAPESIA-YALLUNDAE, THE TELEOMORPH OF THE CEREAL EYESPOT PATHOGEN PSEUDOCERCOSPORELLA-HERPOTRICHOIDES. Physiological and Molecular Plant Pathology. 1993;43(6):403–14. 10.1006/pmpp.1993.1068 WOS:A1993NZ55800002.

[32] Wang P, Kojima T, Kobayashi T, Nakano H. Comprehensive analysis of the DNA-binding specificity of an Aspergillus nidulans transcription factor, AmyR, using a bead display system. Biosci Biotechnol Biochem. 2012;76(6):1128–34. 10.1271/bbb.110949 . 22790934

[33] Zhang Y, Liu T, Meyer CA, Eeckhoute J, Johnson DS, Bernstein BE, et al Model-based analysis of ChIP-Seq (MACS). Genome Biol. 2008;9(9):R137 10.1186/gb-2008-9-9-r137 18798982 PMC2592715

[34] Riley TR, Slattery M, Abe N, Rastogi C, Liu D, Mann RS, et al SELEX-seq: a method for characterizing the complete repertoire of binding site preferences for transcription factor complexes. Methods Mol Biol. 2014;1196:255–78. 10.1007/978-1-4939-1242-1_16 25151169 PMC4265583

[35] Harbison CT, Gordon DB, Lee TI, Rinaldi NJ, Macisaac KD, Danford TW, et al Transcriptional regulatory code of a eukaryotic genome. Nature. 2004;431(7004):99–104. 10.1038/nature02800 15343339 PMC3006441

[36] Nakamura T, Maeda Y, Tanoue N, Makita T, Kato M, Kobayashi T. Expression profile of amylolytic genes in Aspergillus nidulans. Biosci Biotechnol Biochem. 2006;70(10):2363–70. 10.1271/bbb.50694 . 17031028

[37] Oberegger H, Schoeser M, Zadra I, Schrettl M, Parson W, Haas H. Regulation of freA, acoA, lysF, and cycA expression by iron availability in Aspergillus nidulans. Appl Environ Microbiol. 2002;68(11):5769–72. 12406779 10.1128/AEM.68.11.5769-5772.2002 PMC129941

[38] Del Sorbo G, Andrade AC, Van Nistelrooy JG, Van Kan JA, Balzi E, De Waard MA. Multidrug resistance in Aspergillus nidulans involves novel ATP-binding cassette transporters. Mol Gen Genet. 1997;254(4):417–26. . 9180695 10.1007/s004380050434

[39] d'Enfert C, Fontaine T. Molecular characterization of the Aspergillus nidulans treA gene encoding an acid trehalase required for growth on trehalose. Mol Microbiol. 1997;24(1):203–16. . 9140977 10.1046/j.1365-2958.1997.3131693.x

[40] Cultrone A, Scazzocchio C, Rochet M, Montero-Morán G, Drevet C, Fernández-Martín R. Convergent evolution of hydroxylation mechanisms in the fungal kingdom: molybdenum cofactor-independent hydroxylation of xanthine via alpha-ketoglutarate-dependent dioxygenases. Mol Microbiol. 2005;57(1):276–90. 10.1111/j.1365-2958.2005.04686.x . 15948966

[41] Levin AM, de Vries RP, Conesa A, de Bekker C, Talon M, Menke HH, et al Spatial differentiation in the vegetative mycelium of Aspergillus niger. Eukaryot Cell. 2007;6(12):2311–22. 10.1128/EC.00244-07 17951513 PMC2168252

[42] Yuan XL, van der Kaaij RM, van den Hondel CA, Punt PJ, van der Maarel MJ, Dijkhuizen L, et al Aspergillus niger genome-wide analysis reveals a large number of novel alpha-glucan acting enzymes with unexpected expression profiles. Mol Genet Genomics. 2008;279(6):545–61. 10.1007/s00438-008-0332-7 18320228 PMC2413074

[43] Coutinho PM, Andersen MR, Kolenova K, vanKuyk PA, Benoit I, Gruben BS, et al Post-genomic insights into the plant polysaccharide degradation potential of Aspergillus nidulans and comparison to Aspergillus niger and Aspergillus oryzae. Fungal Genet Biol. 2009;46 Suppl 1:S161–S9. . 19618505 10.1016/j.fgb.2008.07.020

[44] vanKuyk PA, Benen JA, Wösten HA, Visser J, de Vries RP. A broader role for AmyR in Aspergillus niger: regulation of the utilisation of D-glucose or D-galactose containing oligo- and polysaccharides. Appl Microbiol Biotechnol. 2012;93(1):285–93. 10.1007/s00253-011-3550-6 21874276 PMC3251782

[45] Dittmar KA, Jiang P, Park JW, Amirikian K, Wan J, Shen S, et al Genome-wide determination of a broad ESRP-regulated posttranscriptional network by high-throughput sequencing. Mol Cell Biol. 2012;32(8):1468–82. 10.1128/MCB.06536-11 22354987 PMC3318588

