# Supplementary figures and images for: A Robust Analytical Pipeline for Genome-Wide Identification of the Genes Regulated by a Transcription Factor: Combinatorial Analysis Performed Using gSELEX-Seq and RNA-Seq
Source: PLoS One. 2016 Jul 13;11(7):e0159011. doi: 10.1371/journal.pone.0159011 (PMC4943734; doi:10.1371/journal.pone.0159011)

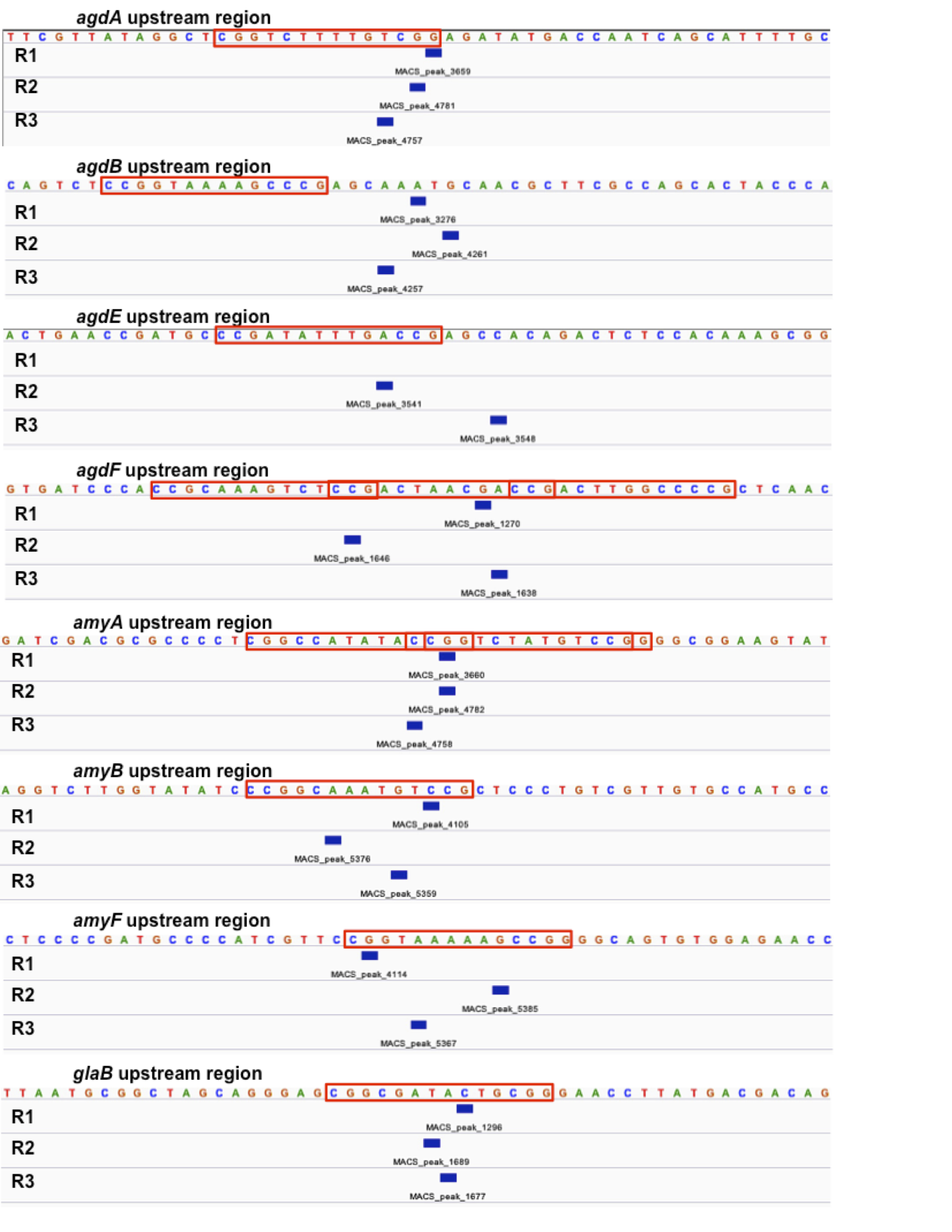

Supplement: S1 Fig — gSELEX-Seq peaks were detected using MACS (v1.4.2). Blue squares indicate the summits of the peaks. Red square frames indicate CGGN8CGG. (TIFF) [file pone.0159011.s001.tiff]

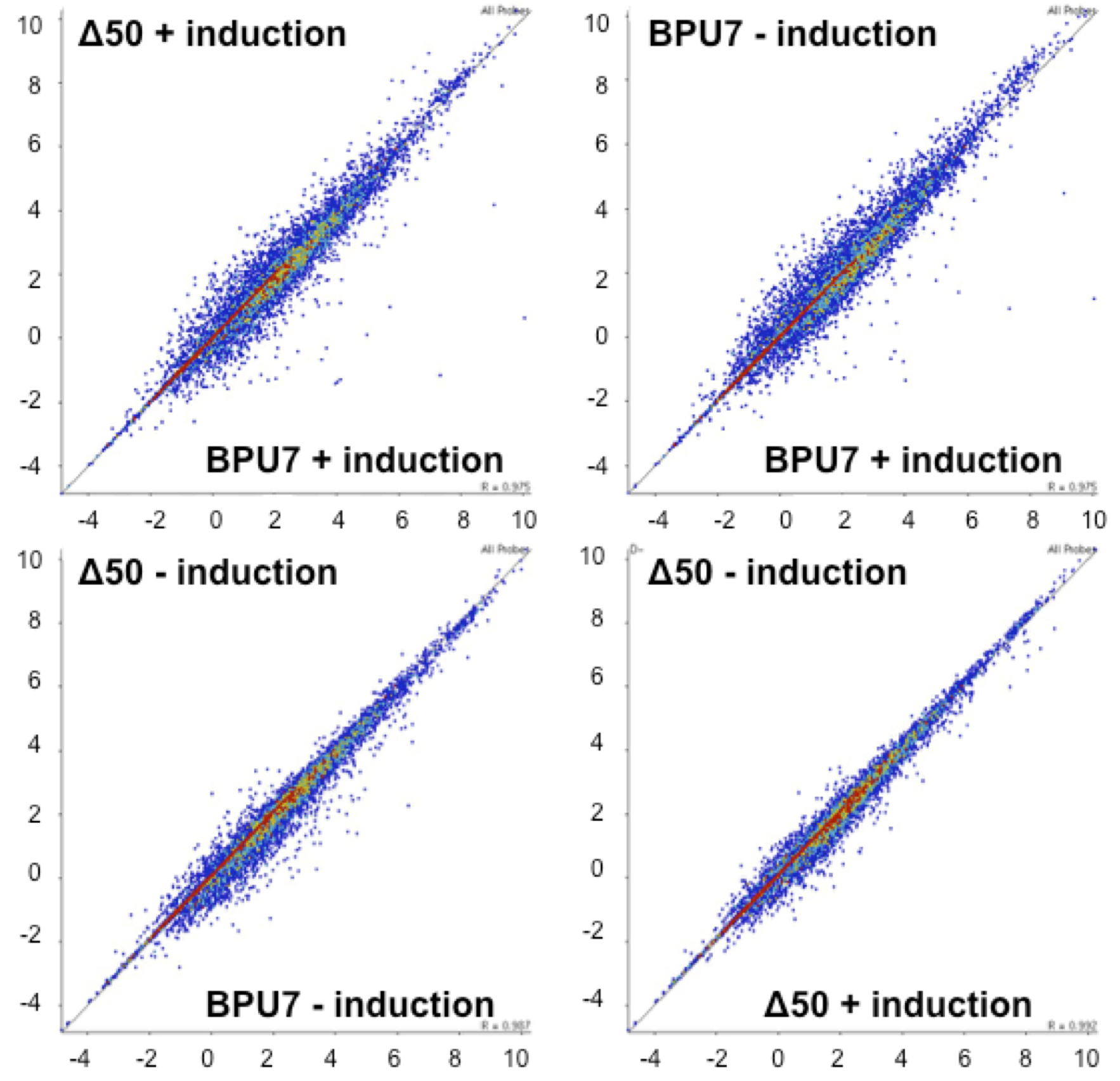

Supplement: S2 Fig — RNA-Seq analysis was performed using poly(A)-selected RNA samples from A. nidulans WT (BPU7) and an amyR deletant (Δ50), with or without isomaltose induction. (TIFF) [file pone.0159011.s002.tiff]
