# Supplementary material for: A Robust Analytical Pipeline for Genome-Wide Identification of the Genes Regulated by a Transcription Factor: Combinatorial Analysis Performed Using gSELEX-Seq and RNA-Seq
Source: PLoS One. 2016 Jul 13;11(7):e0159011. doi: 10.1371/journal.pone.0159011 (PMC4943734; doi:10.1371/journal.pone.0159011)
Supplement: S1 Table — (DOCX) [file pone.0159011.s003.docx]

**S1 Table. *A. nidulans* promoter regions selected by gSELEX-Seq**

| **Query ID** | **Subject ID** |
| --- | --- |
| **ChrIV_A_nidulans_FGSC_A4:1709146-1709196** | **AN7491** |
| **ChrVIII_A_nidulans_FGSC_A4:923789-923839** | **AN1293** |
| **ChrVII_A_nidulans_FGSC_A4:1457774-1457824** | **AN1643** |
| **ChrVI_A_nidulans_FGSC_A4:40724-40774** | **AN9225** |
| **ChrIII_A_nidulans_FGSC_A4:1160125-1160175** | **AN4731** |
| **ChrIV_A_nidulans_FGSC_A4:347239-347289** | **AN7307** |
| **ChrVI_A_nidulans_FGSC_A4:398326-398376** | **AN11177** |
| **ChrII_A_nidulans_FGSC_A4:360000-360050** | **AN7972** |
| **ChrII_A_nidulans_FGSC_A4:360000-360050** | **AN7971** |
| **ChrVIII_A_nidulans_FGSC_A4:843346-843396** | **AN1320** |
| **ChrIII_A_nidulans_FGSC_A4:3118103-3118153** | **AN8689** |
| **ChrVII_A_nidulans_FGSC_A4:571729-571779** | **AN11145** |
| **ChrV_A_nidulans_FGSC_A4:1930782-1930832** | **AN5708** |
| **ChrI_A_nidulans_FGSC_A4:2368645-2368695** | **AN6524** |
| **ChrIV_A_nidulans_FGSC_A4:681578-681628** | **AN7208** |
| **ChrVIII_A_nidulans_FGSC_A4:1943337-1943387** | **AN0964** |
| **ChrII_A_nidulans_FGSC_A4:241929-241979** | **AN7923** |
| **ChrIII_A_nidulans_FGSC_A4:2740549-2740599** | **AN8812** |
| **ChrII_A_nidulans_FGSC_A4:2845807-2845857** | **AN3863** |
| **ChrI_A_nidulans_FGSC_A4:2276719-2276769** | **AN6488** |
| **ChrI_A_nidulans_FGSC_A4:2276719-2276769** | **AN6490** |
| **ChrII_A_nidulans_FGSC_A4:1667696-1667746** | **AN4235** |
| **ChrII_A_nidulans_FGSC_A4:1667696-1667746** | **AN10526** |
| **ChrV_A_nidulans_FGSC_A4:2733980-2734030** | **AN5463** |
| **ChrIV_A_nidulans_FGSC_A4:53062-53112** | **AN7406** |
| **ChrII_A_nidulans_FGSC_A4:1966207-1966257** | **AN12099** |
| **ChrII_A_nidulans_FGSC_A4:2309896-2309946** | **AN4025** |
| **ChrVIII_A_nidulans_FGSC_A4:1261670-1261720** | **AN1185** |
| **ChrIII_A_nidulans_FGSC_A4:2983166-2983216** | **AN8737** |
| **ChrVIII_A_nidulans_FGSC_A4:2006456-2006506** | **AN0941** |
| **ChrV_A_nidulans_FGSC_A4:2370757-2370807** | **AN5571** |
| **ChrI_A_nidulans_FGSC_A4:3552198-3552248** | **AN6915** |
| **ChrVIII_A_nidulans_FGSC_A4:2202154-2202204** | **AN0876** |
| **ChrVI_A_nidulans_FGSC_A4:134472-134522** | **AN9199** |
| **ChrV_A_nidulans_FGSC_A4:2466933-2466983** | **AN5540** |
| **ChrIV_A_nidulans_FGSC_A4:85807-85857** | **AN7396** |
| **ChrVII_A_nidulans_FGSC_A4:1452500-1452550** | **AN1641** |
| **ChrI_A_nidulans_FGSC_A4:1948407-1948457** | **AN10742** |
| **ChrI_A_nidulans_FGSC_A4:114913-114963** | **AN6457** |
| **ChrVIII_A_nidulans_FGSC_A4:4265047-4265097** | **AN0217** |
| **ChrVII_A_nidulans_FGSC_A4:337997-338047** | **AN8950** |
| **ChrVII_A_nidulans_FGSC_A4:460351-460401** | **AN9425** |
| **ChrII_A_nidulans_FGSC_A4:402154-402204** | **AN7985** |
| **ChrVII_A_nidulans_FGSC_A4:538247-538297** | **AN8891** |
| **ChrVI_A_nidulans_FGSC_A4:412892-412942** | **AN9108** |
| **ChrV_A_nidulans_FGSC_A4:243218-243268** | **AN8366** |
| **ChrVI_A_nidulans_FGSC_A4:1331647-1331697** | **AN3252** |
| **ChrI_A_nidulans_FGSC_A4:889821-889871** | **AN6216** |
| **ChrVII_A_nidulans_FGSC_A4:2064049-2064099** | **AN1850** |
| **ChrVII_A_nidulans_FGSC_A4:3955170-3955220** | **AN2441** |
| **ChrIV_A_nidulans_FGSC_A4:40690-40740** | **AN7411** |
| **ChrIV_A_nidulans_FGSC_A4:40690-40740** | **AN7412** |
| **ChrVIII_A_nidulans_FGSC_A4:1584038-1584088** | **AN11840** |
| **ChrII_A_nidulans_FGSC_A4:3106617-3106667** | **AN3769** |
| **ChrIII_A_nidulans_FGSC_A4:2164715-2164765** | **AN4395** |
| **ChrI_A_nidulans_FGSC_A4:2468806-2468856** | **AN6563** |
| **ChrV_A_nidulans_FGSC_A4:375253-375303** | **AN8404** |
| **ChrIII_A_nidulans_FGSC_A4:2819134-2819184** | **AN8782** |
| **ChrI_A_nidulans_FGSC_A4:2415267-2415317** | **AN6540** |
| **ChrI_A_nidulans_FGSC_A4:2415267-2415317** | **AN6541** |
| **ChrIII_A_nidulans_FGSC_A4:552803-552853** | **AN11768** |
| **ChrVI_A_nidulans_FGSC_A4:2097023-2097073** | **AN11696** |
| **ChrI_A_nidulans_FGSC_A4:2889875-2889925** | **AN6699** |
| **ChrVIII_A_nidulans_FGSC_A4:4138337-4138387** | **AN0253** |
| **ChrVIII_A_nidulans_FGSC_A4:4138337-4138387** | **AN0252** |
| **ChrV_A_nidulans_FGSC_A4:1830911-1830961** | **AN5742** |
| **ChrV_A_nidulans_FGSC_A4:2964781-2964831** | **AN5390** |
| **ChrV_A_nidulans_FGSC_A4:2964781-2964831** | **AN5391** |
| **ChrI_A_nidulans_FGSC_A4:2009909-2009959** | **AN5853** |
| **ChrI_A_nidulans_FGSC_A4:2009909-2009959** | **AN5854** |
| **ChrII_A_nidulans_FGSC_A4:2691246-2691296** | **AN10489** |
| **ChrIV_A_nidulans_FGSC_A4:144920-144970** | **AN7375** |
| **ChrIV_A_nidulans_FGSC_A4:144920-144970** | **AN7374** |
| **ChrVII_A_nidulans_FGSC_A4:4022426-4022476** | **AN2463** |
| **ChrVII_A_nidulans_FGSC_A4:4022426-4022476** | **AN10306** |
| **ChrII_A_nidulans_FGSC_A4:229042-229092** | **AN7916** |
| **ChrV_A_nidulans_FGSC_A4:2597879-2597929** | **AN5501** |
| **ChrII_A_nidulans_FGSC_A4:198672-198722** | **AN7905** |
| **ChrIV_A_nidulans_FGSC_A4:1182781-1182831** | **AN7055** |
| **ChrIV_A_nidulans_FGSC_A4:1875013-1875063** | **AN7537** |
| **ChrVII_A_nidulans_FGSC_A4:724945-724995** | **AN1419** |
| **ChrVIII_A_nidulans_FGSC_A4:2681261-2681311** | **AN0722** |
| **ChrII_A_nidulans_FGSC_A4:1502821-1502871** | **AN4288** |
| **ChrVI_A_nidulans_FGSC_A4:3323229-3323279** | **AN2630** |
| **ChrV_A_nidulans_FGSC_A4:1712288-1712338** | **AN10708** |
| **ChrV_A_nidulans_FGSC_A4:1712288-1712338** | **AN5784** |
| **ChrII_A_nidulans_FGSC_A4:2517229-2517279** | **AN3956** |
| **ChrVI_A_nidulans_FGSC_A4:343740-343790** | **AN9133** |
| **ChrIV_A_nidulans_FGSC_A4:286971-287021** | **AN7331** |
| **ChrIV_A_nidulans_FGSC_A4:286971-287021** | **AN7332** |
| **ChrI_A_nidulans_FGSC_A4:3600406-3600456** | **AN10868** |
| **ChrII_A_nidulans_FGSC_A4:368269-368319** | **AN7973** |
| **ChrVII_A_nidulans_FGSC_A4:1197414-1197464** | **AN12137** |
| **ChrVIII_A_nidulans_FGSC_A4:1518188-1518238** | **AN1099** |
| **ChrIII_A_nidulans_FGSC_A4:1949037-1949087** | **AN9433** |
| **ChrIV_A_nidulans_FGSC_A4:954984-955034** | **AN7119** |
| **ChrIV_A_nidulans_FGSC_A4:954984-955034** | **AN7120** |
| **ChrIV_A_nidulans_FGSC_A4:2000002-2000052** | **AN11756** |
| **ChrIV_A_nidulans_FGSC_A4:2000002-2000052** | **AN10948** |
| **ChrI_A_nidulans_FGSC_A4:1775640-1775690** | **AN5930** |
| **ChrIII_A_nidulans_FGSC_A4:2720818-2720868** | **AN8815** |
| **ChrII_A_nidulans_FGSC_A4:1206782-1206832** | **AN8229** |
| **ChrVIII_A_nidulans_FGSC_A4:653746-653796** | **AN1394** |
| **ChrVII_A_nidulans_FGSC_A4:3519267-3519317** | **AN10285** |
| **ChrVII_A_nidulans_FGSC_A4:3519267-3519317** | **AN2311** |
| **ChrVI_A_nidulans_FGSC_A4:2215363-2215413** | **AN2969** |
| **ChrV_A_nidulans_FGSC_A4:2491628-2491678** | **AN5533** |
| **ChrVIII_A_nidulans_FGSC_A4:3020048-3020098** | **AN0605** |
| **ChrIV_A_nidulans_FGSC_A4:2739964-2740014** | **AN7804** |
| **ChrII_A_nidulans_FGSC_A4:3942482-3942532** | **AN10421** |
| **ChrIV_A_nidulans_FGSC_A4:1533768-1533818** | **AN7435** |
| **ChrVI_A_nidulans_FGSC_A4:3071974-3072024** | **AN2710** |
| **ChrII_A_nidulans_FGSC_A4:2571043-2571093** | **AN3944** |
| **ChrVII_A_nidulans_FGSC_A4:4493286-4493336** | **AN2603** |
| **ChrVI_A_nidulans_FGSC_A4:3221435-3221485** | **AN2664** |
| **ChrII_A_nidulans_FGSC_A4:1709480-1709530** | **AN4220** |
| **ChrII_A_nidulans_FGSC_A4:1709480-1709530** | **AN4219** |
| **ChrVII_A_nidulans_FGSC_A4:3829715-3829765** | **AN11337** |
| **ChrIV_A_nidulans_FGSC_A4:1886128-1886178** | **AN7541** |
| **ChrV_A_nidulans_FGSC_A4:2418584-2418634** | **AN5556** |
| **ChrIII_A_nidulans_FGSC_A4:284991-285041** | **AN11778** |
| **ChrVI_A_nidulans_FGSC_A4:1950534-1950584** | **AN3067** |
| **ChrII_A_nidulans_FGSC_A4:221548-221598** | **AN7913** |
| **ChrII_A_nidulans_FGSC_A4:3947942-3947992** | **AN12192** |
| **ChrVI_A_nidulans_FGSC_A4:828145-828195** | **AN3402** |
| **ChrII_A_nidulans_FGSC_A4:144710-144760** | **AN7887** |
| **ChrI_A_nidulans_FGSC_A4:1559628-1559678** | **AN5996** |
| **ChrII_A_nidulans_FGSC_A4:846629-846679** | **AN12449** |
| **ChrV_A_nidulans_FGSC_A4:447758-447808** | **AN8424** |
| **ChrVI_A_nidulans_FGSC_A4:1052930-1052980** | **AN3338** |
| **ChrVIII_A_nidulans_FGSC_A4:392874-392924** | **AN9353** |
| **ChrVIII_A_nidulans_FGSC_A4:392874-392924** | **AN9354** |
| **ChrI_A_nidulans_FGSC_A4:10028-10078** | **AN10813** |
| **ChrVI_A_nidulans_FGSC_A4:234918-234968** | **AN9164** |
| **ChrV_A_nidulans_FGSC_A4:583399-583449** | **AN8467** |
| **ChrVIII_A_nidulans_FGSC_A4:2245752-2245802** | **AN0861** |
| **ChrVIII_A_nidulans_FGSC_A4:2245752-2245802** | **AN0862** |
| **ChrVII_A_nidulans_FGSC_A4:4190789-4190839** | **AN2511** |
| **ChrVII_A_nidulans_FGSC_A4:4190789-4190839** | **AN10315** |
| **ChrVIII_A_nidulans_FGSC_A4:3566496-3566546** | **AN0432** |
| **ChrII_A_nidulans_FGSC_A4:1198568-1198618** | **AN8224** |
| **ChrII_A_nidulans_FGSC_A4:1198568-1198618** | **AN8225** |
| **ChrIV_A_nidulans_FGSC_A4:1337480-1337530** | **AN7007** |
| **ChrIV_A_nidulans_FGSC_A4:1337480-1337530** | **AN7008** |
| **ChrIII_A_nidulans_FGSC_A4:1664579-1664629** | **AN4558** |
| **ChrII_A_nidulans_FGSC_A4:1108903-1108953** | **AN8199** |
| **ChrVI_A_nidulans_FGSC_A4:1967231-1967281** | **AN3062** |
| **ChrVI_A_nidulans_FGSC_A4:1967231-1967281** | **AN3063** |
| **ChrI_A_nidulans_FGSC_A4:356996-357046** | **AN9497** |
| **ChrI_A_nidulans_FGSC_A4:3137985-3138035** | **AN6778** |
| **ChrV_A_nidulans_FGSC_A4:70417-70467** | **AN8308** |
| **ChrV_A_nidulans_FGSC_A4:552646-552696** | **AN11875** |
| **ChrV_A_nidulans_FGSC_A4:2004875-2004925** | **AN5676** |
| **ChrIII_A_nidulans_FGSC_A4:1828462-1828512** | **AN11910** |
| **ChrIII_A_nidulans_FGSC_A4:2340327-2340377** | **AN4341** |
| **ChrIII_A_nidulans_FGSC_A4:2340327-2340377** | **AN4342** |
| **ChrIII_A_nidulans_FGSC_A4:3021053-3021103** | **AN12241** |
| **ChrIV_A_nidulans_FGSC_A4:2642941-2642991** | **AN7766** |
| **ChrV_A_nidulans_FGSC_A4:2208465-2208515** | **AN5614** |
| **ChrVI_A_nidulans_FGSC_A4:2880574-2880624** | **AN2762** |
| **ChrV_A_nidulans_FGSC_A4:3185068-3185118** | **AN5312** |
| **ChrVIII_A_nidulans_FGSC_A4:1659151-1659201** | **AN1055** |
| **ChrIII_A_nidulans_FGSC_A4:2084989-2085039** | **AN4425** |
| **ChrIII_A_nidulans_FGSC_A4:2084989-2085039** | **AN4424** |
| **ChrIV_A_nidulans_FGSC_A4:469126-469176** | **AN7274** |
| **ChrI_A_nidulans_FGSC_A4:2273960-2274010** | **AN6487** |
| **ChrI_A_nidulans_FGSC_A4:2346521-2346571** | **AN6517** |
| **ChrVI_A_nidulans_FGSC_A4:2262779-2262829** | **AN2956** |
| **ChrIII_A_nidulans_FGSC_A4:2077984-2078034** | **AN4427** |
| **ChrV_A_nidulans_FGSC_A4:590599-590649** | **AN8469** |
| **ChrIII_A_nidulans_FGSC_A4:182447-182497** | **AN10612** |
| **ChrVIII_A_nidulans_FGSC_A4:1911015-1911065** | **AN0976** |
| **ChrVIII_A_nidulans_FGSC_A4:337257-337307** | **AN9340** |
| **ChrII_A_nidulans_FGSC_A4:1161363-1161413** | **AN8211** |
| **ChrV_A_nidulans_FGSC_A4:1483553-1483603** | **AN5206** |
| **ChrVIII_A_nidulans_FGSC_A4:978457-978507** | **AN1274** |
| **ChrVIII_A_nidulans_FGSC_A4:978457-978507** | **AN1275** |
| **ChrII_A_nidulans_FGSC_A4:2401043-2401093** | **AN3992** |
| **ChrVI_A_nidulans_FGSC_A4:977213-977263** | **AN3357** |
| **ChrVIII_A_nidulans_FGSC_A4:4806585-4806635** | **AN0034** |
| **ChrIII_A_nidulans_FGSC_A4:2776007-2776057** | **AN8798** |
| **ChrI_A_nidulans_FGSC_A4:1984349-1984399** | **AN5860** |
| **ChrVIII_A_nidulans_FGSC_A4:86910-86960** | **AN11206** |
| **ChrVIII_A_nidulans_FGSC_A4:86910-86960** | **AN9259** |
| **ChrIII_A_nidulans_FGSC_A4:2154213-2154263** | **AN4401** |
| **ChrVII_A_nidulans_FGSC_A4:1800713-1800763** | **AN1753** |
| **ChrVII_A_nidulans_FGSC_A4:1800713-1800763** | **AN1752** |
| **ChrII_A_nidulans_FGSC_A4:3333780-3333830** | **AN3702** |
| **ChrII_A_nidulans_FGSC_A4:3333780-3333830** | **AN3701** |
| **ChrI_A_nidulans_FGSC_A4:1099010-1099060** | **AN6147** |
| **ChrI_A_nidulans_FGSC_A4:1099010-1099060** | **AN6146** |
| **ChrVIII_A_nidulans_FGSC_A4:1214658-1214708** | **AN1200** |
| **ChrVIII_A_nidulans_FGSC_A4:1214658-1214708** | **AN1201** |
| **ChrVI_A_nidulans_FGSC_A4:2250854-2250904** | **AN10371** |
| **ChrV_A_nidulans_FGSC_A4:1420551-1420601** | **AN12033** |
| **ChrV_A_nidulans_FGSC_A4:3099683-3099733** | **AN5340** |
| **ChrII_A_nidulans_FGSC_A4:422394-422444** | **AN7988** |
| **ChrII_A_nidulans_FGSC_A4:1312844-1312894** | **AN12206** |
| **ChrI_A_nidulans_FGSC_A4:1009272-1009322** | **AN6180** |
| **ChrVI_A_nidulans_FGSC_A4:1399931-1399981** | **AN3229** |
| **ChrVI_A_nidulans_FGSC_A4:1399931-1399981** | **AN3230** |
| **ChrIII_A_nidulans_FGSC_A4:939529-939579** | **AN4804** |
| **ChrVI_A_nidulans_FGSC_A4:48141-48191** | **AN11200** |
| **ChrVI_A_nidulans_FGSC_A4:1169227-1169277** | **AN3307** |
| **ChrV_A_nidulans_FGSC_A4:147008-147058** | **AN8328** |
| **ChrV_A_nidulans_FGSC_A4:2131551-2131601** | **AN5637** |
| **ChrV_A_nidulans_FGSC_A4:2131551-2131601** | **AN5638** |
| **ChrV_A_nidulans_FGSC_A4:1322535-1322585** | **AN10640** |
| **ChrII_A_nidulans_FGSC_A4:2839215-2839265** | **AN3864** |
| **ChrI_A_nidulans_FGSC_A4:2586880-2586930** | **AN6599** |
| **ChrI_A_nidulans_FGSC_A4:2586880-2586930** | **AN6598** |
| **ChrI_A_nidulans_FGSC_A4:3674243-3674293** | **AN6951** |
| **ChrI_A_nidulans_FGSC_A4:583551-583601** | **AN6311** |
| **ChrII_A_nidulans_FGSC_A4:973632-973682** | **AN8161** |
| **ChrIII_A_nidulans_FGSC_A4:2105077-2105127** | **AN4419** |
| **ChrVIII_A_nidulans_FGSC_A4:4401021-4401071** | **AN0165** |
| **ChrVIII_A_nidulans_FGSC_A4:4401021-4401071** | **AN11849** |
| **ChrVI_A_nidulans_FGSC_A4:3010667-3010717** | **AN2729** |
| **ChrVI_A_nidulans_FGSC_A4:3010667-3010717** | **AN2728** |
| **ChrIII_A_nidulans_FGSC_A4:852391-852441** | **AN10599** |
| **ChrIII_A_nidulans_FGSC_A4:852391-852441** | **AN4829** |
| **ChrVIII_A_nidulans_FGSC_A4:1828741-1828791** | **AN1004** |
| **ChrVI_A_nidulans_FGSC_A4:2527709-2527759** | **AN2875** |
| **ChrIV_A_nidulans_FGSC_A4:1410704-1410754** | **AN6979** |
| **ChrI_A_nidulans_FGSC_A4:1923579-1923629** | **AN5884** |
| **ChrI_A_nidulans_FGSC_A4:1923579-1923629** | **AN5883** |
| **ChrIV_A_nidulans_FGSC_A4:411095-411145** | **AN7286** |
| **ChrIV_A_nidulans_FGSC_A4:411095-411145** | **AN7287** |
| **ChrVIII_A_nidulans_FGSC_A4:2657229-2657279** | **AN9436** |
| **ChrVIII_A_nidulans_FGSC_A4:2657229-2657279** | **AN9437** |
| **ChrII_A_nidulans_FGSC_A4:1077878-1077928** | **AN8192** |
| **ChrII_A_nidulans_FGSC_A4:2510135-2510185** | **AN3960** |
| **ChrVII_A_nidulans_FGSC_A4:3507467-3507517** | **AN2306** |
| **ChrV_A_nidulans_FGSC_A4:1874059-1874109** | **AN5728** |
| **ChrIII_A_nidulans_FGSC_A4:2868710-2868760** | **AN11119** |
| **ChrVI_A_nidulans_FGSC_A4:3316162-3316212** | **AN2633** |
| **ChrVII_A_nidulans_FGSC_A4:381936-381986** | **AN8937** |
| **ChrVIII_A_nidulans_FGSC_A4:2690800-2690850** | **AN0715** |
| **ChrIII_A_nidulans_FGSC_A4:749692-749742** | **AN4859** |
| **ChrVII_A_nidulans_FGSC_A4:685986-686036** | **AN10206** |
| **ChrVII_A_nidulans_FGSC_A4:3863858-3863908** | **AN2418** |
| **ChrVIII_A_nidulans_FGSC_A4:1495498-1495548** | **AN1104** |
| **ChrVIII_A_nidulans_FGSC_A4:3886259-3886309** | **AN0328** |
| **ChrV_A_nidulans_FGSC_A4:671348-671398** | **AN8490** |
| **ChrVIII_A_nidulans_FGSC_A4:1535602-1535652** | **AN1095** |
| **ChrI_A_nidulans_FGSC_A4:3281185-3281235** | **AN11987** |
| **ChrIII_A_nidulans_FGSC_A4:1880956-1881006** | **AN4488** |
| **ChrIII_A_nidulans_FGSC_A4:1880956-1881006** | **AN4489** |
| **ChrII_A_nidulans_FGSC_A4:1908007-1908057** | **AN4162** |
| **ChrVIII_A_nidulans_FGSC_A4:4301410-4301460** | **AN0204** |
| **ChrVII_A_nidulans_FGSC_A4:821517-821567** | **AN1452** |
| **ChrVII_A_nidulans_FGSC_A4:821517-821567** | **AN1453** |
| **ChrIII_A_nidulans_FGSC_A4:455531-455581** | **AN4956** |
| **ChrIII_A_nidulans_FGSC_A4:455531-455581** | **AN4957** |
| **ChrII_A_nidulans_FGSC_A4:2741917-2741967** | **AN3897** |
| **ChrVII_A_nidulans_FGSC_A4:2269982-2270032** | **AN1923** |
| **ChrVII_A_nidulans_FGSC_A4:998030-998080** | **AN12361** |
| **ChrVII_A_nidulans_FGSC_A4:437877-437927** | **AN8918** |
| **ChrVI_A_nidulans_FGSC_A4:799424-799474** | **AN3412** |
| **ChrVI_A_nidulans_FGSC_A4:799424-799474** | **AN3413** |
| **ChrIII_A_nidulans_FGSC_A4:561155-561205** | **AN4918** |
| **ChrIII_A_nidulans_FGSC_A4:1573225-1573275** | **AN4596** |
| **ChrII_A_nidulans_FGSC_A4:2920378-2920428** | **AN12174** |
| **ChrIV_A_nidulans_FGSC_A4:2125308-2125358** | **AN7607** |
| **ChrVIII_A_nidulans_FGSC_A4:4025863-4025913** | **AN0285** |
| **ChrVIII_A_nidulans_FGSC_A4:4025863-4025913** | **AN0286** |
| **ChrVIII_A_nidulans_FGSC_A4:4388642-4388692** | **AN0169** |
| **ChrVII_A_nidulans_FGSC_A4:2913835-2913885** | **AN2116** |
| **ChrV_A_nidulans_FGSC_A4:2532647-2532697** | **AN5518** |
| **ChrV_A_nidulans_FGSC_A4:2532647-2532697** | **AN5519** |
| **ChrVIII_A_nidulans_FGSC_A4:1287399-1287449** | **AN1179** |
| **ChrIV_A_nidulans_FGSC_A4:2142075-2142125** | **AN7613** |
| **ChrVIII_A_nidulans_FGSC_A4:218384-218434** | **AN9299** |
| **ChrVII_A_nidulans_FGSC_A4:317314-317364** | **AN8953** |
| **ChrVII_A_nidulans_FGSC_A4:2725070-2725120** | **AN2060** |
| **ChrVII_A_nidulans_FGSC_A4:2725070-2725120** | **AN11861** |
| **ChrIV_A_nidulans_FGSC_A4:2080607-2080657** | **AN7594** |
| **ChrVI_A_nidulans_FGSC_A4:2072768-2072818** | **AN3024** |
| **ChrVI_A_nidulans_FGSC_A4:2072768-2072818** | **AN3026** |
| **ChrV_A_nidulans_FGSC_A4:1190051-1190101** | **AN5111** |
| **ChrIII_A_nidulans_FGSC_A4:1336893-1336943** | **AN11974** |
| **ChrIV_A_nidulans_FGSC_A4:1420172-1420222** | **AN12059** |
| **ChrIV_A_nidulans_FGSC_A4:1967515-1967565** | **AN7565** |
| **ChrIV_A_nidulans_FGSC_A4:1967515-1967565** | **AN7564** |
| **ChrVII_A_nidulans_FGSC_A4:1556619-1556669** | **AN1666** |
| **ChrIV_A_nidulans_FGSC_A4:2546209-2546259** | **AN7738** |
| **ChrII_A_nidulans_FGSC_A4:3927471-3927521** | **AN10420** |
| **ChrII_A_nidulans_FGSC_A4:3927471-3927521** | **AN3515** |
| **ChrVI_A_nidulans_FGSC_A4:1978400-1978450** | **AN3058** |
| **ChrI_A_nidulans_FGSC_A4:2284582-2284632** | **AN6493** |
| **ChrVII_A_nidulans_FGSC_A4:4328577-4328627** | **AN2550** |
| **ChrI_A_nidulans_FGSC_A4:830258-830308** | **AN12359** |
| **ChrVII_A_nidulans_FGSC_A4:4386062-4386112** | **AN2571** |
| **ChrVI_A_nidulans_FGSC_A4:2941846-2941896** | **AN2745** |
| **ChrV_A_nidulans_FGSC_A4:395996-396046** | **AN8411** |
| **ChrV_A_nidulans_FGSC_A4:395996-396046** | **AN8412** |
| **ChrIII_A_nidulans_FGSC_A4:2249138-2249188** | **AN4369** |
| **ChrV_A_nidulans_FGSC_A4:885175-885225** | **AN8565** |
| **ChrV_A_nidulans_FGSC_A4:885175-885225** | **AN8566** |
| **ChrIII_A_nidulans_FGSC_A4:202476-202526** | **AN11455** |
| **ChrII_A_nidulans_FGSC_A4:866539-866589** | **AN8136** |
| **ChrII_A_nidulans_FGSC_A4:1253159-1253209** | **AN11062** |
| **ChrIV_A_nidulans_FGSC_A4:2469710-2469760** | **AN11006** |
| **ChrV_A_nidulans_FGSC_A4:2086941-2086991** | **AN5652** |
| **ChrVI_A_nidulans_FGSC_A4:2437197-2437247** | **AN2907** |
| **ChrIII_A_nidulans_FGSC_A4:1854536-1854586** | **AN4499** |
| **ChrVII_A_nidulans_FGSC_A4:1597603-1597653** | **AN1681** |
| **ChrVII_A_nidulans_FGSC_A4:2135382-2135432** | **AN10247** |
| **ChrVII_A_nidulans_FGSC_A4:3412822-3412872** | **AN2276** |
| **ChrVI_A_nidulans_FGSC_A4:2016970-2017020** | **AN3045** |
| **ChrV_A_nidulans_FGSC_A4:3240510-3240560** | **AN10658** |
| **ChrIII_A_nidulans_FGSC_A4:1763639-1763689** | **AN4524** |
| **ChrIII_A_nidulans_FGSC_A4:2070797-2070847** | **AN4430** |
| **ChrIII_A_nidulans_FGSC_A4:2070797-2070847** | **AN4431** |
| **ChrVIII_A_nidulans_FGSC_A4:2252006-2252056** | **AN0858** |
| **ChrI_A_nidulans_FGSC_A4:2825944-2825994** | **AN6680** |
| **ChrII_A_nidulans_FGSC_A4:3309376-3309426** | **AN11411** |
| **ChrV_A_nidulans_FGSC_A4:2193619-2193669** | **AN5619** |
| **ChrV_A_nidulans_FGSC_A4:2395793-2395843** | **AN5565** |
| **ChrVIII_A_nidulans_FGSC_A4:2999459-2999509** | **AN0608** |
| **ChrVIII_A_nidulans_FGSC_A4:225149-225199** | **AN11215** |
| **ChrIII_A_nidulans_FGSC_A4:29145-29195** | **AN5086** |
| **ChrVIII_A_nidulans_FGSC_A4:640617-640667** | **AN1397** |
| **ChrVIII_A_nidulans_FGSC_A4:3447950-3448000** | **AN0473** |
| **ChrIV_A_nidulans_FGSC_A4:2192569-2192619** | **AN7629** |
| **ChrIV_A_nidulans_FGSC_A4:2388330-2388380** | **AN7690** |
| **ChrVII_A_nidulans_FGSC_A4:903632-903682** | **AN1477** |
| **ChrII_A_nidulans_FGSC_A4:3235197-3235247** | **AN3734** |
| **ChrVII_A_nidulans_FGSC_A4:175923-175973** | **AN9000** |
| **ChrVI_A_nidulans_FGSC_A4:2747722-2747772** | **AN2810** |
| **ChrVI_A_nidulans_FGSC_A4:2623980-2624030** | **AN2844** |
| **ChrV_A_nidulans_FGSC_A4:204472-204522** | **AN8351** |
| **ChrV_A_nidulans_FGSC_A4:204472-204522** | **AN8352** |
| **ChrV_A_nidulans_FGSC_A4:1822004-1822054** | **AN5745** |
| **ChrIII_A_nidulans_FGSC_A4:1602659-1602709** | **AN4586** |
| **ChrIV_A_nidulans_FGSC_A4:655614-655664** | **AN10908** |
| **ChrVII_A_nidulans_FGSC_A4:1611815-1611865** | **AN1685** |
| **ChrVII_A_nidulans_FGSC_A4:3090472-3090522** | **AN2169** |
| **ChrV_A_nidulans_FGSC_A4:1581943-1581993** | **AN5234** |
| **ChrV_A_nidulans_FGSC_A4:1581943-1581993** | **AN5235** |
| **ChrV_A_nidulans_FGSC_A4:166875-166925** | **AN8338** |
| **ChrV_A_nidulans_FGSC_A4:3135208-3135258** | **AN5328** |
| **ChrV_A_nidulans_FGSC_A4:3135208-3135258** | **AN5327** |
| **ChrVIII_A_nidulans_FGSC_A4:1950339-1950389** | **AN11894** |
| **ChrVIII_A_nidulans_FGSC_A4:4790923-4790973** | **AN10002** |
| **ChrI_A_nidulans_FGSC_A4:3623711-3623761** | **AN6933** |
| **ChrVIII_A_nidulans_FGSC_A4:3347399-3347449** | **AN0505** |
| **ChrVI_A_nidulans_FGSC_A4:845072-845122** | **AN3396** |
| **ChrIV_A_nidulans_FGSC_A4:667771-667821** | **AN7212** |
| **ChrVI_A_nidulans_FGSC_A4:2140206-2140256** | **AN2999** |
| **ChrII_A_nidulans_FGSC_A4:2019824-2019874** | **AN4122** |
| **ChrV_A_nidulans_FGSC_A4:153118-153168** | **AN8332** |
| **ChrVII_A_nidulans_FGSC_A4:3323795-3323845** | **AN2248** |
| **ChrII_A_nidulans_FGSC_A4:553654-553704** | **AN8032** |
| **ChrII_A_nidulans_FGSC_A4:553654-553704** | **AN11045** |
| **ChrIII_A_nidulans_FGSC_A4:2124452-2124502** | **AN4412** |
| **ChrIII_A_nidulans_FGSC_A4:2124452-2124502** | **AN10551** |
| **ChrVI_A_nidulans_FGSC_A4:2000847-2000897** | **AN11702** |
| **ChrIV_A_nidulans_FGSC_A4:2570000-2570050** | **AN7745** |
| **ChrIV_A_nidulans_FGSC_A4:2570000-2570050** | **AN7746** |
| **ChrVI_A_nidulans_FGSC_A4:2871015-2871065** | **AN2766** |
| **ChrV_A_nidulans_FGSC_A4:1286928-1286978** | **AN5146** |
| **ChrV_A_nidulans_FGSC_A4:1286928-1286978** | **AN5145** |
| **ChrIII_A_nidulans_FGSC_A4:326416-326466** | **AN10622** |
| **ChrII_A_nidulans_FGSC_A4:1718633-1718683** | **AN4217** |
| **ChrII_A_nidulans_FGSC_A4:3700276-3700326** | **AN3581** |
| **ChrI_A_nidulans_FGSC_A4:1567543-1567593** | **AN5992** |
| **ChrI_A_nidulans_FGSC_A4:1567543-1567593** | **AN5991** |
| **ChrIII_A_nidulans_FGSC_A4:807809-807859** | **AN10605** |
| **ChrIV_A_nidulans_FGSC_A4:2166934-2166984** | **AN7621** |
| **ChrVIII_A_nidulans_FGSC_A4:511741-511791** | **AN9396** |
| **ChrI_A_nidulans_FGSC_A4:2356614-2356664** | **AN6521** |
| **ChrIV_A_nidulans_FGSC_A4:1319193-1319243** | **AN7013** |
| **ChrIV_A_nidulans_FGSC_A4:1319193-1319243** | **AN7014** |
| **ChrII_A_nidulans_FGSC_A4:166441-166491** | **AN7895** |
| **ChrI_A_nidulans_FGSC_A4:2354314-2354364** | **AN6520** |
| **ChrVII_A_nidulans_FGSC_A4:361862-361912** | **AN8943** |
| **ChrVII_A_nidulans_FGSC_A4:4457077-4457127** | **AN2589** |
| **ChrI_A_nidulans_FGSC_A4:2068353-2068403** | **AN10740** |
| **ChrII_A_nidulans_FGSC_A4:1260978-1261028** | **AN8244** |
| **ChrII_A_nidulans_FGSC_A4:1260978-1261028** | **AN11069** |
| **ChrII_A_nidulans_FGSC_A4:3242122-3242172** | **AN3732** |
| **ChrVII_A_nidulans_FGSC_A4:3772056-3772106** | **AN2390** |
| **ChrIII_A_nidulans_FGSC_A4:2771004-2771054** | **AN8800** |
| **ChrIV_A_nidulans_FGSC_A4:2423931-2423981** | **AN7701** |
| **ChrI_A_nidulans_FGSC_A4:3504523-3504573** | **AN6896** |
| **ChrVII_A_nidulans_FGSC_A4:1968361-1968411** | **AN1813** |
| **ChrVI_A_nidulans_FGSC_A4:449230-449280** | **AN9095** |
| **ChrV_A_nidulans_FGSC_A4:2720167-2720217** | **AN5465** |
| **ChrVII_A_nidulans_FGSC_A4:3973612-3973662** | **AN12233** |
| **ChrVI_A_nidulans_FGSC_A4:1877115-1877165** | **AN3089** |
| **ChrVII_A_nidulans_FGSC_A4:3269513-3269563** | **AN2229** |
| **ChrVII_A_nidulans_FGSC_A4:2225718-2225768** | **AN1909** |
| **ChrIII_A_nidulans_FGSC_A4:1969170-1969220** | **AN4465** |
| **ChrVIII_A_nidulans_FGSC_A4:4602787-4602837** | **AN0104** |
| **ChrVIII_A_nidulans_FGSC_A4:4602787-4602837** | **AN0103** |
| **ChrVII_A_nidulans_FGSC_A4:1738614-1738664** | **AN1730** |
| **ChrV_A_nidulans_FGSC_A4:2501475-2501525** | **AN5528** |
| **ChrVII_A_nidulans_FGSC_A4:3480546-3480596** | **AN2297** |
| **ChrVII_A_nidulans_FGSC_A4:3480546-3480596** | **AN2298** |
| **ChrI_A_nidulans_FGSC_A4:1859385-1859435** | **AN5900** |
| **ChrVIII_A_nidulans_FGSC_A4:1187449-1187499** | **AN1210** |
| **ChrVIII_A_nidulans_FGSC_A4:1187449-1187499** | **AN1211** |
| **ChrIII_A_nidulans_FGSC_A4:1775758-1775808** | **AN4522** |
| **ChrIV_A_nidulans_FGSC_A4:288035-288085** | **AN7330** |
| **ChrVII_A_nidulans_FGSC_A4:2257165-2257215** | **AN1918** |
| **ChrIII_A_nidulans_FGSC_A4:1689096-1689146** | **AN4549** |
| **ChrIII_A_nidulans_FGSC_A4:1689096-1689146** | **AN4548** |
| **ChrVI_A_nidulans_FGSC_A4:1425566-1425616** | **AN3220** |
| **ChrIII_A_nidulans_FGSC_A4:2147380-2147430** | **AN4403** |
| **ChrVIII_A_nidulans_FGSC_A4:1807343-1807393** | **AN10140** |
| **ChrI_A_nidulans_FGSC_A4:1209201-1209251** | **AN6118** |
| **ChrI_A_nidulans_FGSC_A4:2882786-2882836** | **AN6696** |
| **ChrV_A_nidulans_FGSC_A4:1699644-1699694** | **AN5788** |
| **ChrIII_A_nidulans_FGSC_A4:961683-961733** | **AN4796** |
| **ChrVII_A_nidulans_FGSC_A4:3313424-3313474** | **AN2247** |
| **ChrVII_A_nidulans_FGSC_A4:3313424-3313474** | **AN2246** |
| **ChrII_A_nidulans_FGSC_A4:3112160-3112210** | **AN10467** |
| **ChrI_A_nidulans_FGSC_A4:1018016-1018066** | **AN6173** |
| **ChrIII_A_nidulans_FGSC_A4:1814938-1814988** | **AN4513** |
| **ChrVI_A_nidulans_FGSC_A4:24274-24324** | **AN11201** |
| **ChrVI_A_nidulans_FGSC_A4:477257-477307** | **AN9087** |
| **ChrVI_A_nidulans_FGSC_A4:870966-871016** | **AN3388** |
| **ChrII_A_nidulans_FGSC_A4:2218479-2218529** | **AN4056** |
| **ChrVIII_A_nidulans_FGSC_A4:4341144-4341194** | **AN0187** |
| **ChrIII_A_nidulans_FGSC_A4:1117215-1117265** | **AN4745** |
| **ChrII_A_nidulans_FGSC_A4:64152-64202** | **AN7868** |
| **ChrVII_A_nidulans_FGSC_A4:1389743-1389793** | **AN11882** |
| **ChrII_A_nidulans_FGSC_A4:544728-544778** | **AN8030** |
| **ChrII_A_nidulans_FGSC_A4:3579089-3579139** | **AN10432** |
| **ChrIV_A_nidulans_FGSC_A4:2104412-2104462** | **AN7600** |
| **ChrIII_A_nidulans_FGSC_A4:2915956-2916006** | **AN8754** |
| **ChrVIII_A_nidulans_FGSC_A4:1033280-1033330** | **AN1256** |
| **ChrVIII_A_nidulans_FGSC_A4:757592-757642** | **AN1355** |
| **ChrVIII_A_nidulans_FGSC_A4:2310968-2311018** | **AN0840** |
| **ChrIII_A_nidulans_FGSC_A4:401779-401829** | **AN4975** |
| **ChrIII_A_nidulans_FGSC_A4:401779-401829** | **AN4974** |
| **ChrIII_A_nidulans_FGSC_A4:1639134-1639184** | **AN4567** |
| **ChrVIII_A_nidulans_FGSC_A4:363209-363259** | **AN9344** |
| **ChrVII_A_nidulans_FGSC_A4:1935330-1935380** | **AN1802** |
| **ChrVII_A_nidulans_FGSC_A4:389159-389209** | **AN8933** |
| **ChrV_A_nidulans_FGSC_A4:3264354-3264404** | **AN11948** |
| **ChrIII_A_nidulans_FGSC_A4:1383246-1383296** | **AN4659** |
| **ChrIV_A_nidulans_FGSC_A4:333032-333082** | **AN7313** |
| **ChrIII_A_nidulans_FGSC_A4:1613443-1613493** | **AN4580** |
| **ChrV_A_nidulans_FGSC_A4:1955904-1955954** | **AN11788** |
| **ChrII_A_nidulans_FGSC_A4:1298368-1298418** | **AN12205** |
| **ChrII_A_nidulans_FGSC_A4:3049814-3049864** | **AN3791** |
| **ChrIV_A_nidulans_FGSC_A4:172248-172298** | **AN7365** |
| **ChrIV_A_nidulans_FGSC_A4:172248-172298** | **AN7366** |
| **ChrVIII_A_nidulans_FGSC_A4:1988482-1988532** | **AN10136** |
| **ChrVIII_A_nidulans_FGSC_A4:2100876-2100926** | **AN0909** |
| **ChrIII_A_nidulans_FGSC_A4:496565-496615** | **AN4939** |
| **ChrIV_A_nidulans_FGSC_A4:725921-725971** | **AN7193** |
| **ChrVII_A_nidulans_FGSC_A4:2579276-2579326** | **AN2018** |
| **ChrVI_A_nidulans_FGSC_A4:2864723-2864773** | **AN10340** |
| **ChrIV_A_nidulans_FGSC_A4:1216633-1216683** | **AN7042** |
| **ChrIV_A_nidulans_FGSC_A4:1216633-1216683** | **AN11543** |
| **ChrIV_A_nidulans_FGSC_A4:1216633-1216683** | **AN7043** |
| **ChrVII_A_nidulans_FGSC_A4:1979338-1979388** | **AN1815** |
| **ChrV_A_nidulans_FGSC_A4:834830-834880** | **AN8549** |
| **ChrV_A_nidulans_FGSC_A4:1865529-1865579** | **AN5729** |
| **ChrVI_A_nidulans_FGSC_A4:3293193-3293243** | **AN2639** |
| **ChrI_A_nidulans_FGSC_A4:314381-314431** | **AN6395** |
| **ChrII_A_nidulans_FGSC_A4:276279-276329** | **AN7938** |
| **ChrV_A_nidulans_FGSC_A4:2609874-2609924** | **AN5497** |
| **ChrIII_A_nidulans_FGSC_A4:971456-971506** | **AN4794** |
| **ChrII_A_nidulans_FGSC_A4:3505713-3505763** | **AN3645** |
| **ChrII_A_nidulans_FGSC_A4:3505713-3505763** | **AN3643** |
| **ChrVIII_A_nidulans_FGSC_A4:4513471-4513521** | **AN0127** |
| **ChrI_A_nidulans_FGSC_A4:3100314-3100364** | **AN6765** |
| **ChrI_A_nidulans_FGSC_A4:3100314-3100364** | **AN6766** |
| **ChrVIII_A_nidulans_FGSC_A4:409463-409513** | **AN11828** |
| **ChrII_A_nidulans_FGSC_A4:1601472-1601522** | **AN4257** |
| **ChrI_A_nidulans_FGSC_A4:1222970-1223020** | **AN6113** |
| **ChrVI_A_nidulans_FGSC_A4:885715-885765** | **AN3386** |
| **ChrVI_A_nidulans_FGSC_A4:939065-939115** | **AN3369** |
| **ChrV_A_nidulans_FGSC_A4:410921-410971** | **AN8414** |
| **ChrV_A_nidulans_FGSC_A4:410921-410971** | **AN8413** |
| **ChrIII_A_nidulans_FGSC_A4:1591651-1591701** | **AN4590** |
| **ChrI_A_nidulans_FGSC_A4:3653211-3653261** | **AN6942** |
| **ChrVI_A_nidulans_FGSC_A4:3159289-3159339** | **AN2682** |
| **ChrV_A_nidulans_FGSC_A4:297359-297409** | **AN8379** |
| **ChrI_A_nidulans_FGSC_A4:884597-884647** | **AN6218** |
| **ChrVII_A_nidulans_FGSC_A4:2310329-2310379** | **AN1936** |
| **ChrVIII_A_nidulans_FGSC_A4:4745924-4745974** | **AN0052** |
| **ChrVIII_A_nidulans_FGSC_A4:4745924-4745974** | **AN0053** |
| **ChrIV_A_nidulans_FGSC_A4:314343-314393** | **AN7319** |
| **ChrI_A_nidulans_FGSC_A4:2162880-2162930** | **AN5817** |
| **ChrVI_A_nidulans_FGSC_A4:3145650-3145700** | **AN2687** |
| **ChrVI_A_nidulans_FGSC_A4:384213-384263** | **AN9118** |
| **ChrII_A_nidulans_FGSC_A4:507113-507163** | **AN8016** |
| **ChrII_A_nidulans_FGSC_A4:507113-507163** | **AN8015** |
| **ChrVIII_A_nidulans_FGSC_A4:3968120-3968170** | **AN0303** |
| **ChrV_A_nidulans_FGSC_A4:2338058-2338108** | **AN5579** |
| **ChrV_A_nidulans_FGSC_A4:2338058-2338108** | **AN5580** |
| **ChrVIII_A_nidulans_FGSC_A4:4731487-4731537** | **AN10014** |
| **ChrVI_A_nidulans_FGSC_A4:797193-797243** | **AN3414** |
| **ChrII_A_nidulans_FGSC_A4:329757-329807** | **AN7960** |
| **ChrVII_A_nidulans_FGSC_A4:2446757-2446807** | **AN1978** |
| **ChrII_A_nidulans_FGSC_A4:3538339-3538389** | **AN3631** |
| **ChrV_A_nidulans_FGSC_A4:1950485-1950535** | **AN5698** |
| **ChrII_A_nidulans_FGSC_A4:2859686-2859736** | **AN10478** |
| **ChrVIII_A_nidulans_FGSC_A4:3152063-3152113** | **AN0566** |
| **ChrVII_A_nidulans_FGSC_A4:1203796-1203846** | **AN10217** |
| **ChrVIII_A_nidulans_FGSC_A4:4449695-4449745** | **AN0149** |
| **ChrVIII_A_nidulans_FGSC_A4:4449695-4449745** | **AN0150** |
| **ChrIII_A_nidulans_FGSC_A4:3417253-3417303** | **AN8595** |
| **ChrIII_A_nidulans_FGSC_A4:3417253-3417303** | **AN11090** |
| **ChrII_A_nidulans_FGSC_A4:454654-454704** | **AN8000** |
| **ChrVII_A_nidulans_FGSC_A4:3835910-3835960** | **AN10301** |
| **ChrV_A_nidulans_FGSC_A4:3238494-3238544** | **AN10668** |
| **ChrVIII_A_nidulans_FGSC_A4:1160352-1160402** | **AN1219** |
| **ChrIV_A_nidulans_FGSC_A4:339584-339634** | **AN10924** |
| **ChrVIII_A_nidulans_FGSC_A4:3267783-3267833** | **AN0531** |
| **ChrVIII_A_nidulans_FGSC_A4:3267783-3267833** | **AN0532** |
| **ChrV_A_nidulans_FGSC_A4:2869926-2869976** | **AN11810** |
| **ChrI_A_nidulans_FGSC_A4:265788-265838** | **AN6413** |
| **ChrVI_A_nidulans_FGSC_A4:1807958-1808008** | **AN10375** |
| **ChrVI_A_nidulans_FGSC_A4:2176318-2176368** | **AN2983** |
| **ChrVI_A_nidulans_FGSC_A4:2929201-2929251** | **AN2749** |
| **ChrV_A_nidulans_FGSC_A4:1398122-1398172** | **AN11467** |
| **ChrVII_A_nidulans_FGSC_A4:1961499-1961549** | **AN1810** |
| **ChrI_A_nidulans_FGSC_A4:1608659-1608709** | **AN5979** |
| **ChrI_A_nidulans_FGSC_A4:3180314-3180364** | **AN6790** |
| **ChrVII_A_nidulans_FGSC_A4:809486-809536** | **AN1447** |
| **ChrVII_A_nidulans_FGSC_A4:809486-809536** | **AN1448** |
| **ChrV_A_nidulans_FGSC_A4:1945766-1945816** | **AN5701** |
| **ChrII_A_nidulans_FGSC_A4:2082527-2082577** | **AN4101** |
| **ChrIV_A_nidulans_FGSC_A4:2649589-2649639** | **AN7771** |
| **ChrVII_A_nidulans_FGSC_A4:239323-239373** | **AN8976** |
| **ChrVII_A_nidulans_FGSC_A4:2014069-2014119** | **AN1829** |
| **ChrII_A_nidulans_FGSC_A4:2906023-2906073** | **AN3837** |
| **ChrII_A_nidulans_FGSC_A4:2906023-2906073** | **AN3836** |
| **ChrIV_A_nidulans_FGSC_A4:817052-817102** | **AN12147** |
| **ChrIII_A_nidulans_FGSC_A4:338967-339017** | **AN4995** |
| **ChrIII_A_nidulans_FGSC_A4:1662876-1662926** | **AN4559** |
| **ChrIII_A_nidulans_FGSC_A4:1662876-1662926** | **AN4561** |
| **ChrII_A_nidulans_FGSC_A4:12722-12772** | **AN7853** |
| **ChrII_A_nidulans_FGSC_A4:579629-579679** | **AN8041** |
| **ChrII_A_nidulans_FGSC_A4:3404011-3404061** | **AN11724** |
| **ChrI_A_nidulans_FGSC_A4:1361408-1361458** | **AN6069** |
| **ChrI_A_nidulans_FGSC_A4:1361408-1361458** | **AN6068** |
| **ChrVIII_A_nidulans_FGSC_A4:2853221-2853271** | **AN0663** |
| **ChrVI_A_nidulans_FGSC_A4:2357533-2357583** | **AN10349** |
| **ChrV_A_nidulans_FGSC_A4:3314908-3314958** | **AN5270** |
| **ChrVIII_A_nidulans_FGSC_A4:2645301-2645351** | **AN10118** |
| **ChrVI_A_nidulans_FGSC_A4:1882313-1882363** | **AN3088** |
| **ChrIV_A_nidulans_FGSC_A4:2167729-2167779** | **AN7622** |
| **ChrVI_A_nidulans_FGSC_A4:2978646-2978696** | **AN2736** |
| **ChrIII_A_nidulans_FGSC_A4:2705353-2705403** | **AN12435** |
| **ChrVII_A_nidulans_FGSC_A4:3238415-3238465** | **AN2220** |
| **ChrVI_A_nidulans_FGSC_A4:670299-670349** | **AN3452** |
| **ChrII_A_nidulans_FGSC_A4:3362607-3362657** | **AN3690** |
| **ChrVIII_A_nidulans_FGSC_A4:2121899-2121949** | **AN0902** |
| **ChrV_A_nidulans_FGSC_A4:2157850-2157900** | **AN5630** |
| **ChrI_A_nidulans_FGSC_A4:1223714-1223764** | **AN6113** |
| **ChrVII_A_nidulans_FGSC_A4:2510640-2510690** | **AN1998** |
| **ChrVII_A_nidulans_FGSC_A4:4488072-4488122** | **AN2601** |
| **ChrVIII_A_nidulans_FGSC_A4:1110617-1110667** | **AN1237** |
| **ChrVIII_A_nidulans_FGSC_A4:3054692-3054742** | **AN0593** |
| **ChrVI_A_nidulans_FGSC_A4:2479805-2479855** | **AN11687** |
| **ChrII_A_nidulans_FGSC_A4:2890060-2890110** | **AN3844** |
| **ChrVIII_A_nidulans_FGSC_A4:2704769-2704819** | **AN12368** |
| **ChrIV_A_nidulans_FGSC_A4:1243312-1243362** | **AN7034** |
| **ChrVII_A_nidulans_FGSC_A4:3790246-3790296** | **AN2394** |
| **ChrIII_A_nidulans_FGSC_A4:636024-636074** | **AN4895** |
| **ChrVI_A_nidulans_FGSC_A4:2722834-2722884** | **AN12379** |
| **ChrVII_A_nidulans_FGSC_A4:769553-769603** | **AN1434** |
| **ChrIII_A_nidulans_FGSC_A4:52026-52076** | **AN12281** |
| **ChrI_A_nidulans_FGSC_A4:3512577-3512627** | **AN6898** |
| **ChrI_A_nidulans_FGSC_A4:3512577-3512627** | **AN6897** |
| **ChrV_A_nidulans_FGSC_A4:1403818-1403868** | **AN5176** |
| **ChrV_A_nidulans_FGSC_A4:2515579-2515629** | **AN5526** |
| **ChrV_A_nidulans_FGSC_A4:2515579-2515629** | **AN5525** |
| **ChrV_A_nidulans_FGSC_A4:2620531-2620581** | **AN5492** |
| **ChrVII_A_nidulans_FGSC_A4:1150186-1150236** | **AN12136** |
| **ChrIII_A_nidulans_FGSC_A4:2218385-2218435** | **AN4377** |
| **ChrVIII_A_nidulans_FGSC_A4:1777548-1777598** | **AN1022** |
| **ChrV_A_nidulans_FGSC_A4:757131-757181** | **AN8525** |
| **ChrIV_A_nidulans_FGSC_A4:1594186-1594236** | **AN7453** |
| **ChrI_A_nidulans_FGSC_A4:3041814-3041864** | **AN6742** |
| **ChrIII_A_nidulans_FGSC_A4:1735869-1735919** | **AN4534** |
| **ChrIII_A_nidulans_FGSC_A4:1735869-1735919** | **AN10564** |
| **ChrII_A_nidulans_FGSC_A4:639114-639164** | **AN8064** |
| **ChrII_A_nidulans_FGSC_A4:639114-639164** | **AN8065** |
| **ChrVIII_A_nidulans_FGSC_A4:3437812-3437862** | **AN11738** |
| **ChrV_A_nidulans_FGSC_A4:2614473-2614523** | **AN5495** |
| **ChrV_A_nidulans_FGSC_A4:2614473-2614523** | **AN5494** |
| **ChrII_A_nidulans_FGSC_A4:1007200-1007250** | **AN8173** |
| **ChrVIII_A_nidulans_FGSC_A4:2792508-2792558** | **AN0686** |
| **ChrVII_A_nidulans_FGSC_A4:2355682-2355732** | **AN11801** |
| **ChrVI_A_nidulans_FGSC_A4:2877570-2877620** | **AN2764** |
| **ChrVI_A_nidulans_FGSC_A4:2877570-2877620** | **AN2763** |
| **ChrVIII_A_nidulans_FGSC_A4:1635970-1636020** | **AN1062** |
| **ChrIII_A_nidulans_FGSC_A4:1182870-1182920** | **AN4721** |
| **ChrV_A_nidulans_FGSC_A4:429894-429944** | **AN8419** |
| **ChrII_A_nidulans_FGSC_A4:1617366-1617416** | **AN10535** |
| **ChrI_A_nidulans_FGSC_A4:2570620-2570670** | **AN6593** |
| **ChrIII_A_nidulans_FGSC_A4:2797386-2797436** | **AN8789** |
| **ChrIII_A_nidulans_FGSC_A4:2893263-2893313** | **AN8763** |
| **ChrI_A_nidulans_FGSC_A4:2076225-2076275** | **AN5839** |
| **ChrI_A_nidulans_FGSC_A4:1496116-1496166** | **AN6016** |
| **ChrVIII_A_nidulans_FGSC_A4:2216857-2216907** | **AN0870** |
| **ChrVIII_A_nidulans_FGSC_A4:2982664-2982714** | **AN0614** |
| **ChrVIII_A_nidulans_FGSC_A4:2982664-2982714** | **AN0613** |
| **ChrVII_A_nidulans_FGSC_A4:1793098-1793148** | **AN1749** |
| **ChrVII_A_nidulans_FGSC_A4:2664600-2664650** | **AN2037** |
| **ChrVI_A_nidulans_FGSC_A4:2105329-2105379** | **AN3012** |
| **ChrIII_A_nidulans_FGSC_A4:3058724-3058774** | **AN8708** |
| **ChrIII_A_nidulans_FGSC_A4:3058724-3058774** | **AN8707** |
| **ChrII_A_nidulans_FGSC_A4:788845-788895** | **AN8112** |
| **ChrII_A_nidulans_FGSC_A4:788845-788895** | **AN8113** |
| **ChrII_A_nidulans_FGSC_A4:1088585-1088635** | **AN12427** |
| **ChrVIII_A_nidulans_FGSC_A4:202327-202377** | **AN9295** |
| **ChrII_A_nidulans_FGSC_A4:2705976-2706026** | **AN12388** |
| **ChrVI_A_nidulans_FGSC_A4:3216235-3216285** | **AN2665** |
| **ChrVI_A_nidulans_FGSC_A4:3216235-3216285** | **AN2666** |
| **ChrI_A_nidulans_FGSC_A4:1245061-1245111** | **AN6105** |
| **ChrIV_A_nidulans_FGSC_A4:2731027-2731077** | **AN7800** |
| **ChrVIII_A_nidulans_FGSC_A4:2938637-2938687** | **AN0632** |
| **ChrIV_A_nidulans_FGSC_A4:1297525-1297575** | **AN12063** |
| **ChrVII_A_nidulans_FGSC_A4:4365191-4365241** | **AN2562** |
| **ChrIII_A_nidulans_FGSC_A4:848438-848488** | **AN4830** |
| **ChrIV_A_nidulans_FGSC_A4:194230-194280** | **AN7358** |
| **ChrVI_A_nidulans_FGSC_A4:385931-385981** | **AN9117** |
| **ChrV_A_nidulans_FGSC_A4:3075486-3075536** | **AN5349** |
| **ChrV_A_nidulans_FGSC_A4:3075486-3075536** | **AN5350** |
| **ChrIV_A_nidulans_FGSC_A4:930389-930439** | **AN7126** |
| **ChrVIII_A_nidulans_FGSC_A4:4620984-4621034** | **AN0098** |
| **ChrV_A_nidulans_FGSC_A4:2480909-2480959** | **AN10683** |
| **ChrVIII_A_nidulans_FGSC_A4:147244-147294** | **AN9274** |
| **ChrVI_A_nidulans_FGSC_A4:3242380-3242430** | **AN2657** |
| **ChrVII_A_nidulans_FGSC_A4:1128922-1128972** | **AN1547** |
| **ChrII_A_nidulans_FGSC_A4:3318265-3318315** | **AN3707** |
| **ChrVII_A_nidulans_FGSC_A4:2204297-2204347** | **AN1902** |
| **ChrV_A_nidulans_FGSC_A4:2351791-2351841** | **AN5577** |
| **ChrV_A_nidulans_FGSC_A4:2351791-2351841** | **AN5578** |
| **ChrIII_A_nidulans_FGSC_A4:3207847-3207897** | **AN12031** |
| **ChrII_A_nidulans_FGSC_A4:330942-330992** | **AN7959** |
| **ChrI_A_nidulans_FGSC_A4:1014134-1014184** | **AN6177** |
| **ChrI_A_nidulans_FGSC_A4:1014134-1014184** | **AN6176** |
| **ChrI_A_nidulans_FGSC_A4:2165218-2165268** | **AN5815** |
| **ChrI_A_nidulans_FGSC_A4:2165218-2165268** | **AN5814** |
| **ChrVIII_A_nidulans_FGSC_A4:402643-402693** | **AN9360** |
| **ChrVIII_A_nidulans_FGSC_A4:2510519-2510569** | **AN10120** |
| **ChrVIII_A_nidulans_FGSC_A4:3896438-3896488** | **AN0324** |
| **ChrVII_A_nidulans_FGSC_A4:563656-563706** | **AN11140** |
| **ChrIV_A_nidulans_FGSC_A4:2305656-2305706** | **AN7662** |
| **ChrVII_A_nidulans_FGSC_A4:405828-405878** | **AN8928** |
| **ChrVII_A_nidulans_FGSC_A4:2739560-2739610** | **AN11863** |
| **ChrIII_A_nidulans_FGSC_A4:2994198-2994248** | **AN8732** |
| **ChrVIII_A_nidulans_FGSC_A4:3396352-3396402** | **AN0489** |
| **ChrV_A_nidulans_FGSC_A4:2566897-2566947** | **AN5510** |
| **ChrV_A_nidulans_FGSC_A4:2566897-2566947** | **AN11815** |
| **ChrVIII_A_nidulans_FGSC_A4:54956-55006** | **AN9247** |
| **ChrVI_A_nidulans_FGSC_A4:1827393-1827443** | **AN3101** |
| **ChrIII_A_nidulans_FGSC_A4:2899248-2899298** | **AN8760** |
| **ChrIII_A_nidulans_FGSC_A4:2899248-2899298** | **AN8759** |
| **ChrII_A_nidulans_FGSC_A4:931178-931228** | **AN8153** |
| **ChrIV_A_nidulans_FGSC_A4:1053475-1053525** | **AN7092** |
| **ChrIV_A_nidulans_FGSC_A4:1053475-1053525** | **AN7091** |
| **ChrVIII_A_nidulans_FGSC_A4:3818743-3818793** | **AN0353** |
| **ChrVII_A_nidulans_FGSC_A4:768339-768389** | **AN12056** |
| **ChrVII_A_nidulans_FGSC_A4:932409-932459** | **AN10195** |
| **ChrVII_A_nidulans_FGSC_A4:1006176-1006226** | **AN1506** |
| **ChrVI_A_nidulans_FGSC_A4:655588-655638** | **AN3459** |
| **ChrVI_A_nidulans_FGSC_A4:2200097-2200147** | **AN2975** |
| **ChrIII_A_nidulans_FGSC_A4:775238-775288** | **AN4855** |
| **ChrIII_A_nidulans_FGSC_A4:2031419-2031469** | **AN4443** |
| **ChrVI_A_nidulans_FGSC_A4:2969533-2969583** | **AN2739** |
| **ChrI_A_nidulans_FGSC_A4:1253037-1253087** | **AN6103** |
| **ChrI_A_nidulans_FGSC_A4:1275515-1275565** | **AN6095** |
| **ChrI_A_nidulans_FGSC_A4:2628623-2628673** | **AN6615** |
| **ChrI_A_nidulans_FGSC_A4:2976687-2976737** | **AN6721** |
| **ChrI_A_nidulans_FGSC_A4:2976687-2976737** | **AN10844** |
| **ChrVIII_A_nidulans_FGSC_A4:4202903-4202953** | **AN0235** |
| **ChrIII_A_nidulans_FGSC_A4:176037-176087** | **AN10624** |
| **ChrIII_A_nidulans_FGSC_A4:176037-176087** | **AN5044** |
| **ChrIII_A_nidulans_FGSC_A4:2943395-2943445** | **AN11116** |
| **ChrII_A_nidulans_FGSC_A4:905241-905291** | **AN8148** |
| **ChrVII_A_nidulans_FGSC_A4:1625831-1625881** | **AN1689** |
| **ChrV_A_nidulans_FGSC_A4:2799549-2799599** | **AN5444** |
| **ChrII_A_nidulans_FGSC_A4:3401098-3401148** | **AN3677** |
| **ChrVIII_A_nidulans_FGSC_A4:1477673-1477723** | **AN1110** |
| **ChrV_A_nidulans_FGSC_A4:454384-454434** | **AN8426** |
| **ChrI_A_nidulans_FGSC_A4:1215882-1215932** | **AN6115** |
| **ChrVI_A_nidulans_FGSC_A4:1295937-1295987** | **AN3264** |
| **ChrIII_A_nidulans_FGSC_A4:1893031-1893081** | **AN11909** |
| **ChrIII_A_nidulans_FGSC_A4:2615079-2615129** | **AN11133** |
| **ChrII_A_nidulans_FGSC_A4:1640696-1640746** | **AN4241** |
| **ChrII_A_nidulans_FGSC_A4:2598233-2598283** | **AN3938** |
| **ChrII_A_nidulans_FGSC_A4:2598233-2598283** | **AN3937** |
| **ChrI_A_nidulans_FGSC_A4:368503-368553** | **AN6378** |
| **ChrI_A_nidulans_FGSC_A4:375460-375510** | **AN6376** |
| **ChrVIII_A_nidulans_FGSC_A4:114648-114698** | **AN9265** |
| **ChrVIII_A_nidulans_FGSC_A4:114648-114698** | **AN9266** |
| **ChrVIII_A_nidulans_FGSC_A4:934850-934900** | **AN10171** |
| **ChrVIII_A_nidulans_FGSC_A4:934850-934900** | **AN1290** |
| **ChrVIII_A_nidulans_FGSC_A4:3505329-3505379** | **AN0454** |
| **ChrVII_A_nidulans_FGSC_A4:1827248-1827298** | **AN1767** |
| **ChrV_A_nidulans_FGSC_A4:3001625-3001675** | **AN5378** |
| **ChrIII_A_nidulans_FGSC_A4:368375-368425** | **AN4986** |
| **ChrIII_A_nidulans_FGSC_A4:492138-492188** | **AN4940** |
| **ChrI_A_nidulans_FGSC_A4:913112-913162** | **AN6207** |
| **ChrIII_A_nidulans_FGSC_A4:697436-697486** | **AN4878** |
| **ChrII_A_nidulans_FGSC_A4:714526-714576** | **AN8089** |
| **ChrIV_A_nidulans_FGSC_A4:1937651-1937701** | **AN11754** |
| **ChrI_A_nidulans_FGSC_A4:1658375-1658425** | **AN9470** |
| **ChrI_A_nidulans_FGSC_A4:1658375-1658425** | **AN12490** |
| **ChrI_A_nidulans_FGSC_A4:2866489-2866539** | **AN6692** |
| **ChrVIII_A_nidulans_FGSC_A4:3496657-3496707** | **AN0456** |
| **ChrVII_A_nidulans_FGSC_A4:3071401-3071451** | **AN2161** |
| **ChrVI_A_nidulans_FGSC_A4:195233-195283** | **AN11188** |
| **ChrVI_A_nidulans_FGSC_A4:502571-502621** | **AN9078** |
| **ChrV_A_nidulans_FGSC_A4:1939595-1939645** | **AN5703** |
| **ChrVII_A_nidulans_FGSC_A4:3296830-3296880** | **AN2240** |
| **ChrVII_A_nidulans_FGSC_A4:3296830-3296880** | **AN10283** |
| **ChrV_A_nidulans_FGSC_A4:1355742-1355792** | **AN5165** |
| **ChrII_A_nidulans_FGSC_A4:535460-535510** | **AN8026** |
| **ChrIV_A_nidulans_FGSC_A4:865330-865380** | **AN7146** |
| **ChrII_A_nidulans_FGSC_A4:1699954-1700004** | **AN4222** |
| **ChrII_A_nidulans_FGSC_A4:3847067-3847117** | **AN3538** |
| **ChrVIII_A_nidulans_FGSC_A4:3628700-3628750** | **AN0413** |
| **ChrV_A_nidulans_FGSC_A4:721644-721694** | **AN8507** |
| **ChrV_A_nidulans_FGSC_A4:2705707-2705757** | **AN5470** |
| **ChrV_A_nidulans_FGSC_A4:3309054-3309104** | **AN10656** |
| **ChrI_A_nidulans_FGSC_A4:3273543-3273593** | **AN6816** |
| **ChrI_A_nidulans_FGSC_A4:3273543-3273593** | **AN6818** |
| **ChrII_A_nidulans_FGSC_A4:1618286-1618336** | **AN4250** |
| **ChrII_A_nidulans_FGSC_A4:1618286-1618336** | **AN10535** |
| **ChrII_A_nidulans_FGSC_A4:1677612-1677662** | **AN4229** |
| **ChrIV_A_nidulans_FGSC_A4:623781-623831** | **AN10915** |
| **ChrII_A_nidulans_FGSC_A4:1797292-1797342** | **AN10521** |
| **ChrIV_A_nidulans_FGSC_A4:541178-541228** | **AN10918** |
| **ChrVIII_A_nidulans_FGSC_A4:321814-321864** | **AN9336** |
| **ChrVII_A_nidulans_FGSC_A4:2185055-2185105** | **AN1896** |
| **ChrVII_A_nidulans_FGSC_A4:2185055-2185105** | **AN1897** |
| **ChrVII_A_nidulans_FGSC_A4:3125729-3125779** | **AN2180** |
| **ChrVI_A_nidulans_FGSC_A4:2508173-2508223** | **AN2883** |
| **ChrII_A_nidulans_FGSC_A4:2692300-2692350** | **AN3909** |
| **ChrV_A_nidulans_FGSC_A4:3022233-3022283** | **AN5370** |
| **ChrIV_A_nidulans_FGSC_A4:922162-922212** | **AN7128** |
| **ChrI_A_nidulans_FGSC_A4:3086879-3086929** | **AN6762** |
| **ChrI_A_nidulans_FGSC_A4:3086879-3086929** | **AN6761** |
| **ChrVIII_A_nidulans_FGSC_A4:194240-194290** | **AN9293** |
| **ChrVIII_A_nidulans_FGSC_A4:953152-953202** | **AN10170** |
| **ChrVIII_A_nidulans_FGSC_A4:2012497-2012547** | **AN0938** |
| **ChrVIII_A_nidulans_FGSC_A4:2012497-2012547** | **AN0939** |
| **ChrVII_A_nidulans_FGSC_A4:1173393-1173443** | **AN1560** |
| **ChrII_A_nidulans_FGSC_A4:2076468-2076518** | **AN10511** |
| **ChrVII_A_nidulans_FGSC_A4:4382366-4382416** | **AN2569** |
| **ChrI_A_nidulans_FGSC_A4:2377089-2377139** | **AN6526** |
| **ChrII_A_nidulans_FGSC_A4:2615425-2615475** | **AN3931** |
| **ChrVI_A_nidulans_FGSC_A4:2794810-2794860** | **AN2794** |
| **ChrII_A_nidulans_FGSC_A4:1771094-1771144** | **AN10522** |
| **ChrVIII_A_nidulans_FGSC_A4:4500290-4500340** | **AN0131** |
| **ChrVII_A_nidulans_FGSC_A4:3813127-3813177** | **AN10300** |
| **ChrI_A_nidulans_FGSC_A4:1149931-1149981** | **AN10776** |
| **ChrVII_A_nidulans_FGSC_A4:3066273-3066323** | **AN10265** |
| **ChrII_A_nidulans_FGSC_A4:2802170-2802220** | **AN10475** |
| **ChrVI_A_nidulans_FGSC_A4:3152307-3152357** | **AN2684** |
| **ChrIV_A_nidulans_FGSC_A4:2334037-2334087** | **AN7672** |
| **ChrVII_A_nidulans_FGSC_A4:54408-54458** | **AN9042** |
| **ChrV_A_nidulans_FGSC_A4:3140253-3140303** | **AN5326** |
| **ChrIII_A_nidulans_FGSC_A4:31448-31498** | **AN10628** |
| **ChrI_A_nidulans_FGSC_A4:430880-430930** | **AN6354** |
| **ChrVII_A_nidulans_FGSC_A4:1022879-1022929** | **AN1513** |
| **ChrVII_A_nidulans_FGSC_A4:1265289-1265339** | **AN1587** |
| **ChrIII_A_nidulans_FGSC_A4:3399927-3399977** | **AN11099** |
| **ChrIII_A_nidulans_FGSC_A4:3399927-3399977** | **AN8601** |
| **ChrVI_A_nidulans_FGSC_A4:2607592-2607642** | **AN2850** |
| **ChrVI_A_nidulans_FGSC_A4:2607592-2607642** | **AN2851** |
| **ChrIII_A_nidulans_FGSC_A4:1463690-1463740** | **AN10575** |
| **ChrVIII_A_nidulans_FGSC_A4:1174555-1174605** | **AN1216** |
| **ChrVIII_A_nidulans_FGSC_A4:1174555-1174605** | **AN1215** |
| **ChrIII_A_nidulans_FGSC_A4:2930767-2930817** | **AN8749** |
| **ChrII_A_nidulans_FGSC_A4:1283040-1283090** | **AN11070** |
| **ChrIV_A_nidulans_FGSC_A4:1245869-1245919** | **AN7033** |
| **ChrIII_A_nidulans_FGSC_A4:2983952-2984002** | **AN8737** |
| **ChrIII_A_nidulans_FGSC_A4:2983952-2984002** | **AN12244** |
| **ChrIV_A_nidulans_FGSC_A4:197695-197745** | **AN7357** |
| **ChrVIII_A_nidulans_FGSC_A4:1063449-1063499** | **AN1246** |
| **ChrVI_A_nidulans_FGSC_A4:447021-447071** | **AN9096** |
| **ChrV_A_nidulans_FGSC_A4:1532802-1532852** | **AN5219** |
| **ChrV_A_nidulans_FGSC_A4:1532802-1532852** | **AN5220** |
| **ChrIII_A_nidulans_FGSC_A4:739855-739905** | **AN4861** |
| **ChrII_A_nidulans_FGSC_A4:3152778-3152828** | **AN3756** |
| **ChrVI_A_nidulans_FGSC_A4:1521780-1521830** | **AN3193** |
| **ChrIII_A_nidulans_FGSC_A4:1981470-1981520** | **AN4462** |
| **ChrVIII_A_nidulans_FGSC_A4:3676551-3676601** | **AN0401** |
| **ChrVI_A_nidulans_FGSC_A4:3181365-3181415** | **AN2675** |
| **ChrVIII_A_nidulans_FGSC_A4:4796269-4796319** | **AN10008** |
| **ChrIII_A_nidulans_FGSC_A4:3325285-3325335** | **AN8628** |
| **ChrVIII_A_nidulans_FGSC_A4:1971923-1971973** | **AN11891** |
| **ChrIII_A_nidulans_FGSC_A4:1379124-1379174** | **AN4663** |
| **ChrI_A_nidulans_FGSC_A4:2709621-2709671** | **AN6642** |
| **ChrI_A_nidulans_FGSC_A4:2962297-2962347** | **AN6718** |
| **ChrVIII_A_nidulans_FGSC_A4:1297362-1297412** | **AN1174** |
| **ChrIV_A_nidulans_FGSC_A4:220215-220265** | **AN7349** |
| **ChrIII_A_nidulans_FGSC_A4:1492257-1492307** | **AN10579** |
| **ChrIII_A_nidulans_FGSC_A4:2264046-2264096** | **AN4366** |
| **ChrIII_A_nidulans_FGSC_A4:2264046-2264096** | **AN4367** |
| **ChrII_A_nidulans_FGSC_A4:624272-624322** | **AN8056** |
| **ChrIV_A_nidulans_FGSC_A4:1378956-1379006** | **AN10879** |
| **ChrVIII_A_nidulans_FGSC_A4:2019220-2019270** | **AN0935** |
| **ChrVIII_A_nidulans_FGSC_A4:4438096-4438146** | **AN10023** |
| **ChrVIII_A_nidulans_FGSC_A4:4438096-4438146** | **AN10038** |
| **ChrV_A_nidulans_FGSC_A4:1855933-1855983** | **AN5733** |
| **ChrVI_A_nidulans_FGSC_A4:1744022-1744072** | **AN10365** |
| **ChrIII_A_nidulans_FGSC_A4:3026119-3026169** | **AN8721** |
| **ChrI_A_nidulans_FGSC_A4:2832729-2832779** | **AN6682** |
| **ChrVI_A_nidulans_FGSC_A4:1234780-1234830** | **AN3285** |
| **ChrII_A_nidulans_FGSC_A4:2679901-2679951** | **AN10491** |
| **ChrIV_A_nidulans_FGSC_A4:591523-591573** | **AN7234** |
| **ChrI_A_nidulans_FGSC_A4:2464107-2464157** | **AN6561** |
| **ChrVIII_A_nidulans_FGSC_A4:2633550-2633600** | **AN0732** |
| **ChrVIII_A_nidulans_FGSC_A4:3044791-3044841** | **AN0595** |
| **ChrVIII_A_nidulans_FGSC_A4:3455368-3455418** | **AN0470** |
| **ChrVIII_A_nidulans_FGSC_A4:4584455-4584505** | **AN0111** |
| **ChrVII_A_nidulans_FGSC_A4:346208-346258** | **AN8947** |
| **ChrVII_A_nidulans_FGSC_A4:2022989-2023039** | **AN11795** |
| **ChrV_A_nidulans_FGSC_A4:663515-663565** | **AN8489** |
| **ChrIV_A_nidulans_FGSC_A4:2676129-2676179** | **AN7781** |
| **ChrIII_A_nidulans_FGSC_A4:270400-270450** | **AN5017** |
| **ChrII_A_nidulans_FGSC_A4:73328-73378** | **AN11581** |
| **ChrII_A_nidulans_FGSC_A4:1055926-1055976** | **AN11053** |
| **ChrVIII_A_nidulans_FGSC_A4:2389430-2389480** | **AN0817** |
| **ChrVII_A_nidulans_FGSC_A4:3407527-3407577** | **AN2272** |
| **ChrIII_A_nidulans_FGSC_A4:1929415-1929465** | **AN10568** |
| **ChrIII_A_nidulans_FGSC_A4:1929415-1929465** | **AN4475** |
| **ChrVIII_A_nidulans_FGSC_A4:451763-451813** | **AN11216** |
| **ChrVIII_A_nidulans_FGSC_A4:1230289-1230339** | **AN1195** |
| **ChrIV_A_nidulans_FGSC_A4:104831-104881** | **AN7390** |
| **ChrIV_A_nidulans_FGSC_A4:2108677-2108727** | **AN7602** |
| **ChrII_A_nidulans_FGSC_A4:2602016-2602066** | **AN3936** |
| **ChrVIII_A_nidulans_FGSC_A4:1648173-1648223** | **AN1059** |
| **ChrVIII_A_nidulans_FGSC_A4:2299418-2299468** | **AN0843** |
| **ChrVIII_A_nidulans_FGSC_A4:2299418-2299468** | **AN0842** |
| **ChrVI_A_nidulans_FGSC_A4:2216596-2216646** | **AN11693** |
| **ChrVI_A_nidulans_FGSC_A4:2216596-2216646** | **AN11694** |
| **ChrII_A_nidulans_FGSC_A4:230613-230663** | **AN7918** |
| **ChrII_A_nidulans_FGSC_A4:1612074-1612124** | **AN4252** |
| **ChrIV_A_nidulans_FGSC_A4:742862-742912** | **AN7188** |
| **ChrIV_A_nidulans_FGSC_A4:742862-742912** | **AN7187** |
| **ChrIV_A_nidulans_FGSC_A4:2374061-2374111** | **AN11002** |
| **ChrI_A_nidulans_FGSC_A4:29977-30027** | **AN6476** |
| **ChrVIII_A_nidulans_FGSC_A4:4196453-4196503** | **AN10048** |
| **ChrVII_A_nidulans_FGSC_A4:1903491-1903541** | **AN1792** |
| **ChrVII_A_nidulans_FGSC_A4:4283826-4283876** | **AN2543** |
| **ChrVI_A_nidulans_FGSC_A4:1072966-1073016** | **AN3333** |
| **ChrVI_A_nidulans_FGSC_A4:1516802-1516852** | **AN3196** |
| **ChrVI_A_nidulans_FGSC_A4:1516802-1516852** | **AN3195** |
| **ChrVIII_A_nidulans_FGSC_A4:1512391-1512441** | **AN10166** |
| **ChrVIII_A_nidulans_FGSC_A4:1627807-1627857** | **AN1066** |
| **ChrVI_A_nidulans_FGSC_A4:681024-681074** | **AN3448** |
| **ChrVI_A_nidulans_FGSC_A4:681024-681074** | **AN3449** |
| **ChrVI_A_nidulans_FGSC_A4:1464268-1464318** | **AN3210** |
| **ChrII_A_nidulans_FGSC_A4:3217988-3218038** | **AN3741** |
| **ChrII_A_nidulans_FGSC_A4:3217988-3218038** | **AN3740** |
| **ChrIII_A_nidulans_FGSC_A4:3110778-3110828** | **AN11109** |
| **ChrII_A_nidulans_FGSC_A4:174996-175046** | **AN11584** |
| **ChrII_A_nidulans_FGSC_A4:174996-175046** | **AN7897** |
| **ChrVIII_A_nidulans_FGSC_A4:173727-173777** | **AN9283** |
| **ChrV_A_nidulans_FGSC_A4:605066-605116** | **AN8476** |
| **ChrV_A_nidulans_FGSC_A4:605066-605116** | **AN8475** |
| **ChrIV_A_nidulans_FGSC_A4:189249-189299** | **AN7361** |
| **ChrI_A_nidulans_FGSC_A4:1325112-1325162** | **AN6078** |
| **ChrII_A_nidulans_FGSC_A4:466869-466919** | **AN8002** |
| **ChrVII_A_nidulans_FGSC_A4:131280-131330** | **AN9014** |
| **ChrVI_A_nidulans_FGSC_A4:1538562-1538612** | **AN10378** |
| **ChrVI_A_nidulans_FGSC_A4:1538562-1538612** | **AN3188** |
| **ChrIII_A_nidulans_FGSC_A4:3440778-3440828** | **AN8587** |
| **ChrII_A_nidulans_FGSC_A4:1973792-1973842** | **AN12097** |
| **ChrI_A_nidulans_FGSC_A4:2440646-2440696** | **AN6551** |
| **ChrI_A_nidulans_FGSC_A4:3613410-3613460** | **AN6930** |
| **ChrVII_A_nidulans_FGSC_A4:3662868-3662918** | **AN2354** |
| **ChrVII_A_nidulans_FGSC_A4:3721354-3721404** | **AN2373** |
| **ChrV_A_nidulans_FGSC_A4:1222309-1222359** | **AN5123** |
| **ChrVII_A_nidulans_FGSC_A4:2982766-2982816** | **AN2133** |
| **ChrIII_A_nidulans_FGSC_A4:3146741-3146791** | **AN8676** |
| **ChrIII_A_nidulans_FGSC_A4:602547-602597** | **AN10615** |
| **ChrIII_A_nidulans_FGSC_A4:2017791-2017841** | **AN4449** |
| **ChrVIII_A_nidulans_FGSC_A4:2702617-2702667** | **AN0712** |
| **ChrVIII_A_nidulans_FGSC_A4:2702617-2702667** | **AN12369** |
| **ChrVIII_A_nidulans_FGSC_A4:2793527-2793577** | **AN12213** |
| **ChrVII_A_nidulans_FGSC_A4:3926880-3926930** | **AN10307** |
| **ChrVI_A_nidulans_FGSC_A4:58316-58366** | **AN9219** |
| **ChrII_A_nidulans_FGSC_A4:1352239-1352289** | **AN8274** |
| **ChrII_A_nidulans_FGSC_A4:2768988-2769038** | **AN3889** |
| **ChrII_A_nidulans_FGSC_A4:3477722-3477772** | **AN11721** |
| **ChrVI_A_nidulans_FGSC_A4:2633823-2633873** | **AN2840** |
| **ChrIII_A_nidulans_FGSC_A4:1742452-1742502** | **AN4532** |
| **ChrVIII_A_nidulans_FGSC_A4:906427-906477** | **AN10172** |
| **ChrVII_A_nidulans_FGSC_A4:4051358-4051408** | **AN2469** |
| **ChrVII_A_nidulans_FGSC_A4:4051358-4051408** | **AN2470** |
| **ChrVI_A_nidulans_FGSC_A4:2937623-2937673** | **AN2746** |
| **ChrVIII_A_nidulans_FGSC_A4:3597274-3597324** | **AN0424** |
| **ChrVII_A_nidulans_FGSC_A4:3443531-3443581** | **AN2285** |
| **ChrVII_A_nidulans_FGSC_A4:3992583-3992633** | **AN2455** |
| **ChrV_A_nidulans_FGSC_A4:154530-154580** | **AN11073** |
| **ChrV_A_nidulans_FGSC_A4:2755390-2755440** | **AN5455** |
| **ChrI_A_nidulans_FGSC_A4:3126766-3126816** | **AN6775** |
| **ChrVIII_A_nidulans_FGSC_A4:1172598-1172648** | **AN10177** |
| **ChrVII_A_nidulans_FGSC_A4:430745-430795** | **AN8921** |
| **ChrVII_A_nidulans_FGSC_A4:2182069-2182119** | **AN1895** |
| **ChrVIII_A_nidulans_FGSC_A4:3758539-3758589** | **AN10059** |
| **ChrVIII_A_nidulans_FGSC_A4:3758539-3758589** | **AN0372** |
| **ChrII_A_nidulans_FGSC_A4:629844-629894** | **AN8060** |
| **ChrIV_A_nidulans_FGSC_A4:235461-235511** | **AN7346** |
| **ChrVIII_A_nidulans_FGSC_A4:2568908-2568958** | **AN0752** |
| **ChrVIII_A_nidulans_FGSC_A4:2568908-2568958** | **AN0753** |
| **ChrI_A_nidulans_FGSC_A4:20865-20915** | **AN6480** |
| **ChrI_A_nidulans_FGSC_A4:449342-449392** | **AN6348** |
| **ChrI_A_nidulans_FGSC_A4:449342-449392** | **AN6349** |
| **ChrVII_A_nidulans_FGSC_A4:4038938-4038988** | **AN2466** |
| **ChrVIII_A_nidulans_FGSC_A4:3271932-3271982** | **AN0528** |
| **ChrVIII_A_nidulans_FGSC_A4:3271932-3271982** | **AN0529** |
| **ChrV_A_nidulans_FGSC_A4:843130-843180** | **AN8552** |
| **ChrV_A_nidulans_FGSC_A4:843130-843180** | **AN8551** |
| **ChrV_A_nidulans_FGSC_A4:1798461-1798511** | **AN5755** |
| **ChrIII_A_nidulans_FGSC_A4:1087685-1087735** | **AN4757** |
| **ChrIII_A_nidulans_FGSC_A4:1087685-1087735** | **AN10583** |
| **ChrIV_A_nidulans_FGSC_A4:410342-410392** | **AN7287** |
| **ChrI_A_nidulans_FGSC_A4:3252008-3252058** | **AN6808** |
| **ChrVII_A_nidulans_FGSC_A4:4484436-4484486** | **AN2599** |
| **ChrIII_A_nidulans_FGSC_A4:391313-391363** | **AN4979** |
| **ChrV_A_nidulans_FGSC_A4:142252-142302** | **AN8327** |
| **ChrIII_A_nidulans_FGSC_A4:1537355-1537405** | **AN4608** |
| **ChrI_A_nidulans_FGSC_A4:2592796-2592846** | **AN6602** |
| **ChrVII_A_nidulans_FGSC_A4:1898867-1898917** | **AN12309** |
| **ChrVIII_A_nidulans_FGSC_A4:3843004-3843054** | **AN10073** |
| **ChrVII_A_nidulans_FGSC_A4:2404768-2404818** | **AN1964** |
| **ChrVII_A_nidulans_FGSC_A4:2404768-2404818** | **AN1963** |
| **ChrVII_A_nidulans_FGSC_A4:3207696-3207746** | **AN2209** |
| **ChrVI_A_nidulans_FGSC_A4:2811469-2811519** | **AN2786** |
| **ChrVI_A_nidulans_FGSC_A4:2811469-2811519** | **AN11366** |
| **ChrIII_A_nidulans_FGSC_A4:2887457-2887507** | **AN8765** |
| **ChrVII_A_nidulans_FGSC_A4:1746388-1746438** | **AN10233** |
| **ChrIII_A_nidulans_FGSC_A4:885919-885969** | **AN4822** |
| **ChrIII_A_nidulans_FGSC_A4:1046965-1047015** | **AN10593** |
| **ChrIII_A_nidulans_FGSC_A4:1046965-1047015** | **AN4770** |
| **ChrII_A_nidulans_FGSC_A4:2422367-2422417** | **AN3987** |
| **ChrVII_A_nidulans_FGSC_A4:1661078-1661128** | **AN1701** |
| **ChrVI_A_nidulans_FGSC_A4:3051969-3052019** | **AN2716** |
| **ChrI_A_nidulans_FGSC_A4:11520-11570** | **AN10820** |
| **ChrVII_A_nidulans_FGSC_A4:2702019-2702069** | **AN2051** |
| **ChrV_A_nidulans_FGSC_A4:3307080-3307130** | **AN10665** |
| **ChrII_A_nidulans_FGSC_A4:2574037-2574087** | **AN3942** |
| **ChrIV_A_nidulans_FGSC_A4:1364170-1364220** | **AN6997** |
| **ChrI_A_nidulans_FGSC_A4:1158218-1158268** | **AN12441** |
| **ChrI_A_nidulans_FGSC_A4:3534683-3534733** | **AN11250** |
| **ChrI_A_nidulans_FGSC_A4:3639415-3639465** | **AN10865** |
| **ChrVIII_A_nidulans_FGSC_A4:80358-80408** | **AN11647** |
| **ChrVIII_A_nidulans_FGSC_A4:80358-80408** | **AN9257** |
| **ChrVIII_A_nidulans_FGSC_A4:3511858-3511908** | **AN0451** |
| **ChrVIII_A_nidulans_FGSC_A4:4759338-4759388** | **AN10003** |
| **ChrVI_A_nidulans_FGSC_A4:3271364-3271414** | **AN2647** |
| **ChrV_A_nidulans_FGSC_A4:1991369-1991419** | **AN5682** |
| **ChrII_A_nidulans_FGSC_A4:3127677-3127727** | **AN3765** |
| **ChrIV_A_nidulans_FGSC_A4:1091590-1091640** | **AN10887** |
| **ChrVIII_A_nidulans_FGSC_A4:3519243-3519293** | **AN0449** |
| **ChrVI_A_nidulans_FGSC_A4:2396390-2396440** | **AN2920** |
| **ChrVI_A_nidulans_FGSC_A4:2396390-2396440** | **AN2919** |
| **ChrV_A_nidulans_FGSC_A4:1445590-1445640** | **AN5191** |
| **ChrVII_A_nidulans_FGSC_A4:1047884-1047934** | **AN1517** |
| **ChrVI_A_nidulans_FGSC_A4:479574-479624** | **AN9085** |
| **ChrVI_A_nidulans_FGSC_A4:479574-479624** | **AN9086** |
| **ChrIII_A_nidulans_FGSC_A4:744317-744367** | **AN4860** |
| **ChrIII_A_nidulans_FGSC_A4:1085553-1085603** | **AN4758** |
| **ChrI_A_nidulans_FGSC_A4:3453908-3453958** | **AN6879** |
| **ChrIII_A_nidulans_FGSC_A4:2450102-2450152** | **AN12152** |
| **ChrIII_A_nidulans_FGSC_A4:2450102-2450152** | **AN4306** |
| **ChrIII_A_nidulans_FGSC_A4:3464155-3464205** | **AN8580** |
| **ChrIV_A_nidulans_FGSC_A4:1754797-1754847** | **AN7505** |
| **ChrI_A_nidulans_FGSC_A4:1287889-1287939** | **AN6091** |
| **ChrI_A_nidulans_FGSC_A4:1639059-1639109** | **AN5969** |
| **ChrI_A_nidulans_FGSC_A4:2931394-2931444** | **AN6710** |
| **ChrVIII_A_nidulans_FGSC_A4:3987726-3987776** | **AN0295** |
| **ChrV_A_nidulans_FGSC_A4:2254232-2254282** | **AN5604** |
| **ChrVIII_A_nidulans_FGSC_A4:896625-896675** | **AN9516** |
| **ChrVI_A_nidulans_FGSC_A4:464262-464312** | **AN9090** |
| **ChrIV_A_nidulans_FGSC_A4:705017-705067** | **AN7199** |
| **ChrVII_A_nidulans_FGSC_A4:3534324-3534374** | **AN2315** |
| **ChrV_A_nidulans_FGSC_A4:1294620-1294670** | **AN5148** |
| **ChrV_A_nidulans_FGSC_A4:1294620-1294670** | **AN5149** |
| **ChrVII_A_nidulans_FGSC_A4:1836325-1836375** | **AN1770** |
| **ChrVI_A_nidulans_FGSC_A4:3211443-3211493** | **AN2667** |
| **ChrII_A_nidulans_FGSC_A4:1676348-1676398** | **AN4231** |
| **ChrII_A_nidulans_FGSC_A4:1676348-1676398** | **AN4230** |
| **ChrVII_A_nidulans_FGSC_A4:130335-130385** | **AN9013** |
| **ChrVI_A_nidulans_FGSC_A4:1579478-1579528** | **AN3177** |
| **ChrVI_A_nidulans_FGSC_A4:1579478-1579528** | **AN3176** |
| **ChrVII_A_nidulans_FGSC_A4:3134193-3134243** | **AN2183** |
| **ChrI_A_nidulans_FGSC_A4:1970474-1970524** | **AN5866** |
| **ChrVIII_A_nidulans_FGSC_A4:2596483-2596533** | **AN0746** |
| **ChrVIII_A_nidulans_FGSC_A4:3708679-3708729** | **AN0389** |
| **ChrVI_A_nidulans_FGSC_A4:2685655-2685705** | **AN2823** |
| **ChrII_A_nidulans_FGSC_A4:3138349-3138399** | **AN3763** |
| **ChrVII_A_nidulans_FGSC_A4:1772248-1772298** | **AN1744** |
| **ChrIII_A_nidulans_FGSC_A4:155991-156041** | **AN5050** |
| **ChrVII_A_nidulans_FGSC_A4:4202314-4202364** | **AN2515** |
| **ChrV_A_nidulans_FGSC_A4:3231182-3231232** | **AN5295** |
| **ChrV_A_nidulans_FGSC_A4:3231182-3231232** | **AN5296** |
| **ChrI_A_nidulans_FGSC_A4:2716090-2716140** | **AN6643** |
| **ChrVII_A_nidulans_FGSC_A4:2576189-2576239** | **AN2017** |
| **ChrIII_A_nidulans_FGSC_A4:3401883-3401933** | **AN11092** |
| **ChrI_A_nidulans_FGSC_A4:987432-987482** | **AN6191** |
| **ChrIV_A_nidulans_FGSC_A4:1785929-1785979** | **AN10957** |
| **ChrIII_A_nidulans_FGSC_A4:2148130-2148180** | **AN4403** |
| **ChrIII_A_nidulans_FGSC_A4:2148130-2148180** | **AN4404** |
| **ChrVIII_A_nidulans_FGSC_A4:760545-760595** | **AN1354** |
| **ChrI_A_nidulans_FGSC_A4:988505-988555** | **AN6189** |
| **ChrVIII_A_nidulans_FGSC_A4:2504210-2504260** | **AN0775** |
| **ChrVIII_A_nidulans_FGSC_A4:2504210-2504260** | **AN0774** |
| **ChrV_A_nidulans_FGSC_A4:2283717-2283767** | **AN5597** |
| **ChrII_A_nidulans_FGSC_A4:265618-265668** | **AN7935** |
| **ChrII_A_nidulans_FGSC_A4:3322803-3322853** | **AN10438** |
| **ChrIV_A_nidulans_FGSC_A4:2843671-2843721** | **AN11575** |
| **ChrI_A_nidulans_FGSC_A4:1430506-1430556** | **AN6045** |
| **ChrVII_A_nidulans_FGSC_A4:448790-448840** | **AN12413** |
| **ChrVI_A_nidulans_FGSC_A4:1641289-1641339** | **AN3153** |
| **ChrIII_A_nidulans_FGSC_A4:2041621-2041671** | **AN4440** |
| **ChrII_A_nidulans_FGSC_A4:2867415-2867465** | **AN3854** |
| **ChrIV_A_nidulans_FGSC_A4:2309456-2309506** | **AN7663** |
| **ChrVII_A_nidulans_FGSC_A4:3585055-3585105** | **AN2332** |
| **ChrVI_A_nidulans_FGSC_A4:295333-295383** | **AN9148** |
| **ChrIII_A_nidulans_FGSC_A4:631496-631546** | **AN4896** |
| **ChrV_A_nidulans_FGSC_A4:389276-389326** | **AN8409** |
| **ChrV_A_nidulans_FGSC_A4:389276-389326** | **AN8408** |
| **ChrI_A_nidulans_FGSC_A4:2463144-2463194** | **AN6562** |
| **ChrIV_A_nidulans_FGSC_A4:1146115-1146165** | **AN10888** |
| **ChrIV_A_nidulans_FGSC_A4:1189942-1189992** | **AN7052** |
| **ChrVII_A_nidulans_FGSC_A4:3190122-3190172** | **AN2201** |
| **ChrIV_A_nidulans_FGSC_A4:1156832-1156882** | **AN7061** |
| **ChrVIII_A_nidulans_FGSC_A4:1307485-1307535** | **AN1171** |
| **ChrVIII_A_nidulans_FGSC_A4:1307485-1307535** | **AN1172** |
| **ChrV_A_nidulans_FGSC_A4:1350483-1350533** | **AN5163** |
| **ChrVIII_A_nidulans_FGSC_A4:1221473-1221523** | **AN1198** |
| **ChrIII_A_nidulans_FGSC_A4:2044436-2044486** | **AN4439** |
| **ChrIV_A_nidulans_FGSC_A4:2770175-2770225** | **AN7818** |
| **ChrI_A_nidulans_FGSC_A4:1590264-1590314** | **AN5984** |
| **ChrII_A_nidulans_FGSC_A4:60111-60161** | **AN7867** |
| **ChrII_A_nidulans_FGSC_A4:60111-60161** | **AN7866** |
| **ChrV_A_nidulans_FGSC_A4:3151303-3151353** | **AN5321** |
| **ChrVIII_A_nidulans_FGSC_A4:4451754-4451804** | **AN0148** |
| **ChrVIII_A_nidulans_FGSC_A4:4495347-4495397** | **AN0133** |
| **ChrVIII_A_nidulans_FGSC_A4:4495347-4495397** | **AN0134** |
| **ChrIII_A_nidulans_FGSC_A4:473879-473929** | **AN4951** |
| **ChrII_A_nidulans_FGSC_A4:453499-453549** | **AN12431** |
| **ChrIV_A_nidulans_FGSC_A4:630405-630455** | **AN12267** |
| **ChrI_A_nidulans_FGSC_A4:3073307-3073357** | **AN6755** |
| **ChrII_A_nidulans_FGSC_A4:3961472-3961522** | **AN3504** |
| **ChrII_A_nidulans_FGSC_A4:3961472-3961522** | **AN3503** |
| **ChrIV_A_nidulans_FGSC_A4:1588064-1588114** | **AN11745** |
| **ChrVII_A_nidulans_FGSC_A4:3803761-3803811** | **AN2399** |
| **ChrI_A_nidulans_FGSC_A4:2520305-2520355** | **AN6579** |
| **ChrI_A_nidulans_FGSC_A4:2520305-2520355** | **AN6580** |
| **ChrVII_A_nidulans_FGSC_A4:3523365-3523415** | **AN2312** |
| **ChrVI_A_nidulans_FGSC_A4:1816322-1816372** | **AN3103** |
| **ChrI_A_nidulans_FGSC_A4:2395530-2395580** | **AN12122** |
| **ChrVIII_A_nidulans_FGSC_A4:2175659-2175709** | **AN0885** |
| **ChrVI_A_nidulans_FGSC_A4:2116428-2116478** | **AN3009** |
| **ChrV_A_nidulans_FGSC_A4:331194-331244** | **AN8388** |
| **ChrVIII_A_nidulans_FGSC_A4:2929188-2929238** | **AN0636** |
| **ChrII_A_nidulans_FGSC_A4:3502087-3502137** | **AN3646** |
| **ChrI_A_nidulans_FGSC_A4:2577835-2577885** | **AN6597** |
| **ChrII_A_nidulans_FGSC_A4:2256208-2256258** | **AN4043** |
| **ChrII_A_nidulans_FGSC_A4:2256208-2256258** | **AN10498** |
| **ChrVI_A_nidulans_FGSC_A4:2833414-2833464** | **AN2777** |
| **ChrII_A_nidulans_FGSC_A4:1176382-1176432** | **AN11058** |
| **ChrVII_A_nidulans_FGSC_A4:3006628-3006678** | **AN12197** |
| **ChrV_A_nidulans_FGSC_A4:796567-796617** | **AN8538** |
| **ChrIII_A_nidulans_FGSC_A4:1103253-1103303** | **AN4749** |
| **ChrIII_A_nidulans_FGSC_A4:1103253-1103303** | **AN4750** |
| **ChrII_A_nidulans_FGSC_A4:1771796-1771846** | **AN10522** |
| **ChrI_A_nidulans_FGSC_A4:2600418-2600468** | **AN10835** |
| **ChrVII_A_nidulans_FGSC_A4:2972376-2972426** | **AN11869** |
| **ChrVI_A_nidulans_FGSC_A4:2222886-2222936** | **AN10363** |
| **ChrV_A_nidulans_FGSC_A4:3309735-3309785** | **AN10656** |
| **ChrVII_A_nidulans_FGSC_A4:250455-250505** | **AN8973** |
| **ChrV_A_nidulans_FGSC_A4:1857574-1857624** | **AN5731** |
| **ChrV_A_nidulans_FGSC_A4:208978-209028** | **AN8355** |
| **ChrIII_A_nidulans_FGSC_A4:1739073-1739123** | **AN10567** |
| **ChrI_A_nidulans_FGSC_A4:2355428-2355478** | **AN10825** |
| **ChrVI_A_nidulans_FGSC_A4:2844830-2844880** | **AN2773** |
| **ChrIII_A_nidulans_FGSC_A4:1109060-1109110** | **AN4748** |
| **ChrIII_A_nidulans_FGSC_A4:2010362-2010412** | **AN4452** |
| **ChrVIII_A_nidulans_FGSC_A4:3036200-3036250** | **AN0598** |
| **ChrVIII_A_nidulans_FGSC_A4:3036200-3036250** | **AN0597** |
| **ChrVII_A_nidulans_FGSC_A4:2919633-2919683** | **AN2118** |
| **ChrVI_A_nidulans_FGSC_A4:2497475-2497525** | **AN2886** |
| **ChrV_A_nidulans_FGSC_A4:1845061-1845111** | **AN5737** |
| **ChrI_A_nidulans_FGSC_A4:2101476-2101526** | **AN10746** |
| **ChrVIII_A_nidulans_FGSC_A4:2809083-2809133** | **AN0681** |
| **ChrVI_A_nidulans_FGSC_A4:925644-925694** | **AN3374** |
| **ChrVI_A_nidulans_FGSC_A4:1726322-1726372** | **AN3131** |
| **ChrVI_A_nidulans_FGSC_A4:1726322-1726372** | **AN3132** |
| **ChrIV_A_nidulans_FGSC_A4:1947831-1947881** | **AN7556** |
| **ChrVI_A_nidulans_FGSC_A4:170460-170510** | **AN9183** |
| **ChrIII_A_nidulans_FGSC_A4:379181-379231** | **AN11772** |
| **ChrI_A_nidulans_FGSC_A4:853311-853361** | **AN12358** |
| **ChrVIII_A_nidulans_FGSC_A4:1563080-1563130** | **AN11841** |
| **ChrII_A_nidulans_FGSC_A4:3466379-3466429** | **AN11722** |
| **ChrII_A_nidulans_FGSC_A4:3976589-3976639** | **AN3498** |
| **ChrII_A_nidulans_FGSC_A4:3976589-3976639** | **AN3499** |
| **ChrVII_A_nidulans_FGSC_A4:2393907-2393957** | **AN11803** |
| **ChrIV_A_nidulans_FGSC_A4:1971897-1971947** | **AN7567** |
| **ChrV_A_nidulans_FGSC_A4:883759-883809** | **AN12258** |
| **ChrIII_A_nidulans_FGSC_A4:1594164-1594214** | **AN4589** |
| **ChrIV_A_nidulans_FGSC_A4:2767884-2767934** | **AN11021** |
| **ChrVIII_A_nidulans_FGSC_A4:4701496-4701546** | **AN0069** |
| **ChrVI_A_nidulans_FGSC_A4:2308061-2308111** | **AN10361** |
| **ChrII_A_nidulans_FGSC_A4:1837119-1837169** | **AN10514** |
| **ChrV_A_nidulans_FGSC_A4:603193-603243** | **AN8474** |
| **ChrVIII_A_nidulans_FGSC_A4:4726264-4726314** | **AN0058** |
| **ChrVIII_A_nidulans_FGSC_A4:4726264-4726314** | **AN0059** |
| **ChrI_A_nidulans_FGSC_A4:483719-483769** | **AN6338** |
| **ChrVII_A_nidulans_FGSC_A4:2648001-2648051** | **AN2032** |
| **ChrVII_A_nidulans_FGSC_A4:3305608-3305658** | **AN2245** |
| **ChrIII_A_nidulans_FGSC_A4:3236334-3236384** | **AN8657** |
| **ChrI_A_nidulans_FGSC_A4:974520-974570** | **AN6193** |
| **ChrVIII_A_nidulans_FGSC_A4:3732826-3732876** | **AN0377** |
| **ChrIV_A_nidulans_FGSC_A4:1034765-1034815** | **AN10891** |
| **ChrIII_A_nidulans_FGSC_A4:1062722-1062772** | **AN10584** |
| **ChrI_A_nidulans_FGSC_A4:404497-404547** | **AN6365** |
| **ChrI_A_nidulans_FGSC_A4:404497-404547** | **AN6366** |
| **ChrV_A_nidulans_FGSC_A4:597533-597583** | **AN8472** |
| **ChrIII_A_nidulans_FGSC_A4:2403672-2403722** | **AN10545** |
| **ChrIV_A_nidulans_FGSC_A4:130358-130408** | **AN10933** |
| **ChrI_A_nidulans_FGSC_A4:1542643-1542693** | **AN6000** |
| **ChrVIII_A_nidulans_FGSC_A4:4320964-4321014** | **AN0196** |
| **ChrV_A_nidulans_FGSC_A4:3175820-3175870** | **AN5314** |
| **ChrIV_A_nidulans_FGSC_A4:237905-237955** | **AN7345** |
| **ChrVII_A_nidulans_FGSC_A4:1995894-1995944** | **AN1821** |
| **ChrIII_A_nidulans_FGSC_A4:1725372-1725422** | **AN4536** |
| **ChrIV_A_nidulans_FGSC_A4:1578894-1578944** | **AN7449** |
| **ChrVII_A_nidulans_FGSC_A4:4407494-4407544** | **AN2576** |
| **ChrVI_A_nidulans_FGSC_A4:990858-990908** | **AN3354** |
| **ChrVI_A_nidulans_FGSC_A4:990858-990908** | **AN3355** |
| **ChrV_A_nidulans_FGSC_A4:1804341-1804391** | **AN5752** |
| **ChrV_A_nidulans_FGSC_A4:1804341-1804391** | **AN5753** |
| **ChrVII_A_nidulans_FGSC_A4:58890-58940** | **AN9041** |
| **ChrII_A_nidulans_FGSC_A4:165085-165135** | **AN7894** |
| **ChrII_A_nidulans_FGSC_A4:165085-165135** | **AN7893** |
| **ChrV_A_nidulans_FGSC_A4:2015538-2015588** | **AN5672** |
| **ChrV_A_nidulans_FGSC_A4:2015538-2015588** | **AN5673** |
| **ChrIII_A_nidulans_FGSC_A4:2381143-2381193** | **AN10546** |
| **ChrIV_A_nidulans_FGSC_A4:1386279-1386329** | **AN10872** |
| **ChrVIII_A_nidulans_FGSC_A4:1545500-1545550** | **AN1092** |
| **ChrVIII_A_nidulans_FGSC_A4:4518777-4518827** | **AN10016** |
| **ChrVIII_A_nidulans_FGSC_A4:4518777-4518827** | **AN0126** |
| **ChrVII_A_nidulans_FGSC_A4:2466282-2466332** | **AN10248** |
| **ChrV_A_nidulans_FGSC_A4:2233603-2233653** | **AN9474** |
| **ChrII_A_nidulans_FGSC_A4:3557096-3557146** | **AN3626** |
| **ChrVI_A_nidulans_FGSC_A4:221745-221795** | **AN9168** |
| **ChrVI_A_nidulans_FGSC_A4:2492261-2492311** | **AN2889** |
| **ChrVI_A_nidulans_FGSC_A4:2562690-2562740** | **AN2867** |
| **ChrII_A_nidulans_FGSC_A4:3227701-3227751** | **AN3737** |
| **ChrIII_A_nidulans_FGSC_A4:2885963-2886013** | **AN8766** |
| **ChrV_A_nidulans_FGSC_A4:2184854-2184904** | **AN5623** |
| **ChrIII_A_nidulans_FGSC_A4:1784842-1784892** | **AN4521** |
| **ChrIII_A_nidulans_FGSC_A4:2191306-2191356** | **AN4386** |
| **ChrIII_A_nidulans_FGSC_A4:3255105-3255155** | **AN11094** |
| **ChrI_A_nidulans_FGSC_A4:2901646-2901696** | **AN6701** |
| **ChrVIII_A_nidulans_FGSC_A4:1067357-1067407** | **AN1244** |
| **ChrVI_A_nidulans_FGSC_A4:1612668-1612718** | **AN3164** |
| **ChrI_A_nidulans_FGSC_A4:1882151-1882201** | **AN5893** |
| **ChrVI_A_nidulans_FGSC_A4:64147-64197** | **AN11199** |
| **ChrVI_A_nidulans_FGSC_A4:3113058-3113108** | **AN2698** |
| **ChrVI_A_nidulans_FGSC_A4:2730874-2730924** | **AN2814** |
| **ChrIII_A_nidulans_FGSC_A4:1526675-1526725** | **AN4612** |
| **ChrIII_A_nidulans_FGSC_A4:1526675-1526725** | **AN11444** |
| **ChrI_A_nidulans_FGSC_A4:613997-614047** | **AN6303** |
| **ChrI_A_nidulans_FGSC_A4:613997-614047** | **AN6302** |
| **ChrI_A_nidulans_FGSC_A4:1190560-1190610** | **AN6123** |
| **ChrI_A_nidulans_FGSC_A4:1190560-1190610** | **AN11500** |
| **ChrI_A_nidulans_FGSC_A4:2536114-2536164** | **AN6585** |
| **ChrI_A_nidulans_FGSC_A4:2536114-2536164** | **AN6584** |
| **ChrVII_A_nidulans_FGSC_A4:3053132-3053182** | **AN2154** |
| **ChrVI_A_nidulans_FGSC_A4:196656-196706** | **AN11196** |
| **ChrVI_A_nidulans_FGSC_A4:1293465-1293515** | **AN3265** |
| **ChrVI_A_nidulans_FGSC_A4:3132846-3132896** | **AN10334** |
| **ChrV_A_nidulans_FGSC_A4:2514055-2514105** | **AN10677** |
| **ChrV_A_nidulans_FGSC_A4:2693116-2693166** | **AN5474** |
| **ChrIII_A_nidulans_FGSC_A4:2354365-2354415** | **AN4335** |
| **ChrIV_A_nidulans_FGSC_A4:2703323-2703373** | **AN12086** |
| **ChrV_A_nidulans_FGSC_A4:2460675-2460725** | **AN5545** |
| **ChrII_A_nidulans_FGSC_A4:2525188-2525238** | **AN3954** |
| **ChrII_A_nidulans_FGSC_A4:2525188-2525238** | **AN3953** |
| **ChrVII_A_nidulans_FGSC_A4:3129781-3129831** | **AN10272** |
| **ChrVII_A_nidulans_FGSC_A4:3129781-3129831** | **AN2182** |
| **ChrIII_A_nidulans_FGSC_A4:627304-627354** | **AN4898** |
| **ChrVI_A_nidulans_FGSC_A4:2003667-2003717** | **AN11701** |
| **ChrIV_A_nidulans_FGSC_A4:673416-673466** | **AN10906** |
| **ChrI_A_nidulans_FGSC_A4:498506-498556** | **AN6332** |
| **ChrVII_A_nidulans_FGSC_A4:1809482-1809532** | **AN1757** |
| **ChrV_A_nidulans_FGSC_A4:2795933-2795983** | **AN5445** |
| **ChrIV_A_nidulans_FGSC_A4:2747834-2747884** | **AN7809** |
| **ChrIV_A_nidulans_FGSC_A4:2747834-2747884** | **AN12090** |
| **ChrVI_A_nidulans_FGSC_A4:3016119-3016169** | **AN12313** |
| **ChrV_A_nidulans_FGSC_A4:686072-686122** | **AN8496** |
| **ChrIV_A_nidulans_FGSC_A4:1003399-1003449** | **AN7105** |
| **ChrIV_A_nidulans_FGSC_A4:1025869-1025919** | **AN7098** |
| **ChrV_A_nidulans_FGSC_A4:701048-701098** | **AN8503** |
| **ChrIV_A_nidulans_FGSC_A4:992563-992613** | **AN7109** |
| **ChrIV_A_nidulans_FGSC_A4:992563-992613** | **AN7110** |
| **ChrI_A_nidulans_FGSC_A4:1845428-1845478** | **AN5906** |
| **ChrVIII_A_nidulans_FGSC_A4:1192612-1192662** | **AN1209** |
| **ChrV_A_nidulans_FGSC_A4:2422381-2422431** | **AN5555** |
| **ChrII_A_nidulans_FGSC_A4:3531697-3531747** | **AN3635** |
| **ChrIV_A_nidulans_FGSC_A4:2090495-2090545** | **AN7598** |
| **ChrVIII_A_nidulans_FGSC_A4:2690074-2690124** | **AN0717** |
| **ChrV_A_nidulans_FGSC_A4:2141542-2141592** | **AN5634** |
| **ChrI_A_nidulans_FGSC_A4:2654148-2654198** | **AN6620** |
| **ChrIII_A_nidulans_FGSC_A4:2050167-2050217** | **AN10555** |
| **ChrII_A_nidulans_FGSC_A4:2190125-2190175** | **AN4067** |
| **ChrII_A_nidulans_FGSC_A4:2190125-2190175** | **AN4068** |
| **ChrVIII_A_nidulans_FGSC_A4:1556289-1556339** | **AN1089** |
| **ChrVIII_A_nidulans_FGSC_A4:2225672-2225722** | **AN0867** |
| **ChrVII_A_nidulans_FGSC_A4:2726609-2726659** | **AN11862** |
| **ChrV_A_nidulans_FGSC_A4:910567-910617** | **AN11622** |
| **ChrI_A_nidulans_FGSC_A4:2943601-2943651** | **AN6712** |
| **ChrIII_A_nidulans_FGSC_A4:1335764-1335814** | **AN4678** |
| **ChrI_A_nidulans_FGSC_A4:1052331-1052381** | **AN6162** |
| **ChrVIII_A_nidulans_FGSC_A4:1004237-1004287** | **AN1267** |
| **ChrVII_A_nidulans_FGSC_A4:147626-147676** | **AN9007** |
| **ChrV_A_nidulans_FGSC_A4:1309247-1309297** | **AN5153** |
| **ChrVIII_A_nidulans_FGSC_A4:1614958-1615008** | **AN11292** |
| **ChrIII_A_nidulans_FGSC_A4:2812686-2812736** | **AN8783** |
| **ChrII_A_nidulans_FGSC_A4:2819964-2820014** | **AN3872** |
| **ChrVIII_A_nidulans_FGSC_A4:166109-166159** | **AN9281** |
| **ChrVIII_A_nidulans_FGSC_A4:520770-520820** | **AN9401** |
| **ChrVIII_A_nidulans_FGSC_A4:2526403-2526453** | **AN10119** |
| **ChrVII_A_nidulans_FGSC_A4:24144-24194** | **AN9054** |
| **ChrVII_A_nidulans_FGSC_A4:4113483-4113533** | **AN2489** |
| **ChrV_A_nidulans_FGSC_A4:64476-64526** | **AN8307** |
| **ChrV_A_nidulans_FGSC_A4:64476-64526** | **AN8306** |
| **ChrIV_A_nidulans_FGSC_A4:2484078-2484128** | **AN7724** |
| **ChrIV_A_nidulans_FGSC_A4:2484078-2484128** | **AN7725** |
| **ChrIV_A_nidulans_FGSC_A4:2553094-2553144** | **AN7741** |
| **ChrVII_A_nidulans_FGSC_A4:2079307-2079357** | **AN1855** |
| **ChrVI_A_nidulans_FGSC_A4:2582115-2582165** | **AN2860** |
| **ChrIII_A_nidulans_FGSC_A4:3375361-3375411** | **AN8611** |
| **ChrVII_A_nidulans_FGSC_A4:3201879-3201929** | **AN10277** |
| **ChrIV_A_nidulans_FGSC_A4:963687-963737** | **AN7118** |
| **ChrI_A_nidulans_FGSC_A4:1644783-1644833** | **AN5967** |
| **ChrVIII_A_nidulans_FGSC_A4:470777-470827** | **AN9383** |
| **ChrVII_A_nidulans_FGSC_A4:528009-528059** | **AN12239** |
| **ChrVII_A_nidulans_FGSC_A4:528009-528059** | **AN8894** |
| **ChrV_A_nidulans_FGSC_A4:2556511-2556561** | **AN5513** |
| **ChrII_A_nidulans_FGSC_A4:2895971-2896021** | **AN3840** |
| **ChrII_A_nidulans_FGSC_A4:3828482-3828532** | **AN12357** |
| **ChrVIII_A_nidulans_FGSC_A4:1750865-1750915** | **AN1030** |
| **ChrV_A_nidulans_FGSC_A4:851247-851297** | **AN8554** |
| **ChrII_A_nidulans_FGSC_A4:3500740-3500790** | **AN10449** |
| **ChrII_A_nidulans_FGSC_A4:3682541-3682591** | **AN3587** |
| **ChrVII_A_nidulans_FGSC_A4:520429-520479** | **AN8897** |
| **ChrV_A_nidulans_FGSC_A4:571224-571274** | **AN8465** |
| **ChrI_A_nidulans_FGSC_A4:2108516-2108566** | **AN5833** |
| **ChrI_A_nidulans_FGSC_A4:1682589-1682639** | **AN5957** |
| **ChrVII_A_nidulans_FGSC_A4:1763310-1763360** | **AN1739** |
| **ChrVI_A_nidulans_FGSC_A4:772455-772505** | **AN3422** |
| **ChrVI_A_nidulans_FGSC_A4:772455-772505** | **AN3423** |
| **ChrVI_A_nidulans_FGSC_A4:3239572-3239622** | **AN2659** |
| **ChrV_A_nidulans_FGSC_A4:2827890-2827940** | **AN5434** |
| **ChrVIII_A_nidulans_FGSC_A4:1858352-1858402** | **AN10138** |
| **ChrIV_A_nidulans_FGSC_A4:2054443-2054493** | **AN7588** |
| **ChrVII_A_nidulans_FGSC_A4:263596-263646** | **AN8968** |
| **ChrVII_A_nidulans_FGSC_A4:1185362-1185412** | **AN1563** |
| **ChrV_A_nidulans_FGSC_A4:3015582-3015632** | **AN5371** |
| **ChrIV_A_nidulans_FGSC_A4:2372824-2372874** | **AN11003** |
| **ChrIV_A_nidulans_FGSC_A4:2481287-2481337** | **AN7722** |
| **ChrIV_A_nidulans_FGSC_A4:2481287-2481337** | **AN7723** |
| **ChrVIII_A_nidulans_FGSC_A4:3512562-3512612** | **AN0450** |
| **ChrVII_A_nidulans_FGSC_A4:1713886-1713936** | **AN1721** |
| **ChrII_A_nidulans_FGSC_A4:2891851-2891901** | **AN3843** |
| **ChrII_A_nidulans_FGSC_A4:3762163-3762213** | **AN3559** |
| **ChrI_A_nidulans_FGSC_A4:1459905-1459955** | **AN6028** |
| **ChrVIII_A_nidulans_FGSC_A4:2469170-2469220** | **AN11281** |
| **ChrVII_A_nidulans_FGSC_A4:673487-673537** | **AN9420** |
| **ChrVII_A_nidulans_FGSC_A4:3797294-3797344** | **AN2396** |
| **ChrVI_A_nidulans_FGSC_A4:2816825-2816875** | **AN11365** |
| **ChrVI_A_nidulans_FGSC_A4:2816825-2816875** | **AN2784** |
| **ChrV_A_nidulans_FGSC_A4:1202487-1202537** | **AN5116** |
| **ChrI_A_nidulans_FGSC_A4:2336094-2336144** | **AN10832** |
| **ChrIII_A_nidulans_FGSC_A4:1582696-1582746** | **AN4591** |
| **ChrVIII_A_nidulans_FGSC_A4:4472787-4472837** | **AN0142** |
| **ChrVI_A_nidulans_FGSC_A4:1307930-1307980** | **AN12187** |
| **ChrVI_A_nidulans_FGSC_A4:1307930-1307980** | **AN3261** |
| **ChrIII_A_nidulans_FGSC_A4:2913255-2913305** | **AN11124** |
| **ChrIII_A_nidulans_FGSC_A4:2913255-2913305** | **AN8755** |
| **ChrV_A_nidulans_FGSC_A4:2029530-2029580** | **AN5669** |
| **ChrIII_A_nidulans_FGSC_A4:421837-421887** | **AN10620** |
| **ChrVIII_A_nidulans_FGSC_A4:1745586-1745636** | **AN1033** |
| **ChrVII_A_nidulans_FGSC_A4:4148528-4148578** | **AN2501** |
| **ChrVII_A_nidulans_FGSC_A4:4148528-4148578** | **AN2500** |
| **ChrII_A_nidulans_FGSC_A4:3888542-3888592** | **AN12487** |
| **ChrIV_A_nidulans_FGSC_A4:167066-167116** | **AN7368** |
| **ChrIV_A_nidulans_FGSC_A4:167066-167116** | **AN7367** |
| **ChrI_A_nidulans_FGSC_A4:3516677-3516727** | **AN6900** |
| **ChrVIII_A_nidulans_FGSC_A4:2451490-2451540** | **AN0797** |
| **ChrVII_A_nidulans_FGSC_A4:1068385-1068435** | **AN1526** |
| **ChrVII_A_nidulans_FGSC_A4:1068385-1068435** | **AN1525** |
| **ChrVI_A_nidulans_FGSC_A4:1591514-1591564** | **AN3172** |
| **ChrII_A_nidulans_FGSC_A4:2224365-2224415** | **AN4053** |
| **ChrII_A_nidulans_FGSC_A4:2224365-2224415** | **AN4054** |
| **ChrIV_A_nidulans_FGSC_A4:100967-101017** | **AN7392** |
| **ChrI_A_nidulans_FGSC_A4:2953149-2953199** | **AN6715** |
| **ChrI_A_nidulans_FGSC_A4:2953149-2953199** | **AN6716** |
| **ChrVII_A_nidulans_FGSC_A4:1231057-1231107** | **AN1577** |
| **ChrV_A_nidulans_FGSC_A4:2150350-2150400** | **AN5631** |
| **ChrV_A_nidulans_FGSC_A4:2150350-2150400** | **AN5632** |
| **ChrVII_A_nidulans_FGSC_A4:2866242-2866292** | **AN2103** |
| **ChrVII_A_nidulans_FGSC_A4:1315871-1315921** | **AN1601** |
| **ChrVII_A_nidulans_FGSC_A4:1315871-1315921** | **AN9444** |
| **ChrII_A_nidulans_FGSC_A4:2032470-2032520** | **AN4119** |
| **ChrIV_A_nidulans_FGSC_A4:1133663-1133713** | **AN10889** |
| **ChrII_A_nidulans_FGSC_A4:2548971-2549021** | **AN3948** |
| **ChrV_A_nidulans_FGSC_A4:2315749-2315799** | **AN5586** |
| **ChrII_A_nidulans_FGSC_A4:1765958-1766008** | **AN4201** |
| **ChrIII_A_nidulans_FGSC_A4:2754137-2754187** | **AN8806** |
| **ChrVII_A_nidulans_FGSC_A4:865565-865615** | **AN10197** |
| **ChrVI_A_nidulans_FGSC_A4:252331-252381** | **AN9156** |
| **ChrIII_A_nidulans_FGSC_A4:763211-763261** | **AN4857** |
| **ChrII_A_nidulans_FGSC_A4:1932532-1932582** | **AN4152** |
| **ChrIV_A_nidulans_FGSC_A4:813865-813915** | **AN12148** |
| **ChrI_A_nidulans_FGSC_A4:497892-497942** | **AN6332** |
| **ChrVIII_A_nidulans_FGSC_A4:1596260-1596310** | **AN1074** |
| **ChrVIII_A_nidulans_FGSC_A4:1861974-1862024** | **AN0992** |
| **ChrVIII_A_nidulans_FGSC_A4:1861974-1862024** | **AN0991** |
| **ChrVII_A_nidulans_FGSC_A4:3705129-3705179** | **AN2367** |
| **ChrVII_A_nidulans_FGSC_A4:3705129-3705179** | **AN2368** |
| **ChrVI_A_nidulans_FGSC_A4:1383970-1384020** | **AN3234** |
| **ChrV_A_nidulans_FGSC_A4:1172748-1172798** | **AN5105** |
| **ChrIV_A_nidulans_FGSC_A4:2338792-2338842** | **AN7673** |
| **ChrIV_A_nidulans_FGSC_A4:2338792-2338842** | **AN7675** |
| **ChrVII_A_nidulans_FGSC_A4:2370095-2370145** | **AN11802** |
| **ChrIII_A_nidulans_FGSC_A4:2388620-2388670** | **AN4324** |
| **ChrVIII_A_nidulans_FGSC_A4:1754549-1754599** | **AN1028** |
| **ChrVI_A_nidulans_FGSC_A4:2583949-2583999** | **AN2859** |
| **ChrIII_A_nidulans_FGSC_A4:299770-299820** | **AN11775** |
| **ChrIII_A_nidulans_FGSC_A4:299770-299820** | **AN5008** |
| **ChrII_A_nidulans_FGSC_A4:3461381-3461431** | **AN10434** |
| **ChrII_A_nidulans_FGSC_A4:3536478-3536528** | **AN3632** |
| **ChrI_A_nidulans_FGSC_A4:2125345-2125395** | **AN5828** |
| **ChrVI_A_nidulans_FGSC_A4:2111955-2112005** | **AN3011** |
| **ChrV_A_nidulans_FGSC_A4:1407053-1407103** | **AN5177** |
| **ChrIV_A_nidulans_FGSC_A4:849776-849826** | **AN7151** |
| **ChrVIII_A_nidulans_FGSC_A4:3728914-3728964** | **AN0381** |
| **ChrVII_A_nidulans_FGSC_A4:1106618-1106668** | **AN1541** |
| **ChrVII_A_nidulans_FGSC_A4:2034602-2034652** | **AN1839** |
| **ChrVII_A_nidulans_FGSC_A4:4032518-4032568** | **AN2465** |
| **ChrII_A_nidulans_FGSC_A4:1268449-1268499** | **AN12201** |
| **ChrII_A_nidulans_FGSC_A4:2234094-2234144** | **AN4050** |
| **ChrI_A_nidulans_FGSC_A4:2147534-2147584** | **AN5822** |
| **ChrVII_A_nidulans_FGSC_A4:192263-192313** | **AN8995** |
| **ChrVII_A_nidulans_FGSC_A4:970463-970513** | **AN10209** |
| **ChrVI_A_nidulans_FGSC_A4:779077-779127** | **AN3421** |
| **ChrI_A_nidulans_FGSC_A4:748200-748250** | **AN6254** |
| **ChrVII_A_nidulans_FGSC_A4:2710158-2710208** | **AN2055** |
| **ChrV_A_nidulans_FGSC_A4:2447101-2447151** | **AN5549** |
| **ChrV_A_nidulans_FGSC_A4:2447101-2447151** | **AN10690** |
| **ChrVIII_A_nidulans_FGSC_A4:4555329-4555379** | **AN0117** |
| **ChrVI_A_nidulans_FGSC_A4:2007542-2007592** | **AN11700** |
| **ChrIII_A_nidulans_FGSC_A4:1064575-1064625** | **AN10591** |
| **ChrII_A_nidulans_FGSC_A4:316417-316467** | **AN7952** |
| **ChrII_A_nidulans_FGSC_A4:1633093-1633143** | **AN4246** |
| **ChrI_A_nidulans_FGSC_A4:2299747-2299797** | **AN6499** |
| **ChrVIII_A_nidulans_FGSC_A4:2974593-2974643** | **AN0617** |
| **ChrVII_A_nidulans_FGSC_A4:87882-87932** | **AN9032** |
| **ChrVII_A_nidulans_FGSC_A4:2292974-2293024** | **AN1930** |
| **ChrVII_A_nidulans_FGSC_A4:3779635-3779685** | **AN12114** |
| **ChrVI_A_nidulans_FGSC_A4:1154274-1154324** | **AN3313** |
| **ChrVI_A_nidulans_FGSC_A4:1154274-1154324** | **AN3312** |
| **ChrVI_A_nidulans_FGSC_A4:1747247-1747297** | **AN3124** |
| **ChrVI_A_nidulans_FGSC_A4:3148632-3148682** | **AN2686** |
| **ChrIII_A_nidulans_FGSC_A4:484600-484650** | **AN4945** |
| **ChrVII_A_nidulans_FGSC_A4:1290369-1290419** | **AN1597** |
| **ChrVII_A_nidulans_FGSC_A4:4236845-4236895** | **AN2527** |
| **ChrVII_A_nidulans_FGSC_A4:4236845-4236895** | **AN2526** |
| **ChrIII_A_nidulans_FGSC_A4:873156-873206** | **AN4823** |
| **ChrIV_A_nidulans_FGSC_A4:243097-243147** | **AN7344** |
| **ChrIII_A_nidulans_FGSC_A4:2063086-2063136** | **AN4433** |
| **ChrIII_A_nidulans_FGSC_A4:2063086-2063136** | **AN4434** |
| **ChrII_A_nidulans_FGSC_A4:89412-89462** | **AN7873** |
| **ChrVII_A_nidulans_FGSC_A4:163568-163618** | **AN9003** |
| **ChrII_A_nidulans_FGSC_A4:1002601-1002651** | **AN12171** |
| **ChrIV_A_nidulans_FGSC_A4:123914-123964** | **AN7383** |
| **ChrI_A_nidulans_FGSC_A4:894914-894964** | **AN10784** |
| **ChrVI_A_nidulans_FGSC_A4:1586920-1586970** | **AN10382** |
| **ChrVIII_A_nidulans_FGSC_A4:3106951-3107001** | **AN0579** |
| **ChrVI_A_nidulans_FGSC_A4:2891037-2891087** | **AN2759** |
| **ChrVIII_A_nidulans_FGSC_A4:2685296-2685346** | **AN0718** |
| **ChrIII_A_nidulans_FGSC_A4:857732-857782** | **AN4827** |
| **ChrIII_A_nidulans_FGSC_A4:3088783-3088833** | **AN8696** |
| **ChrIII_A_nidulans_FGSC_A4:3155796-3155846** | **AN8673** |
| **ChrII_A_nidulans_FGSC_A4:1576653-1576703** | **AN4266** |
| **ChrII_A_nidulans_FGSC_A4:1576653-1576703** | **AN4265** |
| **ChrVIII_A_nidulans_FGSC_A4:775003-775053** | **AN1346** |
| **ChrVIII_A_nidulans_FGSC_A4:775003-775053** | **AN1345** |
| **ChrVIII_A_nidulans_FGSC_A4:1145872-1145922** | **AN1222** |
| **ChrVIII_A_nidulans_FGSC_A4:1145872-1145922** | **AN1223** |
| **ChrVIII_A_nidulans_FGSC_A4:2401117-2401167** | **AN10127** |
| **ChrVIII_A_nidulans_FGSC_A4:4484828-4484878** | **AN0136** |
| **ChrVIII_A_nidulans_FGSC_A4:4484828-4484878** | **AN0137** |
| **ChrIII_A_nidulans_FGSC_A4:420642-420692** | **AN4969** |
| **ChrIII_A_nidulans_FGSC_A4:420642-420692** | **AN10610** |
| **ChrV_A_nidulans_FGSC_A4:2409935-2409985** | **AN10695** |
| **ChrV_A_nidulans_FGSC_A4:3194846-3194896** | **AN5308** |
| **ChrIV_A_nidulans_FGSC_A4:995554-995604** | **AN7108** |
| **ChrII_A_nidulans_FGSC_A4:759664-759714** | **AN8102** |
| **ChrVIII_A_nidulans_FGSC_A4:2976987-2977037** | **AN0616** |
| **ChrIII_A_nidulans_FGSC_A4:2769495-2769545** | **AN8799** |
| **ChrVIII_A_nidulans_FGSC_A4:798919-798969** | **AN1335** |
| **ChrVII_A_nidulans_FGSC_A4:2187861-2187911** | **AN1898** |
| **ChrVII_A_nidulans_FGSC_A4:2187861-2187911** | **AN1899** |
| **ChrVIII_A_nidulans_FGSC_A4:641687-641737** | **AN1396** |
| **ChrV_A_nidulans_FGSC_A4:2146271-2146321** | **AN5633** |
| **ChrII_A_nidulans_FGSC_A4:906130-906180** | **AN8149** |
| **ChrV_A_nidulans_FGSC_A4:751078-751128** | **AN8522** |
| **ChrII_A_nidulans_FGSC_A4:243039-243089** | **AN7922** |
| **ChrI_A_nidulans_FGSC_A4:656388-656438** | **AN6284** |
| **ChrI_A_nidulans_FGSC_A4:2485502-2485552** | **AN20053** |
| **ChrI_A_nidulans_FGSC_A4:2485502-2485552** | **AN6570** |
| **ChrI_A_nidulans_FGSC_A4:996059-996109** | **AN6186** |
| **ChrI_A_nidulans_FGSC_A4:996059-996109** | **AN12049** |
| **ChrI_A_nidulans_FGSC_A4:996059-996109** | **AN12050** |
| **ChrVII_A_nidulans_FGSC_A4:1841047-1841097** | **AN1773** |
| **ChrVII_A_nidulans_FGSC_A4:3091732-3091782** | **AN2168** |
| **ChrV_A_nidulans_FGSC_A4:181262-181312** | **AN8343** |
| **ChrIII_A_nidulans_FGSC_A4:474550-474600** | **AN4950** |
| **ChrII_A_nidulans_FGSC_A4:1656187-1656237** | **AN4237** |
| **ChrII_A_nidulans_FGSC_A4:3915360-3915410** | **AN3518** |
| **ChrI_A_nidulans_FGSC_A4:400790-400840** | **AN6367** |
| **ChrI_A_nidulans_FGSC_A4:2691781-2691831** | **AN6636** |
| **ChrVIII_A_nidulans_FGSC_A4:1467150-1467200** | **AN1114** |
| **ChrVIII_A_nidulans_FGSC_A4:3288552-3288602** | **AN0522** |
| **ChrVII_A_nidulans_FGSC_A4:425289-425339** | **AN8923** |
| **ChrVI_A_nidulans_FGSC_A4:1382225-1382275** | **AN3235** |
| **ChrV_A_nidulans_FGSC_A4:3013591-3013641** | **AN5372** |
| **ChrIII_A_nidulans_FGSC_A4:1750338-1750388** | **AN4529** |
| **ChrIII_A_nidulans_FGSC_A4:1750338-1750388** | **AN11442** |
| **ChrII_A_nidulans_FGSC_A4:2372228-2372278** | **AN4002** |
| **ChrVII_A_nidulans_FGSC_A4:1435301-1435351** | **AN1635** |
| **ChrII_A_nidulans_FGSC_A4:479083-479133** | **AN8005** |
| **ChrI_A_nidulans_FGSC_A4:1229153-1229203** | **AN6110** |
| **ChrI_A_nidulans_FGSC_A4:1229153-1229203** | **AN6111** |
| **ChrVIII_A_nidulans_FGSC_A4:3471180-3471230** | **AN0464** |
| **ChrVI_A_nidulans_FGSC_A4:154359-154409** | **AN9191** |
| **ChrII_A_nidulans_FGSC_A4:3101393-3101443** | **AN10468** |
| **ChrIV_A_nidulans_FGSC_A4:573628-573678** | **AN7241** |
| **ChrIII_A_nidulans_FGSC_A4:911-961** | **AN5093** |
| **ChrIII_A_nidulans_FGSC_A4:2367160-2367210** | **AN4330** |
| **ChrII_A_nidulans_FGSC_A4:732254-732304** | **AN8094** |
| **ChrII_A_nidulans_FGSC_A4:3394596-3394646** | **AN3681** |
| **ChrIV_A_nidulans_FGSC_A4:118984-119034** | **AN7385** |
| **ChrIV_A_nidulans_FGSC_A4:2282249-2282299** | **AN10979** |
| **ChrIV_A_nidulans_FGSC_A4:2444605-2444655** | **AN7708** |
| **ChrI_A_nidulans_FGSC_A4:1587523-1587573** | **AN5986** |
| **ChrI_A_nidulans_FGSC_A4:3357430-3357480** | **AN6844** |
| **ChrVIII_A_nidulans_FGSC_A4:766300-766350** | **AN1351** |
| **ChrVII_A_nidulans_FGSC_A4:433621-433671** | **AN8920** |
| **ChrVII_A_nidulans_FGSC_A4:497097-497147** | **AN8907** |
| **ChrVIII_A_nidulans_FGSC_A4:1718162-1718212** | **AN1037** |
| **ChrVIII_A_nidulans_FGSC_A4:1718162-1718212** | **AN11288** |
| **ChrVIII_A_nidulans_FGSC_A4:2116977-2117027** | **AN0904** |
| **ChrII_A_nidulans_FGSC_A4:1367327-1367377** | **AN8280** |
| **ChrI_A_nidulans_FGSC_A4:716710-716760** | **AN6264** |
| **ChrVIII_A_nidulans_FGSC_A4:2972546-2972596** | **AN0619** |
| **ChrVIII_A_nidulans_FGSC_A4:2972546-2972596** | **AN0618** |
| **ChrII_A_nidulans_FGSC_A4:1265968-1266018** | **AN8246** |
| **ChrVII_A_nidulans_FGSC_A4:1274856-1274906** | **AN1591** |
| **ChrVII_A_nidulans_FGSC_A4:1274856-1274906** | **AN1590** |
| **ChrII_A_nidulans_FGSC_A4:2198609-2198659** | **AN4064** |
| **ChrIII_A_nidulans_FGSC_A4:894085-894135** | **AN4819** |
| **ChrII_A_nidulans_FGSC_A4:2176031-2176081** | **AN4074** |
| **ChrI_A_nidulans_FGSC_A4:480562-480612** | **AN6339** |
| **ChrVIII_A_nidulans_FGSC_A4:3032931-3032981** | **AN0600** |
| **ChrVIII_A_nidulans_FGSC_A4:3653290-3653340** | **AN0408** |
| **ChrVIII_A_nidulans_FGSC_A4:3808552-3808602** | **AN0356** |
| **ChrVI_A_nidulans_FGSC_A4:690296-690346** | **AN3446** |
| **ChrV_A_nidulans_FGSC_A4:651249-651299** | **AN8485** |
| **ChrV_A_nidulans_FGSC_A4:1523889-1523939** | **AN5217** |
| **ChrII_A_nidulans_FGSC_A4:3729645-3729695** | **AN10463** |
| **ChrIV_A_nidulans_FGSC_A4:1095817-1095867** | **AN7081** |
| **ChrVII_A_nidulans_FGSC_A4:829189-829239** | **AN1454** |
| **ChrVII_A_nidulans_FGSC_A4:3285662-3285712** | **AN2236** |
| **ChrVI_A_nidulans_FGSC_A4:1049278-1049328** | **AN3339** |
| **ChrVIII_A_nidulans_FGSC_A4:4118609-4118659** | **AN0259** |
| **ChrVIII_A_nidulans_FGSC_A4:4118609-4118659** | **AN0260** |
| **ChrI_A_nidulans_FGSC_A4:63708-63758** | **AN10812** |
| **ChrI_A_nidulans_FGSC_A4:63708-63758** | **AN6468** |
| **ChrI_A_nidulans_FGSC_A4:2340356-2340406** | **AN6513** |
| **ChrVIII_A_nidulans_FGSC_A4:1908472-1908522** | **AN0977** |
| **ChrVIII_A_nidulans_FGSC_A4:4438707-4438757** | **AN10044** |
| **ChrVIII_A_nidulans_FGSC_A4:4438707-4438757** | **AN10038** |
| **ChrVII_A_nidulans_FGSC_A4:2615882-2615932** | **AN2024** |
| **ChrVII_A_nidulans_FGSC_A4:2846687-2846737** | **AN10264** |
| **ChrVI_A_nidulans_FGSC_A4:391004-391054** | **AN9116** |
| **ChrVI_A_nidulans_FGSC_A4:971124-971174** | **AN3359** |
| **ChrII_A_nidulans_FGSC_A4:3213996-3214046** | **AN3742** |
| **ChrI_A_nidulans_FGSC_A4:2770886-2770936** | **AN6663** |
| **ChrVII_A_nidulans_FGSC_A4:1839313-1839363** | **AN1772** |
| **ChrI_A_nidulans_FGSC_A4:2562333-2562383** | **AN6591** |
| **ChrVII_A_nidulans_FGSC_A4:3296041-3296091** | **AN2240** |
| **ChrIII_A_nidulans_FGSC_A4:2903581-2903631** | **AN11112** |
| **ChrIII_A_nidulans_FGSC_A4:3039742-3039792** | **AN8716** |
| **ChrIII_A_nidulans_FGSC_A4:1956580-1956630** | **AN4469** |
| **ChrII_A_nidulans_FGSC_A4:698496-698546** | **AN8083** |
| **ChrII_A_nidulans_FGSC_A4:2623643-2623693** | **AN3928** |
| **ChrIV_A_nidulans_FGSC_A4:758388-758438** | **AN7180** |
| **ChrVII_A_nidulans_FGSC_A4:476874-476924** | **AN8911** |
| **ChrIV_A_nidulans_FGSC_A4:487026-487076** | **AN12400** |
| **ChrIV_A_nidulans_FGSC_A4:2180256-2180306** | **AN7625** |
| **ChrI_A_nidulans_FGSC_A4:804901-804951** | **AN6234** |
| **ChrI_A_nidulans_FGSC_A4:804901-804951** | **AN6236** |
| **ChrVIII_A_nidulans_FGSC_A4:1456412-1456462** | **AN1118** |
| **ChrVIII_A_nidulans_FGSC_A4:1942468-1942518** | **AN0964** |
| **ChrVIII_A_nidulans_FGSC_A4:2379217-2379267** | **AN12110** |
| **ChrVIII_A_nidulans_FGSC_A4:3959326-3959376** | **AN0307** |
| **ChrVIII_A_nidulans_FGSC_A4:4684835-4684885** | **AN0076** |
| **ChrVII_A_nidulans_FGSC_A4:92709-92759** | **AN9028** |
| **ChrVII_A_nidulans_FGSC_A4:864199-864249** | **AN10192** |
| **ChrV_A_nidulans_FGSC_A4:2131016-2131066** | **AN5637** |
| **ChrV_A_nidulans_FGSC_A4:3276715-3276765** | **AN5282** |
| **ChrI_A_nidulans_FGSC_A4:1718955-1719005** | **AN5944** |
| **ChrVII_A_nidulans_FGSC_A4:1209086-1209136** | **AN1571** |
| **ChrIV_A_nidulans_FGSC_A4:2318234-2318284** | **AN7666** |
| **ChrI_A_nidulans_FGSC_A4:770455-770505** | **AN6245** |
| **ChrVIII_A_nidulans_FGSC_A4:950557-950607** | **AN1285** |
| **ChrVI_A_nidulans_FGSC_A4:1406758-1406808** | **AN3226** |
| **ChrV_A_nidulans_FGSC_A4:3236301-3236351** | **AN11228** |
| **ChrV_A_nidulans_FGSC_A4:3236301-3236351** | **AN11950** |
| **ChrII_A_nidulans_FGSC_A4:2131227-2131277** | **AN12323** |
| **ChrII_A_nidulans_FGSC_A4:2131227-2131277** | **AN9533** |
| **ChrI_A_nidulans_FGSC_A4:1249047-1249097** | **AN6104** |
| **ChrV_A_nidulans_FGSC_A4:680426-680476** | **AN8495** |
| **ChrIII_A_nidulans_FGSC_A4:1074099-1074149** | **AN4762** |
| **ChrII_A_nidulans_FGSC_A4:1854150-1854200** | **AN10520** |
| **ChrIV_A_nidulans_FGSC_A4:1585318-1585368** | **AN7451** |
| **ChrII_A_nidulans_FGSC_A4:2155590-2155640** | **AN4080** |
| **ChrI_A_nidulans_FGSC_A4:3029847-3029897** | **AN6739** |
| **ChrVIII_A_nidulans_FGSC_A4:236691-236741** | **AN9307** |
| **ChrVIII_A_nidulans_FGSC_A4:2816062-2816112** | **AN0677** |
| **ChrVIII_A_nidulans_FGSC_A4:4330522-4330572** | **AN0191** |
| **ChrVIII_A_nidulans_FGSC_A4:4330522-4330572** | **AN0192** |
| **ChrVI_A_nidulans_FGSC_A4:2427067-2427117** | **AN2911** |
| **ChrV_A_nidulans_FGSC_A4:870626-870676** | **AN8561** |
| **ChrV_A_nidulans_FGSC_A4:1595452-1595502** | **AN5241** |
| **ChrIII_A_nidulans_FGSC_A4:3015947-3015997** | **AN8725** |
| **ChrVIII_A_nidulans_FGSC_A4:1219107-1219157** | **AN1199** |
| **ChrVII_A_nidulans_FGSC_A4:1928650-1928700** | **AN1800** |
| **ChrIII_A_nidulans_FGSC_A4:1120528-1120578** | **AN4743** |
| **ChrIII_A_nidulans_FGSC_A4:3230089-3230139** | **AN12028** |
| **ChrVIII_A_nidulans_FGSC_A4:2742061-2742111** | **AN0703** |
| **ChrIII_A_nidulans_FGSC_A4:181002-181052** | **AN10623** |
| **ChrI_A_nidulans_FGSC_A4:1109348-1109398** | **AN6140** |
| **ChrI_A_nidulans_FGSC_A4:1839175-1839225** | **AN5909** |
| **ChrVI_A_nidulans_FGSC_A4:1600413-1600463** | **AN3169** |
| **ChrIV_A_nidulans_FGSC_A4:2761953-2762003** | **AN7814** |
| **ChrI_A_nidulans_FGSC_A4:1449600-1449650** | **AN6033** |
| **ChrVI_A_nidulans_FGSC_A4:2644840-2644890** | **AN2836** |
| **ChrIII_A_nidulans_FGSC_A4:2282983-2283033** | **AN4361** |
| **ChrII_A_nidulans_FGSC_A4:1148213-1148263** | **AN12403** |
| **ChrII_A_nidulans_FGSC_A4:1267422-1267472** | **AN8248** |
| **ChrIV_A_nidulans_FGSC_A4:2157272-2157322** | **AN7618** |
| **ChrI_A_nidulans_FGSC_A4:3206011-3206061** | **AN6798** |
| **ChrVII_A_nidulans_FGSC_A4:1593290-1593340** | **AN1680** |
| **ChrVII_A_nidulans_FGSC_A4:4350731-4350781** | **AN2556** |
| **ChrV_A_nidulans_FGSC_A4:1599000-1599050** | **AN12035** |
| **ChrI_A_nidulans_FGSC_A4:847553-847603** | **AN10795** |
| **ChrVI_A_nidulans_FGSC_A4:3260150-3260200** | **AN2650** |
| **ChrI_A_nidulans_FGSC_A4:221066-221116** | **AN6429** |
| **ChrI_A_nidulans_FGSC_A4:221066-221116** | **AN6430** |
| **ChrVII_A_nidulans_FGSC_A4:3126594-3126644** | **AN11329** |
| **ChrVII_A_nidulans_FGSC_A4:3126594-3126644** | **AN2180** |
| **ChrVII_A_nidulans_FGSC_A4:2775788-2775838** | **AN2073** |
| **ChrII_A_nidulans_FGSC_A4:2084875-2084925** | **AN12094** |
| **ChrIV_A_nidulans_FGSC_A4:1811777-1811827** | **AN7518** |
| **ChrVI_A_nidulans_FGSC_A4:3279362-3279412** | **AN2644** |
| **ChrVI_A_nidulans_FGSC_A4:3279362-3279412** | **AN2643** |
| **ChrII_A_nidulans_FGSC_A4:3639335-3639385** | **AN10446** |
| **ChrI_A_nidulans_FGSC_A4:1856348-1856398** | **AN5901** |
| **ChrIV_A_nidulans_FGSC_A4:765186-765236** | **AN7178** |
| **ChrIV_A_nidulans_FGSC_A4:765186-765236** | **AN7179** |
| **ChrVIII_A_nidulans_FGSC_A4:2873218-2873268** | **AN0658** |
| **ChrVIII_A_nidulans_FGSC_A4:2873218-2873268** | **AN0657** |
| **ChrVI_A_nidulans_FGSC_A4:1503592-1503642** | **AN3201** |
| **ChrIV_A_nidulans_FGSC_A4:614305-614355** | **AN10916** |
| **ChrVII_A_nidulans_FGSC_A4:1710381-1710431** | **AN1720** |
| **ChrII_A_nidulans_FGSC_A4:1800303-1800353** | **AN10515** |
| **ChrIV_A_nidulans_FGSC_A4:401351-401401** | **AN7291** |
| **ChrVI_A_nidulans_FGSC_A4:2796965-2797015** | **AN2793** |
| **ChrIV_A_nidulans_FGSC_A4:2663223-2663273** | **AN7776** |
| **ChrV_A_nidulans_FGSC_A4:1493509-1493559** | **AN10651** |
| **ChrV_A_nidulans_FGSC_A4:1493509-1493559** | **AN5210** |
| **ChrV_A_nidulans_FGSC_A4:2407466-2407516** | **AN10696** |
| **ChrV_A_nidulans_FGSC_A4:699462-699512** | **AN8502** |
| **ChrIV_A_nidulans_FGSC_A4:1251665-1251715** | **AN7031** |
| **ChrVIII_A_nidulans_FGSC_A4:223773-223823** | **AN11210** |
| **ChrVIII_A_nidulans_FGSC_A4:223773-223823** | **AN9300** |
| **ChrVIII_A_nidulans_FGSC_A4:2026206-2026256** | **AN0933** |
| **ChrVIII_A_nidulans_FGSC_A4:2051586-2051636** | **AN0927** |
| **ChrVIII_A_nidulans_FGSC_A4:3746993-3747043** | **AN10075** |
| **ChrVIII_A_nidulans_FGSC_A4:4524390-4524440** | **AN0124** |
| **ChrVII_A_nidulans_FGSC_A4:1548779-1548829** | **AN1664** |
| **ChrVII_A_nidulans_FGSC_A4:3280679-3280729** | **AN2235** |
| **ChrVII_A_nidulans_FGSC_A4:4156829-4156879** | **AN2503** |
| **ChrVI_A_nidulans_FGSC_A4:304536-304586** | **AN11932** |
| **ChrVI_A_nidulans_FGSC_A4:2278323-2278373** | **AN2953** |
| **ChrVII_A_nidulans_FGSC_A4:3005343-3005393** | **AN2138** |
| **ChrVIII_A_nidulans_FGSC_A4:1890520-1890570** | **AN0981** |
| **ChrVII_A_nidulans_FGSC_A4:3215985-3216035** | **AN2212** |
| **ChrVII_A_nidulans_FGSC_A4:3215985-3216035** | **AN2213** |
| **ChrIII_A_nidulans_FGSC_A4:1314837-1314887** | **AN4688** |
| **ChrIII_A_nidulans_FGSC_A4:1314837-1314887** | **AN4687** |
| **ChrI_A_nidulans_FGSC_A4:3122602-3122652** | **AN6774** |
| **ChrVIII_A_nidulans_FGSC_A4:3278068-3278118** | **AN0525** |
| **ChrVI_A_nidulans_FGSC_A4:457844-457894** | **AN11929** |
| **ChrVI_A_nidulans_FGSC_A4:677361-677411** | **AN3451** |
| **ChrVI_A_nidulans_FGSC_A4:677361-677411** | **AN3450** |
| **ChrV_A_nidulans_FGSC_A4:174875-174925** | **AN8341** |
| **ChrV_A_nidulans_FGSC_A4:337985-338035** | **AN8391** |
| **ChrII_A_nidulans_FGSC_A4:263288-263338** | **AN7932** |
| **ChrII_A_nidulans_FGSC_A4:263288-263338** | **AN7933** |
| **ChrVII_A_nidulans_FGSC_A4:1989942-1989992** | **AN1819** |
| **ChrVI_A_nidulans_FGSC_A4:2037863-2037913** | **AN3038** |
| **ChrVII_A_nidulans_FGSC_A4:1916635-1916685** | **AN1797** |
| **ChrIV_A_nidulans_FGSC_A4:1148813-1148863** | **AN7063** |
| **ChrVIII_A_nidulans_FGSC_A4:2001104-2001154** | **AN0944** |
| **ChrV_A_nidulans_FGSC_A4:2050493-2050543** | **AN10701** |
| **ChrV_A_nidulans_FGSC_A4:2482526-2482576** | **AN10678** |
| **ChrV_A_nidulans_FGSC_A4:2482526-2482576** | **AN5535** |
| **ChrIII_A_nidulans_FGSC_A4:1006308-1006358** | **AN4784** |
| **ChrIV_A_nidulans_FGSC_A4:63013-63063** | **AN7402** |
| **ChrIV_A_nidulans_FGSC_A4:1213027-1213077** | **AN12071** |
| **ChrVII_A_nidulans_FGSC_A4:447207-447257** | **AN8916** |
| **ChrVII_A_nidulans_FGSC_A4:2003276-2003326** | **AN1826** |
| **ChrVII_A_nidulans_FGSC_A4:2231962-2232012** | **AN1912** |
| **ChrV_A_nidulans_FGSC_A4:1934093-1934143** | **AN10703** |
| **ChrII_A_nidulans_FGSC_A4:2241135-2241185** | **AN4047** |
| **ChrII_A_nidulans_FGSC_A4:4000324-4000374** | **AN3493** |
| **ChrVI_A_nidulans_FGSC_A4:1867320-1867370** | **AN3092** |
| **ChrV_A_nidulans_FGSC_A4:731517-731567** | **AN8511** |
| **ChrV_A_nidulans_FGSC_A4:731517-731567** | **AN8510** |
| **ChrII_A_nidulans_FGSC_A4:1028431-1028481** | **AN11595** |
| **ChrI_A_nidulans_FGSC_A4:1391241-1391291** | **AN6058** |
| **ChrI_A_nidulans_FGSC_A4:1391241-1391291** | **AN6059** |
| **ChrI_A_nidulans_FGSC_A4:464233-464283** | **AN10801** |
| **ChrI_A_nidulans_FGSC_A4:3011025-3011075** | **AN6733** |
| **ChrII_A_nidulans_FGSC_A4:684954-685004** | **AN8079** |
| **ChrII_A_nidulans_FGSC_A4:1549188-1549238** | **AN4274** |
| **ChrII_A_nidulans_FGSC_A4:3372901-3372951** | **AN3687** |
| **ChrII_A_nidulans_FGSC_A4:3492536-3492586** | **AN3649** |
| **ChrIV_A_nidulans_FGSC_A4:296098-296148** | **AN7327** |
| **ChrIV_A_nidulans_FGSC_A4:1702507-1702557** | **AN7489** |
| **ChrVIII_A_nidulans_FGSC_A4:473209-473259** | **AN9384** |
| **ChrVII_A_nidulans_FGSC_A4:2840977-2841027** | **AN2094** |
| **ChrV_A_nidulans_FGSC_A4:1239522-1239572** | **AN5130** |
| **ChrV_A_nidulans_FGSC_A4:2410852-2410902** | **AN10693** |
| **ChrIII_A_nidulans_FGSC_A4:962538-962588** | **AN4797** |
| **ChrVIII_A_nidulans_FGSC_A4:1260257-1260307** | **AN1184** |
| **ChrVIII_A_nidulans_FGSC_A4:3765603-3765653** | **AN0370** |
| **ChrVI_A_nidulans_FGSC_A4:2186038-2186088** | **AN2981** |
| **ChrIV_A_nidulans_FGSC_A4:2095667-2095717** | **AN7599** |
| **ChrIV_A_nidulans_FGSC_A4:2145170-2145220** | **AN7614** |
| **ChrV_A_nidulans_FGSC_A4:484082-484132** | **AN8437** |
| **ChrV_A_nidulans_FGSC_A4:770284-770334** | **AN8529** |
| **ChrI_A_nidulans_FGSC_A4:1303935-1303985** | **AN6087** |
| **ChrVIII_A_nidulans_FGSC_A4:782600-782650** | **AN1341** |
| **ChrVIII_A_nidulans_FGSC_A4:782600-782650** | **AN1342** |
| **ChrIII_A_nidulans_FGSC_A4:2053845-2053895** | **AN11438** |
| **ChrVIII_A_nidulans_FGSC_A4:2771434-2771484** | **AN0696** |
| **ChrVII_A_nidulans_FGSC_A4:3787214-3787264** | **AN2393** |
| **ChrIII_A_nidulans_FGSC_A4:2333320-2333370** | **AN12154** |
| **ChrIV_A_nidulans_FGSC_A4:402581-402631** | **AN12245** |
| **ChrIV_A_nidulans_FGSC_A4:2490747-2490797** | **AN7726** |
| **ChrIV_A_nidulans_FGSC_A4:2627754-2627804** | **AN12084** |
| **ChrI_A_nidulans_FGSC_A4:1868121-1868171** | **AN5898** |
| **ChrI_A_nidulans_FGSC_A4:2089324-2089374** | **AN5836** |
| **ChrI_A_nidulans_FGSC_A4:2562928-2562978** | **AN6591** |
| **ChrI_A_nidulans_FGSC_A4:2562928-2562978** | **AN6590** |
| **ChrI_A_nidulans_FGSC_A4:3240115-3240165** | **AN6805** |
| **ChrI_A_nidulans_FGSC_A4:3671995-3672045** | **AN6950** |
| **ChrVIII_A_nidulans_FGSC_A4:656140-656190** | **AN1392** |
| **ChrVI_A_nidulans_FGSC_A4:492618-492668** | **AN9081** |
| **ChrVI_A_nidulans_FGSC_A4:2041599-2041649** | **AN3037** |
| **ChrVI_A_nidulans_FGSC_A4:2948610-2948660** | **AN2742** |
| **ChrI_A_nidulans_FGSC_A4:3473535-3473585** | **AN6886** |
| **ChrVIII_A_nidulans_FGSC_A4:729259-729309** | **AN1367** |
| **ChrIII_A_nidulans_FGSC_A4:2355890-2355940** | **AN10550** |
| **ChrI_A_nidulans_FGSC_A4:942850-942900** | **AN12053** |
| **ChrVII_A_nidulans_FGSC_A4:2905142-2905192** | **AN2114** |
| **ChrV_A_nidulans_FGSC_A4:463040-463090** | **AN8430** |
| **ChrIII_A_nidulans_FGSC_A4:379844-379894** | **AN11772** |
| **ChrIII_A_nidulans_FGSC_A4:379844-379894** | **AN4983** |
| **ChrII_A_nidulans_FGSC_A4:259343-259393** | **AN11030** |
| **ChrIV_A_nidulans_FGSC_A4:669612-669662** | **AN7211** |
| **ChrIV_A_nidulans_FGSC_A4:669612-669662** | **AN10907** |
| **ChrIII_A_nidulans_FGSC_A4:978018-978068** | **AN4792** |
| **ChrIII_A_nidulans_FGSC_A4:978018-978068** | **AN4791** |
| **ChrII_A_nidulans_FGSC_A4:1648151-1648201** | **AN4239** |
| **ChrI_A_nidulans_FGSC_A4:466509-466559** | **AN6342** |
| **ChrVIII_A_nidulans_FGSC_A4:1129280-1129330** | **AN1228** |
| **ChrVIII_A_nidulans_FGSC_A4:1147998-1148048** | **AN1221** |
| **ChrVII_A_nidulans_FGSC_A4:2685201-2685251** | **AN2043** |
| **ChrVII_A_nidulans_FGSC_A4:2685201-2685251** | **AN2044** |
| **ChrVI_A_nidulans_FGSC_A4:544576-544626** | **AN9066** |
| **ChrVI_A_nidulans_FGSC_A4:544576-544626** | **AN9065** |
| **ChrVI_A_nidulans_FGSC_A4:713916-713966** | **AN10411** |
| **ChrVI_A_nidulans_FGSC_A4:2111357-2111407** | **AN3010** |
| **ChrV_A_nidulans_FGSC_A4:1761358-1761408** | **AN5768** |
| **ChrV_A_nidulans_FGSC_A4:3004564-3004614** | **AN5377** |
| **ChrIII_A_nidulans_FGSC_A4:738160-738210** | **AN4863** |
| **ChrIII_A_nidulans_FGSC_A4:738160-738210** | **AN4862** |
| **ChrI_A_nidulans_FGSC_A4:413159-413209** | **AN6362** |
| **ChrVIII_A_nidulans_FGSC_A4:2592383-2592433** | **AN0748** |
| **ChrII_A_nidulans_FGSC_A4:601010-601060** | **AN8049** |
| **ChrII_A_nidulans_FGSC_A4:8449-8499** | **AN7851** |
| **ChrII_A_nidulans_FGSC_A4:8449-8499** | **AN7850** |
| **ChrII_A_nidulans_FGSC_A4:286044-286094** | **AN7942** |
| **ChrII_A_nidulans_FGSC_A4:782337-782387** | **AN11942** |
| **ChrII_A_nidulans_FGSC_A4:782337-782387** | **AN8110** |
| **ChrIV_A_nidulans_FGSC_A4:1021283-1021333** | **AN10892** |
| **ChrVII_A_nidulans_FGSC_A4:3580143-3580193** | **AN2330** |
| **ChrVI_A_nidulans_FGSC_A4:2987363-2987413** | **AN2732** |
| **ChrIII_A_nidulans_FGSC_A4:3425517-3425567** | **AN8591** |
| **ChrII_A_nidulans_FGSC_A4:262589-262639** | **AN7933** |
| **ChrII_A_nidulans_FGSC_A4:3120875-3120925** | **AN10457** |
| **ChrVI_A_nidulans_FGSC_A4:430629-430679** | **AN9100** |
| **ChrVI_A_nidulans_FGSC_A4:2894921-2894971** | **AN10339** |
| **ChrIII_A_nidulans_FGSC_A4:965602-965652** | **AN4795** |
| **ChrII_A_nidulans_FGSC_A4:1328898-1328948** | **AN8266** |
| **ChrII_A_nidulans_FGSC_A4:1328898-1328948** | **AN8267** |
| **ChrVIII_A_nidulans_FGSC_A4:4834349-4834399** | **AN0023** |
| **ChrVIII_A_nidulans_FGSC_A4:4834349-4834399** | **AN10005** |
| **ChrII_A_nidulans_FGSC_A4:1760121-1760171** | **AN10517** |
| **ChrII_A_nidulans_FGSC_A4:1760121-1760171** | **AN4203** |
| **ChrI_A_nidulans_FGSC_A4:2744569-2744619** | **AN6653** |
| **ChrII_A_nidulans_FGSC_A4:1673360-1673410** | **AN4233** |
| **ChrII_A_nidulans_FGSC_A4:1673360-1673410** | **AN4232** |
| **ChrVI_A_nidulans_FGSC_A4:2412336-2412386** | **AN2914** |
| **ChrV_A_nidulans_FGSC_A4:1269080-1269130** | **AN10645** |
| **ChrIII_A_nidulans_FGSC_A4:487381-487431** | **AN4944** |
| **ChrII_A_nidulans_FGSC_A4:2335649-2335699** | **AN4014** |
| **ChrII_A_nidulans_FGSC_A4:2335649-2335699** | **AN4015** |
| **ChrI_A_nidulans_FGSC_A4:571195-571245** | **AN11853** |
| **ChrI_A_nidulans_FGSC_A4:571195-571245** | **AN6315** |
| **ChrVIII_A_nidulans_FGSC_A4:974862-974912** | **AN1276** |
| **ChrVIII_A_nidulans_FGSC_A4:974862-974912** | **AN1277** |
| **ChrII_A_nidulans_FGSC_A4:185769-185819** | **AN7903** |
| **ChrII_A_nidulans_FGSC_A4:185769-185819** | **AN7902** |
| **ChrIV_A_nidulans_FGSC_A4:1944979-1945029** | **AN7554** |
| **ChrVII_A_nidulans_FGSC_A4:1730372-1730422** | **AN10232** |
| **ChrVII_A_nidulans_FGSC_A4:2266738-2266788** | **AN1921** |
| **ChrVII_A_nidulans_FGSC_A4:2266738-2266788** | **AN1922** |
| **ChrVI_A_nidulans_FGSC_A4:2592213-2592263** | **AN2855** |
| **ChrI_A_nidulans_FGSC_A4:1966631-1966681** | **AN5868** |
| **ChrVII_A_nidulans_FGSC_A4:3204064-3204114** | **AN10274** |
| **ChrVIII_A_nidulans_FGSC_A4:2535848-2535898** | **AN0765** |
| **ChrVII_A_nidulans_FGSC_A4:3571030-3571080** | **AN2327** |
| **ChrIV_A_nidulans_FGSC_A4:2598283-2598333** | **AN7752** |
| **ChrIV_A_nidulans_FGSC_A4:2598283-2598333** | **AN7753** |
| **ChrIII_A_nidulans_FGSC_A4:2612580-2612630** | **AN8845** |
| **ChrIII_A_nidulans_FGSC_A4:2612580-2612630** | **AN8844** |
| **ChrIII_A_nidulans_FGSC_A4:2707281-2707331** | **AN12435** |
| **ChrIII_A_nidulans_FGSC_A4:2707281-2707331** | **AN8819** |
| **ChrII_A_nidulans_FGSC_A4:1170741-1170791** | **AN8215** |
| **ChrII_A_nidulans_FGSC_A4:3904299-3904349** | **AN3523** |
| **ChrIV_A_nidulans_FGSC_A4:48331-48381** | **AN7408** |
| **ChrIV_A_nidulans_FGSC_A4:117699-117749** | **AN7386** |
| **ChrI_A_nidulans_FGSC_A4:488058-488108** | **AN10808** |
| **ChrIII_A_nidulans_FGSC_A4:2018787-2018837** | **AN4450** |
| **ChrIII_A_nidulans_FGSC_A4:2018787-2018837** | **AN4448** |
| **ChrVI_A_nidulans_FGSC_A4:699338-699388** | **AN10413** |
| **ChrVI_A_nidulans_FGSC_A4:1985097-1985147** | **AN3054** |
| **ChrIII_A_nidulans_FGSC_A4:327352-327402** | **AN11773** |
| **ChrVII_A_nidulans_FGSC_A4:1122602-1122652** | **AN1546** |
| **ChrII_A_nidulans_FGSC_A4:2151396-2151446** | **AN4082** |
| **ChrII_A_nidulans_FGSC_A4:3956809-3956859** | **AN3505** |
| **ChrIV_A_nidulans_FGSC_A4:890955-891005** | **AN7135** |
| **ChrIV_A_nidulans_FGSC_A4:2325897-2325947** | **AN7669** |
| **ChrIII_A_nidulans_FGSC_A4:2303987-2304037** | **AN4353** |
| **ChrVIII_A_nidulans_FGSC_A4:1539395-1539445** | **AN1094** |
| **ChrVIII_A_nidulans_FGSC_A4:3172769-3172819** | **AN0560** |
| **ChrVIII_A_nidulans_FGSC_A4:3172769-3172819** | **AN0559** |
| **ChrV_A_nidulans_FGSC_A4:1185176-1185226** | **AN5109** |
| **ChrV_A_nidulans_FGSC_A4:1185176-1185226** | **AN5110** |
| **ChrIII_A_nidulans_FGSC_A4:141442-141492** | **AN5055** |
| **ChrII_A_nidulans_FGSC_A4:2731464-2731514** | **AN3901** |
| **ChrIV_A_nidulans_FGSC_A4:1138677-1138727** | **AN7067** |
| **ChrIV_A_nidulans_FGSC_A4:2430904-2430954** | **AN11001** |
| **ChrI_A_nidulans_FGSC_A4:535578-535628** | **AN6324** |
| **ChrI_A_nidulans_FGSC_A4:1698162-1698212** | **AN5950** |
| **ChrVIII_A_nidulans_FGSC_A4:99457-99507** | **AN9261** |
| **ChrVIII_A_nidulans_FGSC_A4:3902337-3902387** | **AN0322** |
| **ChrVI_A_nidulans_FGSC_A4:2675108-2675158** | **AN2827** |
| **ChrV_A_nidulans_FGSC_A4:1818354-1818404** | **AN5748** |
| **ChrIV_A_nidulans_FGSC_A4:2695012-2695062** | **AN7787** |
| **ChrIV_A_nidulans_FGSC_A4:2695012-2695062** | **AN7788** |
| **ChrI_A_nidulans_FGSC_A4:956906-956956** | **AN6196** |
| **ChrI_A_nidulans_FGSC_A4:2050491-2050541** | **AN5848** |
| **ChrI_A_nidulans_FGSC_A4:2050491-2050541** | **AN5847** |
| **ChrVIII_A_nidulans_FGSC_A4:178044-178094** | **AN9286** |
| **ChrVIII_A_nidulans_FGSC_A4:178044-178094** | **AN9287** |
| **ChrI_A_nidulans_FGSC_A4:451530-451580** | **AN6347** |
| **ChrII_A_nidulans_FGSC_A4:737322-737372** | **AN11035** |
| **ChrII_A_nidulans_FGSC_A4:1123742-1123792** | **AN8204** |
| **ChrII_A_nidulans_FGSC_A4:1123742-1123792** | **AN8203** |
| **ChrII_A_nidulans_FGSC_A4:1385744-1385794** | **AN8286** |
| **ChrII_A_nidulans_FGSC_A4:1853008-1853058** | **AN4180** |
| **ChrII_A_nidulans_FGSC_A4:1853008-1853058** | **AN10512** |
| **ChrII_A_nidulans_FGSC_A4:3677692-3677742** | **AN11715** |
| **ChrVIII_A_nidulans_FGSC_A4:2904320-2904370** | **AN0646** |
| **ChrV_A_nidulans_FGSC_A4:462215-462265** | **AN8431** |
| **ChrV_A_nidulans_FGSC_A4:3255387-3255437** | **AN10666** |
| **ChrII_A_nidulans_FGSC_A4:2925886-2925936** | **AN10477** |
| **ChrIII_A_nidulans_FGSC_A4:1476849-1476899** | **AN4631** |
| **ChrII_A_nidulans_FGSC_A4:3423813-3423863** | **AN3669** |
| **ChrVIII_A_nidulans_FGSC_A4:428136-428186** | **AN9370** |
| **ChrI_A_nidulans_FGSC_A4:580321-580371** | **AN6312** |
| **ChrVI_A_nidulans_FGSC_A4:1562798-1562848** | **AN3181** |
| **ChrII_A_nidulans_FGSC_A4:2872375-2872425** | **AN3851** |
| **ChrVIII_A_nidulans_FGSC_A4:2799630-2799680** | **AN0684** |
| **ChrVI_A_nidulans_FGSC_A4:2306593-2306643** | **AN10360** |
| **ChrV_A_nidulans_FGSC_A4:306764-306814** | **AN11871** |
| **ChrV_A_nidulans_FGSC_A4:467039-467089** | **AN8432** |
| **ChrII_A_nidulans_FGSC_A4:2379512-2379562** | **AN4000** |
| **ChrVIII_A_nidulans_FGSC_A4:1014085-1014135** | **AN10169** |
| **ChrV_A_nidulans_FGSC_A4:2923323-2923373** | **AN11808** |
| **ChrI_A_nidulans_FGSC_A4:1151861-1151911** | **AN10787** |
| **ChrI_A_nidulans_FGSC_A4:1151861-1151911** | **AN6130** |
| **ChrVI_A_nidulans_FGSC_A4:1910763-1910813** | **AN3079** |
| **ChrVII_A_nidulans_FGSC_A4:1601681-1601731** | **AN1682** |
| **ChrII_A_nidulans_FGSC_A4:446718-446768** | **AN12402** |
| **ChrII_A_nidulans_FGSC_A4:446718-446768** | **AN7999** |
| **ChrII_A_nidulans_FGSC_A4:2055300-2055350** | **AN4113** |
| **ChrII_A_nidulans_FGSC_A4:3545159-3545209** | **AN3629** |
| **ChrII_A_nidulans_FGSC_A4:3545159-3545209** | **AN11718** |
| **ChrI_A_nidulans_FGSC_A4:120250-120300** | **AN6454** |
| **ChrI_A_nidulans_FGSC_A4:872324-872374** | **AN11245** |
| **ChrVI_A_nidulans_FGSC_A4:242612-242662** | **AN9161** |
| **ChrVI_A_nidulans_FGSC_A4:242612-242662** | **AN11187** |
| **ChrVI_A_nidulans_FGSC_A4:1117557-1117607** | **AN3321** |
| **ChrVI_A_nidulans_FGSC_A4:1973327-1973377** | **AN3060** |
| **ChrV_A_nidulans_FGSC_A4:3193628-3193678** | **AN10660** |
| **ChrVIII_A_nidulans_FGSC_A4:2340247-2340297** | **AN0833** |
| **ChrVII_A_nidulans_FGSC_A4:4440045-4440095** | **AN11965** |
| **ChrVII_A_nidulans_FGSC_A4:4440045-4440095** | **AN2584** |
| **ChrIII_A_nidulans_FGSC_A4:1787161-1787211** | **AN4519** |
| **ChrI_A_nidulans_FGSC_A4:2420649-2420699** | **AN10830** |
| **ChrVII_A_nidulans_FGSC_A4:990902-990952** | **AN1501** |
| **ChrV_A_nidulans_FGSC_A4:2882644-2882694** | **AN5415** |
| **ChrV_A_nidulans_FGSC_A4:2882644-2882694** | **AN5416** |
| **ChrIII_A_nidulans_FGSC_A4:2198259-2198309** | **AN4383** |
| **ChrIII_A_nidulans_FGSC_A4:2198259-2198309** | **AN4384** |
| **ChrIV_A_nidulans_FGSC_A4:2610858-2610908** | **AN7757** |
| **ChrV_A_nidulans_FGSC_A4:2047141-2047191** | **AN5663** |
| **ChrIII_A_nidulans_FGSC_A4:3126015-3126065** | **AN8685** |
| **ChrIII_A_nidulans_FGSC_A4:3126015-3126065** | **AN8684** |
| **ChrII_A_nidulans_FGSC_A4:1626662-1626712** | **AN4247** |
| **ChrIV_A_nidulans_FGSC_A4:1830705-1830755** | **AN7524** |
| **ChrIV_A_nidulans_FGSC_A4:1830705-1830755** | **AN11752** |
| **ChrI_A_nidulans_FGSC_A4:3701887-3701937** | **AN9440** |
| **ChrIII_A_nidulans_FGSC_A4:3237109-3237159** | **AN8657** |
| **ChrIII_A_nidulans_FGSC_A4:3237109-3237159** | **AN8656** |
| **ChrVII_A_nidulans_FGSC_A4:2989723-2989773** | **AN2135** |
| **ChrV_A_nidulans_FGSC_A4:1813265-1813315** | **AN5749** |
| **ChrVI_A_nidulans_FGSC_A4:2404986-2405036** | **AN2916** |
| **ChrVII_A_nidulans_FGSC_A4:817288-817338** | **AN1450** |
| **ChrVI_A_nidulans_FGSC_A4:1550036-1550086** | **AN3186** |
| **ChrVI_A_nidulans_FGSC_A4:1550036-1550086** | **AN3185** |
| **ChrVI_A_nidulans_FGSC_A4:1508611-1508661** | **AN3199** |
| **ChrVI_A_nidulans_FGSC_A4:1508611-1508661** | **AN10384** |
| **ChrI_A_nidulans_FGSC_A4:330655-330705** | **AN6390** |
| **ChrI_A_nidulans_FGSC_A4:3595411-3595461** | **AN10866** |
| **ChrI_A_nidulans_FGSC_A4:788936-788986** | **AN6239** |
| **ChrI_A_nidulans_FGSC_A4:788936-788986** | **AN6240** |
| **ChrVIII_A_nidulans_FGSC_A4:1996206-1996256** | **AN10135** |
| **ChrVIII_A_nidulans_FGSC_A4:3357920-3357970** | **AN0501** |
| **ChrVI_A_nidulans_FGSC_A4:1475598-1475648** | **AN3209** |
| **ChrII_A_nidulans_FGSC_A4:3576840-3576890** | **AN3619** |
| **ChrI_A_nidulans_FGSC_A4:3222451-3222501** | **AN6802** |
| **ChrI_A_nidulans_FGSC_A4:3222451-3222501** | **AN6803** |
| **ChrVII_A_nidulans_FGSC_A4:843314-843364** | **AN1459** |
| **ChrVII_A_nidulans_FGSC_A4:1070084-1070134** | **AN1528** |
| **ChrVI_A_nidulans_FGSC_A4:366702-366752** | **AN9126** |
| **ChrVI_A_nidulans_FGSC_A4:2464735-2464785** | **AN2897** |
| **ChrV_A_nidulans_FGSC_A4:1258452-1258502** | **AN10634** |
| **ChrV_A_nidulans_FGSC_A4:1258452-1258502** | **AN5135** |
| **ChrVII_A_nidulans_FGSC_A4:2314969-2315019** | **AN1937** |
| **ChrV_A_nidulans_FGSC_A4:2833839-2833889** | **AN5432** |
| **ChrV_A_nidulans_FGSC_A4:2833839-2833889** | **AN5433** |
| **ChrVII_A_nidulans_FGSC_A4:1743515-1743565** | **AN1733** |
| **ChrII_A_nidulans_FGSC_A4:2665868-2665918** | **AN3913** |
| **ChrI_A_nidulans_FGSC_A4:1906942-1906992** | **AN5886** |
| **ChrVIII_A_nidulans_FGSC_A4:3109107-3109157** | **AN0578** |
| **ChrVII_A_nidulans_FGSC_A4:2070615-2070665** | **AN1852** |
| **ChrVI_A_nidulans_FGSC_A4:2572420-2572470** | **AN2864** |
| **ChrV_A_nidulans_FGSC_A4:8759-8809** | **AN12418** |
| **ChrI_A_nidulans_FGSC_A4:654456-654506** | **AN6286** |
| **ChrI_A_nidulans_FGSC_A4:1443238-1443288** | **AN6037** |
| **ChrI_A_nidulans_FGSC_A4:1443238-1443288** | **AN6036** |
| **ChrVIII_A_nidulans_FGSC_A4:3705526-3705576** | **AN0391** |
| **ChrIV_A_nidulans_FGSC_A4:2437233-2437283** | **AN7705** |
| **ChrIV_A_nidulans_FGSC_A4:2437233-2437283** | **AN7704** |
| **ChrI_A_nidulans_FGSC_A4:2062340-2062390** | **AN5842** |
| **ChrVIII_A_nidulans_FGSC_A4:4841801-4841851** | **AN0020** |
| **ChrI_A_nidulans_FGSC_A4:1258073-1258123** | **AN10775** |
| **ChrVIII_A_nidulans_FGSC_A4:2560424-2560474** | **AN0756** |
| **ChrVII_A_nidulans_FGSC_A4:1856281-1856331** | **AN1778** |
| **ChrII_A_nidulans_FGSC_A4:1925699-1925749** | **AN4155** |
| **ChrII_A_nidulans_FGSC_A4:1925699-1925749** | **AN4156** |
| **ChrVI_A_nidulans_FGSC_A4:1457178-1457228** | **AN3213** |
| **ChrIII_A_nidulans_FGSC_A4:668991-669041** | **AN10609** |
| **ChrIII_A_nidulans_FGSC_A4:668991-669041** | **AN4886** |
| **ChrII_A_nidulans_FGSC_A4:2058830-2058880** | **AN4110** |
| **ChrI_A_nidulans_FGSC_A4:1937102-1937152** | **AN5877** |
| **ChrI_A_nidulans_FGSC_A4:1937102-1937152** | **AN5878** |
| **ChrV_A_nidulans_FGSC_A4:2982350-2982400** | **AN5385** |
| **ChrIV_A_nidulans_FGSC_A4:885610-885660** | **AN10903** |
| **ChrVIII_A_nidulans_FGSC_A4:2521109-2521159** | **AN0771** |
| **ChrIV_A_nidulans_FGSC_A4:2068882-2068932** | **AN10969** |
| **ChrIII_A_nidulans_FGSC_A4:3018071-3018121** | **AN8723** |
| **ChrVII_A_nidulans_FGSC_A4:2488682-2488732** | **AN1993** |
| **ChrVI_A_nidulans_FGSC_A4:157555-157605** | **AN9189** |
| **ChrII_A_nidulans_FGSC_A4:2643065-2643115** | **AN3922** |
| **ChrII_A_nidulans_FGSC_A4:2643065-2643115** | **AN3921** |
| **ChrIII_A_nidulans_FGSC_A4:581850-581900** | **AN4912** |
| **ChrII_A_nidulans_FGSC_A4:1921249-1921299** | **AN4157** |
| **ChrIV_A_nidulans_FGSC_A4:346042-346092** | **AN7309** |
| **ChrIV_A_nidulans_FGSC_A4:2862787-2862837** | **AN7845** |
| **ChrI_A_nidulans_FGSC_A4:2296698-2296748** | **AN10826** |
| **ChrVIII_A_nidulans_FGSC_A4:3597921-3597971** | **AN0423** |
| **ChrVII_A_nidulans_FGSC_A4:3429823-3429873** | **AN2282** |
| **ChrVI_A_nidulans_FGSC_A4:2468323-2468373** | **AN2896** |
| **ChrII_A_nidulans_FGSC_A4:2462992-2463042** | **AN3972** |
| **ChrI_A_nidulans_FGSC_A4:155250-155300** | **AN6445** |
| **ChrI_A_nidulans_FGSC_A4:126082-126132** | **AN6452** |
| **ChrVIII_A_nidulans_FGSC_A4:1759725-1759775** | **AN1026** |
| **ChrVII_A_nidulans_FGSC_A4:2746057-2746107** | **AN2066** |
| **ChrI_A_nidulans_FGSC_A4:1511599-1511649** | **AN6013** |
| **ChrI_A_nidulans_FGSC_A4:1511599-1511649** | **AN6014** |
| **ChrII_A_nidulans_FGSC_A4:1372485-1372535** | **AN8281** |
| **ChrII_A_nidulans_FGSC_A4:1998940-1998990** | **AN4129** |
| **ChrIV_A_nidulans_FGSC_A4:1703997-1704047** | **AN7488** |
| **ChrIV_A_nidulans_FGSC_A4:2024364-2024414** | **AN10972** |
| **ChrIV_A_nidulans_FGSC_A4:2236237-2236287** | **AN7642** |
| **ChrI_A_nidulans_FGSC_A4:1930643-1930693** | **AN5882** |
| **ChrVIII_A_nidulans_FGSC_A4:4775301-4775351** | **AN0040** |
| **ChrVIII_A_nidulans_FGSC_A4:4775301-4775351** | **AN0041** |
| **ChrVII_A_nidulans_FGSC_A4:117276-117326** | **AN9019** |
| **ChrVII_A_nidulans_FGSC_A4:923069-923119** | **AN1482** |
| **ChrVII_A_nidulans_FGSC_A4:923069-923119** | **AN1481** |
| **ChrVII_A_nidulans_FGSC_A4:2163844-2163894** | **AN1888** |
| **ChrVII_A_nidulans_FGSC_A4:3546243-3546293** | **AN2319** |
| **ChrV_A_nidulans_FGSC_A4:368121-368171** | **AN8400** |
| **ChrVIII_A_nidulans_FGSC_A4:762525-762575** | **AN1352** |
| **ChrVIII_A_nidulans_FGSC_A4:762525-762575** | **AN1353** |
| **ChrVIII_A_nidulans_FGSC_A4:3948198-3948248** | **AN0311** |
| **ChrIII_A_nidulans_FGSC_A4:3035914-3035964** | **AN8717** |
| **ChrI_A_nidulans_FGSC_A4:3032561-3032611** | **AN6740** |
| **ChrVII_A_nidulans_FGSC_A4:772969-773019** | **AN1435** |
| **ChrIII_A_nidulans_FGSC_A4:528736-528786** | **AN4927** |
| **ChrIII_A_nidulans_FGSC_A4:528736-528786** | **AN4928** |
| **ChrIV_A_nidulans_FGSC_A4:2315769-2315819** | **AN10982** |
| **ChrIV_A_nidulans_FGSC_A4:2315769-2315819** | **AN7665** |
| **ChrVII_A_nidulans_FGSC_A4:2322234-2322284** | **AN1939** |
| **ChrI_A_nidulans_FGSC_A4:3228899-3228949** | **AN11980** |
| **ChrI_A_nidulans_FGSC_A4:3228899-3228949** | **AN6804** |
| **ChrV_A_nidulans_FGSC_A4:2963874-2963924** | **AN10672** |
| **ChrIII_A_nidulans_FGSC_A4:3259387-3259437** | **AN8649** |
| **ChrIV_A_nidulans_FGSC_A4:2541821-2541871** | **AN7737** |
| **ChrI_A_nidulans_FGSC_A4:680913-680963** | **AN6277** |
| **ChrI_A_nidulans_FGSC_A4:1122971-1123021** | **AN6136** |
| **ChrVII_A_nidulans_FGSC_A4:1479628-1479678** | **AN1649** |
| **ChrVII_A_nidulans_FGSC_A4:1479628-1479678** | **AN1650** |
| **ChrV_A_nidulans_FGSC_A4:2416084-2416134** | **AN5558** |
| **ChrV_A_nidulans_FGSC_A4:3226527-3226577** | **AN5297** |
| **ChrVIII_A_nidulans_FGSC_A4:2543905-2543955** | **AN0762** |
| **ChrVIII_A_nidulans_FGSC_A4:2543905-2543955** | **AN0763** |
| **ChrIV_A_nidulans_FGSC_A4:306022-306072** | **AN7324** |
| **ChrV_A_nidulans_FGSC_A4:2826076-2826126** | **AN5435** |
| **ChrII_A_nidulans_FGSC_A4:3975496-3975546** | **AN3500** |
| **ChrVI_A_nidulans_FGSC_A4:1198140-1198190** | **AN3300** |
| **ChrVI_A_nidulans_FGSC_A4:1648810-1648860** | **AN3152** |
| **ChrII_A_nidulans_FGSC_A4:2278530-2278580** | **AN4035** |
| **ChrIII_A_nidulans_FGSC_A4:15654-15704** | **AN5089** |
| **ChrII_A_nidulans_FGSC_A4:255644-255694** | **AN7928** |
| **ChrII_A_nidulans_FGSC_A4:1004604-1004654** | **AN8172** |
| **ChrIV_A_nidulans_FGSC_A4:2860032-2860082** | **AN11027** |
| **ChrI_A_nidulans_FGSC_A4:1667512-1667562** | **AN5962** |
| **ChrI_A_nidulans_FGSC_A4:2317076-2317126** | **AN6505** |
| **ChrVIII_A_nidulans_FGSC_A4:955005-955055** | **AN10175** |
| **ChrVIII_A_nidulans_FGSC_A4:2557173-2557223** | **AN0757** |
| **ChrVII_A_nidulans_FGSC_A4:1687081-1687131** | **AN1711** |
| **ChrVII_A_nidulans_FGSC_A4:4011820-4011870** | **AN12235** |
| **ChrVI_A_nidulans_FGSC_A4:640767-640817** | **AN3463** |
| **ChrVI_A_nidulans_FGSC_A4:640767-640817** | **AN3462** |
| **ChrV_A_nidulans_FGSC_A4:1226373-1226423** | **AN5126** |
| **ChrV_A_nidulans_FGSC_A4:2549500-2549550** | **AN10676** |
| **ChrVII_A_nidulans_FGSC_A4:4153066-4153116** | **AN2502** |
| **ChrVII_A_nidulans_FGSC_A4:3133050-3133100** | **AN12199** |
| **ChrVIII_A_nidulans_FGSC_A4:3830249-3830299** | **AN0351** |
| **ChrV_A_nidulans_FGSC_A4:2123400-2123450** | **AN11487** |
| **ChrV_A_nidulans_FGSC_A4:2123400-2123450** | **AN5640** |
| **ChrVI_A_nidulans_FGSC_A4:283269-283319** | **AN11185** |
| **ChrIII_A_nidulans_FGSC_A4:2881163-2881213** | **AN8768** |
| **ChrIII_A_nidulans_FGSC_A4:2881163-2881213** | **AN8767** |
| **ChrII_A_nidulans_FGSC_A4:3272229-3272279** | **AN3724** |
| **ChrII_A_nidulans_FGSC_A4:3272229-3272279** | **AN10441** |
| **ChrVIII_A_nidulans_FGSC_A4:850395-850445** | **AN1317** |
| **ChrVIII_A_nidulans_FGSC_A4:4319656-4319706** | **AN0197** |
| **ChrVII_A_nidulans_FGSC_A4:393124-393174** | **AN8931** |
| **ChrVII_A_nidulans_FGSC_A4:3363033-3363083** | **AN2260** |
| **ChrVII_A_nidulans_FGSC_A4:3363033-3363083** | **AN2261** |
| **ChrVII_A_nidulans_FGSC_A4:4507306-4507356** | **AN2606** |
| **ChrVI_A_nidulans_FGSC_A4:3232669-3232719** | **AN2660** |
| **ChrV_A_nidulans_FGSC_A4:662582-662632** | **AN11878** |
| **ChrII_A_nidulans_FGSC_A4:2627261-2627311** | **AN3927** |
| **ChrV_A_nidulans_FGSC_A4:391015-391065** | **AN8410** |
| **ChrV_A_nidulans_FGSC_A4:2229672-2229722** | **AN5608** |
| **ChrIII_A_nidulans_FGSC_A4:59496-59546** | **AN12280** |
| **ChrVII_A_nidulans_FGSC_A4:1591827-1591877** | **AN1679** |
| **ChrIV_A_nidulans_FGSC_A4:2171476-2171526** | **AN10987** |
| **ChrVIII_A_nidulans_FGSC_A4:1619575-1619625** | **AN1070** |
| **ChrVII_A_nidulans_FGSC_A4:1959092-1959142** | **AN1809** |
| **ChrVI_A_nidulans_FGSC_A4:1559589-1559639** | **AN3182** |
| **ChrII_A_nidulans_FGSC_A4:3613956-3614006** | **AN10430** |
| **ChrI_A_nidulans_FGSC_A4:1824443-1824493** | **AN5916** |
| **ChrVIII_A_nidulans_FGSC_A4:2368600-2368650** | **AN0822** |
| **ChrIII_A_nidulans_FGSC_A4:2575420-2575470** | **AN8860** |
| **ChrII_A_nidulans_FGSC_A4:2392636-2392686** | **AN3996** |
| **ChrI_A_nidulans_FGSC_A4:355826-355876** | **AN9498** |
| **ChrI_A_nidulans_FGSC_A4:2056755-2056805** | **AN5843** |
| **ChrI_A_nidulans_FGSC_A4:2056755-2056805** | **AN5845** |
| **ChrVIII_A_nidulans_FGSC_A4:2083109-2083159** | **AN0916** |
| **ChrVIII_A_nidulans_FGSC_A4:4418266-4418316** | **AN11260** |
| **ChrVI_A_nidulans_FGSC_A4:2521005-2521055** | **AN2878** |
| **ChrVI_A_nidulans_FGSC_A4:3141945-3141995** | **AN2689** |
| **ChrI_A_nidulans_FGSC_A4:333075-333125** | **AN6389** |
| **ChrI_A_nidulans_FGSC_A4:333075-333125** | **AN6388** |
| **ChrI_A_nidulans_FGSC_A4:1549912-1549962** | **AN5999** |
| **ChrI_A_nidulans_FGSC_A4:3490671-3490721** | **AN6891** |
| **ChrI_A_nidulans_FGSC_A4:3490671-3490721** | **AN6890** |
| **ChrVIII_A_nidulans_FGSC_A4:3100472-3100522** | **AN0582** |
| **ChrIII_A_nidulans_FGSC_A4:1050585-1050635** | **AN4769** |
| **ChrII_A_nidulans_FGSC_A4:3421541-3421591** | **AN3670** |
| **ChrIII_A_nidulans_FGSC_A4:346699-346749** | **AN4993** |
| **ChrIII_A_nidulans_FGSC_A4:346699-346749** | **AN4992** |
| **ChrI_A_nidulans_FGSC_A4:2476917-2476967** | **AN6567** |
| **ChrIII_A_nidulans_FGSC_A4:1026117-1026167** | **AN4775** |
| **ChrIII_A_nidulans_FGSC_A4:1026117-1026167** | **AN4774** |
| **ChrIII_A_nidulans_FGSC_A4:1527876-1527926** | **AN4611** |
| **ChrII_A_nidulans_FGSC_A4:3087139-3087189** | **AN3776** |
| **ChrII_A_nidulans_FGSC_A4:3576288-3576338** | **AN3619** |
| **ChrII_A_nidulans_FGSC_A4:3624389-3624439** | **AN3608** |
| **ChrVII_A_nidulans_FGSC_A4:922303-922353** | **AN1482** |
| **ChrVI_A_nidulans_FGSC_A4:4787-4837** | **AN9234** |
| **ChrVI_A_nidulans_FGSC_A4:4787-4837** | **AN9235** |
| **ChrVI_A_nidulans_FGSC_A4:789282-789332** | **AN3417** |
| **ChrI_A_nidulans_FGSC_A4:1056393-1056443** | **AN6161** |
| **ChrVIII_A_nidulans_FGSC_A4:2642308-2642358** | **AN10123** |
| **ChrVI_A_nidulans_FGSC_A4:1133696-1133746** | **AN3318** |
| **ChrV_A_nidulans_FGSC_A4:1411793-1411843** | **AN5180** |
| **ChrII_A_nidulans_FGSC_A4:2365459-2365509** | **AN4005** |
| **ChrIV_A_nidulans_FGSC_A4:2128680-2128730** | **AN7608** |
| **ChrVIII_A_nidulans_FGSC_A4:525367-525417** | **AN9402** |
| **ChrVIII_A_nidulans_FGSC_A4:525367-525417** | **AN9403** |
| **ChrVII_A_nidulans_FGSC_A4:2609943-2609993** | **AN2023** |
| **ChrVII_A_nidulans_FGSC_A4:2609943-2609993** | **AN2022** |
| **ChrVII_A_nidulans_FGSC_A4:4421151-4421201** | **AN10321** |
| **ChrVI_A_nidulans_FGSC_A4:1748025-1748075** | **AN3125** |
| **ChrVI_A_nidulans_FGSC_A4:1748025-1748075** | **AN3124** |
| **ChrV_A_nidulans_FGSC_A4:1886995-1887045** | **AN5725** |
| **ChrIII_A_nidulans_FGSC_A4:2790361-2790411** | **AN8793** |
| **ChrII_A_nidulans_FGSC_A4:1593450-1593500** | **AN4260** |
| **ChrI_A_nidulans_FGSC_A4:1420839-1420889** | **AN6048** |
| **ChrV_A_nidulans_FGSC_A4:3208975-3209025** | **AN5304** |
| **ChrVII_A_nidulans_FGSC_A4:1960211-1960261** | **AN1811** |
| **ChrVII_A_nidulans_FGSC_A4:3261133-3261183** | **AN2227** |
| **ChrIV_A_nidulans_FGSC_A4:1771522-1771572** | **AN7508** |
| **ChrVIII_A_nidulans_FGSC_A4:4355299-4355349** | **AN0180** |
| **ChrVIII_A_nidulans_FGSC_A4:4355299-4355349** | **AN0181** |
| **ChrVI_A_nidulans_FGSC_A4:1299204-1299254** | **AN3263** |
| **ChrVI_A_nidulans_FGSC_A4:2342648-2342698** | **AN2936** |
| **ChrI_A_nidulans_FGSC_A4:3448358-3448408** | **AN6877** |
| **ChrVII_A_nidulans_FGSC_A4:1562993-1563043** | **AN1670** |
| **ChrII_A_nidulans_FGSC_A4:3017977-3018027** | **AN3797** |
| **ChrI_A_nidulans_FGSC_A4:705664-705714** | **AN6267** |
| **ChrI_A_nidulans_FGSC_A4:1461619-1461669** | **AN6027** |
| **ChrI_A_nidulans_FGSC_A4:2491154-2491204** | **AN6573** |
| **ChrI_A_nidulans_FGSC_A4:3526664-3526714** | **AN6905** |
| **ChrVII_A_nidulans_FGSC_A4:1654299-1654349** | **AN1698** |
| **ChrVI_A_nidulans_FGSC_A4:654926-654976** | **AN3459** |
| **ChrVI_A_nidulans_FGSC_A4:3074693-3074743** | **AN2709** |
| **ChrVI_A_nidulans_FGSC_A4:3084069-3084119** | **AN12252** |
| **ChrVI_A_nidulans_FGSC_A4:3124399-3124449** | **AN2695** |
| **ChrVII_A_nidulans_FGSC_A4:1908722-1908772** | **AN1794** |
| **ChrVIII_A_nidulans_FGSC_A4:4358235-4358285** | **AN0179** |
| **ChrIV_A_nidulans_FGSC_A4:2248022-2248072** | **AN10988** |
| **ChrII_A_nidulans_FGSC_A4:926349-926399** | **AN8152** |
| **ChrIV_A_nidulans_FGSC_A4:2798626-2798676** | **AN7828** |
| **ChrVIII_A_nidulans_FGSC_A4:4748470-4748520** | **AN0050** |
| **ChrVI_A_nidulans_FGSC_A4:375114-375164** | **AN9122** |
| **ChrVI_A_nidulans_FGSC_A4:2147872-2147922** | **AN2996** |
| **ChrV_A_nidulans_FGSC_A4:2024894-2024944** | **AN5670** |
| **ChrI_A_nidulans_FGSC_A4:2904212-2904262** | **AN6702** |
| **ChrIII_A_nidulans_FGSC_A4:119992-120042** | **AN5061** |
| **ChrIII_A_nidulans_FGSC_A4:1843250-1843300** | **AN4502** |
| **ChrII_A_nidulans_FGSC_A4:3145650-3145700** | **AN3761** |
| **ChrII_A_nidulans_FGSC_A4:3534871-3534921** | **AN3633** |
| **ChrVII_A_nidulans_FGSC_A4:337088-337138** | **AN8949** |
| **ChrVII_A_nidulans_FGSC_A4:3971199-3971249** | **AN11340** |
| **ChrVI_A_nidulans_FGSC_A4:2431767-2431817** | **AN11689** |
| **ChrI_A_nidulans_FGSC_A4:1961288-1961338** | **AN10743** |
| **ChrVIII_A_nidulans_FGSC_A4:778819-778869** | **AN1344** |
| **ChrII_A_nidulans_FGSC_A4:1515912-1515962** | **AN4282** |
| **ChrII_A_nidulans_FGSC_A4:3282709-3282759** | **AN3720** |
| **ChrI_A_nidulans_FGSC_A4:250217-250267** | **AN6417** |
| **ChrI_A_nidulans_FGSC_A4:1168067-1168117** | **AN6127** |
| **ChrVIII_A_nidulans_FGSC_A4:1196307-1196357** | **AN1207** |
| **ChrVIII_A_nidulans_FGSC_A4:1692886-1692936** | **AN11290** |
| **ChrVIII_A_nidulans_FGSC_A4:2090968-2091018** | **AN0914** |
| **ChrVII_A_nidulans_FGSC_A4:3934737-3934787** | **AN2430** |
| **ChrVI_A_nidulans_FGSC_A4:88531-88581** | **AN9212** |
| **ChrVI_A_nidulans_FGSC_A4:88531-88581** | **AN9213** |
| **ChrV_A_nidulans_FGSC_A4:2483743-2483793** | **AN5536** |
| **ChrVIII_A_nidulans_FGSC_A4:1959331-1959381** | **AN0954** |
| **ChrI_A_nidulans_FGSC_A4:204490-204540** | **AN10816** |
| **ChrVII_A_nidulans_FGSC_A4:49137-49187** | **AN9045** |
| **ChrIV_A_nidulans_FGSC_A4:797468-797518** | **AN7169** |
| **ChrIV_A_nidulans_FGSC_A4:797468-797518** | **AN10902** |
| **ChrIII_A_nidulans_FGSC_A4:589397-589447** | **AN4908** |
| **ChrIII_A_nidulans_FGSC_A4:1015394-1015444** | **AN4781** |
| **ChrVIII_A_nidulans_FGSC_A4:395885-395935** | **AN9355** |
| **ChrVIII_A_nidulans_FGSC_A4:1114101-1114151** | **AN10173** |
| **ChrVIII_A_nidulans_FGSC_A4:2648696-2648746** | **AN0727** |
| **ChrVIII_A_nidulans_FGSC_A4:4307758-4307808** | **AN0201** |
| **ChrVI_A_nidulans_FGSC_A4:1622036-1622086** | **AN3159** |
| **ChrV_A_nidulans_FGSC_A4:1240439-1240489** | **AN5132** |
| **ChrI_A_nidulans_FGSC_A4:3256263-3256313** | **AN11985** |
| **ChrVI_A_nidulans_FGSC_A4:1923582-1923632** | **AN3076** |
| **ChrIII_A_nidulans_FGSC_A4:3135396-3135446** | **AN8680** |
| **ChrI_A_nidulans_FGSC_A4:3360424-3360474** | **AN6846** |
| **ChrIII_A_nidulans_FGSC_A4:2455885-2455935** | **AN4303** |
| **ChrVIII_A_nidulans_FGSC_A4:826979-827029** | **AN1324** |
| **ChrVI_A_nidulans_FGSC_A4:1722822-1722872** | **AN3134** |
| **ChrVIII_A_nidulans_FGSC_A4:1680391-1680441** | **AN1049** |
| **ChrVII_A_nidulans_FGSC_A4:1376853-1376903** | **AN1620** |
| **ChrIV_A_nidulans_FGSC_A4:278397-278447** | **AN7334** |
| **ChrI_A_nidulans_FGSC_A4:3007697-3007747** | **AN6732** |
| **ChrIII_A_nidulans_FGSC_A4:725430-725480** | **AN4869** |
| **ChrII_A_nidulans_FGSC_A4:711167-711217** | **AN8088** |
| **ChrII_A_nidulans_FGSC_A4:2054791-2054841** | **AN4113** |
| **ChrIV_A_nidulans_FGSC_A4:1652396-1652446** | **AN7474** |
| **ChrI_A_nidulans_FGSC_A4:146513-146563** | **AN6447** |
| **ChrI_A_nidulans_FGSC_A4:286184-286234** | **AN6405** |
| **ChrI_A_nidulans_FGSC_A4:286184-286234** | **AN6406** |
| **ChrVIII_A_nidulans_FGSC_A4:1629149-1629199** | **AN10159** |
| **ChrVIII_A_nidulans_FGSC_A4:4037169-4037219** | **AN10051** |
| **ChrVI_A_nidulans_FGSC_A4:242147-242197** | **AN9161** |
| **ChrVI_A_nidulans_FGSC_A4:242147-242197** | **AN11187** |
| **ChrVI_A_nidulans_FGSC_A4:403539-403589** | **AN11165** |
| **ChrVIII_A_nidulans_FGSC_A4:3955933-3955983** | **AN10064** |
| **ChrVI_A_nidulans_FGSC_A4:1401945-1401995** | **AN3228** |
| **ChrVII_A_nidulans_FGSC_A4:2946093-2946143** | **AN10263** |
| **ChrVII_A_nidulans_FGSC_A4:756889-756939** | **AN1430** |
| **ChrVII_A_nidulans_FGSC_A4:756889-756939** | **AN1429** |
| **ChrII_A_nidulans_FGSC_A4:1405003-1405053** | **AN12208** |
| **ChrII_A_nidulans_FGSC_A4:1405003-1405053** | **AN8291** |
| **ChrIV_A_nidulans_FGSC_A4:1163661-1163711** | **AN7059** |
| **ChrIV_A_nidulans_FGSC_A4:1512477-1512527** | **AN7426** |
| **ChrIV_A_nidulans_FGSC_A4:1512477-1512527** | **AN10971** |
| **ChrI_A_nidulans_FGSC_A4:3301836-3301886** | **AN6827** |
| **ChrI_A_nidulans_FGSC_A4:1156862-1156912** | **AN12442** |
| **ChrIV_A_nidulans_FGSC_A4:646043-646093** | **AN7218** |
| **ChrI_A_nidulans_FGSC_A4:1438425-1438475** | **AN6040** |
| **ChrVII_A_nidulans_FGSC_A4:1508108-1508158** | **AN1654** |
| **ChrVI_A_nidulans_FGSC_A4:1408610-1408660** | **AN3225** |
| **ChrV_A_nidulans_FGSC_A4:738198-738248** | **AN8514** |
| **ChrII_A_nidulans_FGSC_A4:2589162-2589212** | **AN12167** |
| **ChrII_A_nidulans_FGSC_A4:2589162-2589212** | **AN3939** |
| **ChrII_A_nidulans_FGSC_A4:3413265-3413315** | **AN10458** |
| **ChrVII_A_nidulans_FGSC_A4:1881094-1881144** | **AN1786** |
| **ChrV_A_nidulans_FGSC_A4:395414-395464** | **AN8412** |
| **ChrI_A_nidulans_FGSC_A4:206204-206254** | **AN6431** |
| **ChrI_A_nidulans_FGSC_A4:206204-206254** | **AN6432** |
| **ChrVIII_A_nidulans_FGSC_A4:726449-726499** | **AN1370** |
| **ChrI_A_nidulans_FGSC_A4:843009-843059** | **AN6227** |
| **ChrI_A_nidulans_FGSC_A4:1476288-1476338** | **AN10768** |
| **ChrI_A_nidulans_FGSC_A4:1977893-1977943** | **AN5864** |
| **ChrI_A_nidulans_FGSC_A4:2491956-2492006** | **AN6572** |
| **ChrVII_A_nidulans_FGSC_A4:2714103-2714153** | **AN2057** |
| **ChrVI_A_nidulans_FGSC_A4:1846242-1846292** | **AN3098** |
| **ChrV_A_nidulans_FGSC_A4:2519783-2519833** | **AN5524** |
| **ChrVII_A_nidulans_FGSC_A4:3807141-3807191** | **AN12115** |
| **ChrV_A_nidulans_FGSC_A4:2459610-2459660** | **AN5544** |
| **ChrVIII_A_nidulans_FGSC_A4:4048414-4048464** | **AN0281** |
| **ChrI_A_nidulans_FGSC_A4:2772093-2772143** | **AN10838** |
| **ChrVIII_A_nidulans_FGSC_A4:3541273-3541323** | **AN0442** |
| **ChrIII_A_nidulans_FGSC_A4:1202975-1203025** | **AN4716** |
| **ChrVIII_A_nidulans_FGSC_A4:2893515-2893565** | **AN0649** |
| **ChrVII_A_nidulans_FGSC_A4:1060222-1060272** | **AN1522** |
| **ChrV_A_nidulans_FGSC_A4:2865263-2865313** | **AN5422** |
| **ChrV_A_nidulans_FGSC_A4:2865263-2865313** | **AN5421** |
| **ChrV_A_nidulans_FGSC_A4:2216301-2216351** | **AN5613** |
| **ChrIII_A_nidulans_FGSC_A4:2371377-2371427** | **AN4329** |
| **ChrVII_A_nidulans_FGSC_A4:1333717-1333767** | **AN11880** |
| **ChrII_A_nidulans_FGSC_A4:1889640-1889690** | **AN4169** |
| **ChrII_A_nidulans_FGSC_A4:2210625-2210675** | **AN4058** |
| **ChrVI_A_nidulans_FGSC_A4:892993-893043** | **AN3382** |
| **ChrVI_A_nidulans_FGSC_A4:2617801-2617851** | **AN2846** |
| **ChrVI_A_nidulans_FGSC_A4:2805427-2805477** | **AN2789** |
| **ChrII_A_nidulans_FGSC_A4:1597649-1597699** | **AN11432** |
| **ChrII_A_nidulans_FGSC_A4:1597649-1597699** | **AN4258** |
| **ChrI_A_nidulans_FGSC_A4:874257-874307** | **AN11244** |
| **ChrI_A_nidulans_FGSC_A4:2551395-2551445** | **AN6588** |
| **ChrVII_A_nidulans_FGSC_A4:2855339-2855389** | **AN2099** |
| **ChrIII_A_nidulans_FGSC_A4:2315462-2315512** | **AN10548** |
| **ChrV_A_nidulans_FGSC_A4:2419588-2419638** | **AN5557** |
| **ChrIII_A_nidulans_FGSC_A4:401004-401054** | **AN4974** |
| **ChrVI_A_nidulans_FGSC_A4:1683406-1683456** | **AN3144** |
| **ChrV_A_nidulans_FGSC_A4:1965779-1965829** | **AN5693** |
| **ChrV_A_nidulans_FGSC_A4:1965779-1965829** | **AN5692** |
| **ChrII_A_nidulans_FGSC_A4:238623-238673** | **AN7921** |
| **ChrII_A_nidulans_FGSC_A4:2479227-2479277** | **AN12039** |
| **ChrVII_A_nidulans_FGSC_A4:2473340-2473390** | **AN12319** |
| **ChrVII_A_nidulans_FGSC_A4:2473340-2473390** | **AN1988** |
| **ChrV_A_nidulans_FGSC_A4:2848344-2848394** | **AN5427** |
| **ChrVIII_A_nidulans_FGSC_A4:1883718-1883768** | **AN0984** |
| **ChrII_A_nidulans_FGSC_A4:2863279-2863329** | **AN3855** |
| **ChrIV_A_nidulans_FGSC_A4:1642315-1642365** | **AN7471** |
| **ChrIV_A_nidulans_FGSC_A4:1642315-1642365** | **AN7472** |
| **ChrII_A_nidulans_FGSC_A4:577530-577580** | **AN8039** |
| **ChrII_A_nidulans_FGSC_A4:577530-577580** | **AN8040** |
| **ChrI_A_nidulans_FGSC_A4:1760404-1760454** | **AN12161** |
| **ChrVII_A_nidulans_FGSC_A4:3598475-3598525** | **AN2336** |
| **ChrVII_A_nidulans_FGSC_A4:4270732-4270782** | **AN12472** |
| **ChrVII_A_nidulans_FGSC_A4:4270732-4270782** | **AN2540** |
| **ChrVI_A_nidulans_FGSC_A4:1495646-1495696** | **AN3203** |
| **ChrII_A_nidulans_FGSC_A4:3418989-3419039** | **AN3672** |
| **ChrI_A_nidulans_FGSC_A4:1918077-1918127** | **AN5885** |
| **ChrVII_A_nidulans_FGSC_A4:4362251-4362301** | **AN2559** |
| **ChrVI_A_nidulans_FGSC_A4:1219570-1219620** | **AN10390** |
| **ChrVI_A_nidulans_FGSC_A4:1838071-1838121** | **AN3100** |
| **ChrIV_A_nidulans_FGSC_A4:337569-337619** | **AN10927** |
| **ChrIV_A_nidulans_FGSC_A4:835155-835205** | **AN7157** |
| **ChrIV_A_nidulans_FGSC_A4:835155-835205** | **AN7156** |
| **ChrI_A_nidulans_FGSC_A4:3474300-3474350** | **AN6886** |
| **ChrII_A_nidulans_FGSC_A4:13910-13960** | **AN7852** |
| **ChrI_A_nidulans_FGSC_A4:516781-516831** | **AN6328** |
| **ChrI_A_nidulans_FGSC_A4:516781-516831** | **AN6329** |
| **ChrI_A_nidulans_FGSC_A4:3521119-3521169** | **AN6901** |
| **ChrI_A_nidulans_FGSC_A4:2867675-2867725** | **AN6694** |
| **ChrI_A_nidulans_FGSC_A4:2867675-2867725** | **AN6693** |
| **ChrIV_A_nidulans_FGSC_A4:783244-783294** | **AN7172** |
| **ChrI_A_nidulans_FGSC_A4:1538336-1538386** | **AN12262** |
| **ChrI_A_nidulans_FGSC_A4:2722694-2722744** | **AN6646** |
| **ChrIV_A_nidulans_FGSC_A4:1861082-1861132** | **AN7532** |
| **ChrVII_A_nidulans_FGSC_A4:1113786-1113836** | **AN11306** |
| **ChrVII_A_nidulans_FGSC_A4:3853325-3853375** | **AN2415** |
| **ChrVIII_A_nidulans_FGSC_A4:3401282-3401332** | **AN10081** |
| **ChrII_A_nidulans_FGSC_A4:2242313-2242363** | **AN4048** |
| **ChrVII_A_nidulans_FGSC_A4:495481-495531** | **AN12240** |
| **ChrVI_A_nidulans_FGSC_A4:64962-65012** | **AN11191** |
| **ChrVII_A_nidulans_FGSC_A4:2107368-2107418** | **AN1869** |
| **ChrVII_A_nidulans_FGSC_A4:2107368-2107418** | **AN1870** |
| **ChrVII_A_nidulans_FGSC_A4:62642-62692** | **AN11161** |
| **ChrVII_A_nidulans_FGSC_A4:3165756-3165806** | **AN2195** |
| **ChrVII_A_nidulans_FGSC_A4:3165756-3165806** | **AN2194** |
| **ChrIII_A_nidulans_FGSC_A4:1930151-1930201** | **AN10561** |
| **ChrIII_A_nidulans_FGSC_A4:1930151-1930201** | **AN4475** |
| **ChrVI_A_nidulans_FGSC_A4:163333-163383** | **AN9186** |
| **ChrVI_A_nidulans_FGSC_A4:316518-316568** | **AN11168** |
| **ChrI_A_nidulans_FGSC_A4:850344-850394** | **AN6224** |
| **ChrI_A_nidulans_FGSC_A4:850344-850394** | **AN10792** |
| **ChrIII_A_nidulans_FGSC_A4:2444573-2444623** | **AN4309** |
| **ChrVI_A_nidulans_FGSC_A4:393363-393413** | **AN9115** |
| **ChrVI_A_nidulans_FGSC_A4:2332242-2332292** | **AN2939** |
| **ChrIII_A_nidulans_FGSC_A4:426835-426885** | **AN4967** |
| **ChrVIII_A_nidulans_FGSC_A4:3698492-3698542** | **AN0392** |
| **ChrVII_A_nidulans_FGSC_A4:719081-719131** | **AN1416** |
| **ChrI_A_nidulans_FGSC_A4:3316015-3316065** | **AN11988** |
| **ChrIII_A_nidulans_FGSC_A4:1687824-1687874** | **AN4550** |
| **ChrVI_A_nidulans_FGSC_A4:3135501-3135551** | **AN2690** |
| **ChrVI_A_nidulans_FGSC_A4:3135501-3135551** | **AN10333** |
| **ChrVIII_A_nidulans_FGSC_A4:462182-462232** | **AN9380** |
| **ChrV_A_nidulans_FGSC_A4:1454499-1454549** | **AN5196** |
| **ChrII_A_nidulans_FGSC_A4:2073688-2073738** | **AN4105** |
| **ChrII_A_nidulans_FGSC_A4:735229-735279** | **AN8095** |
