# Supplementary material for: A Robust Analytical Pipeline for Genome-Wide Identification of the Genes Regulated by a Transcription Factor: Combinatorial Analysis Performed Using gSELEX-Seq and RNA-Seq
Source: PLoS One. 2016 Jul 13;11(7):e0159011. doi: 10.1371/journal.pone.0159011 (PMC4943734; doi:10.1371/journal.pone.0159011)
Supplement: S2 Table — (DOCX) [file pone.0159011.s004.docx]

**S2 Table. Correlation matrix of the expression level of *A. nidulans* genes detected using RNA-Seq.**

|  | **Δ50**  **+ induction** | **Δ50**  **- induction** | **BPU7**  **+ induction** | **BPU7**  **- induction** |
| --- | --- | --- | --- | --- |
| **Δ50**  **+ induction** | **1.0** | **0.9920701** | **0.9752139** | **0.9861445** |
| **Δ50**  **- induction** | **0.9920701** | **1.0** | **0.9701384** | **0.987315** |
| **BPU7**  **+ induction** | **0.9752139** | **0.9701384** | **1.0** | **0.9752372** |
| **BPU7**  **- induction** | **0.9861445** | **0.987315** | **0.9752372** | **1.0** |
