# Supplementary material for: A Robust Analytical Pipeline for Genome-Wide Identification of the Genes Regulated by a Transcription Factor: Combinatorial Analysis Performed Using gSELEX-Seq and RNA-Seq
Source: PLoS One. 2016 Jul 13;11(7):e0159011. doi: 10.1371/journal.pone.0159011 (PMC4943734; doi:10.1371/journal.pone.0159011)
Supplement: S3 Table — (DOCX) [file pone.0159011.s005.docx]

**S3 Table. DEGs detected in RNA-Seq using RNA from *A. nidulans* BPU7 and Δ50, with or without induction by isomaltose**

| **BPU7 + induction/Δ50 + induction**   \| **ID** \| **Diff *p*-value** \| **Gene** \| **Group in gSELEX** \| \| \| --- \| --- \| --- \| --- \| --- \| \| **AN0941** \| **0** \| **agdE** \| **1** \| \| \| **AN8928** \| **0** \| **atrA** \| **2** \| \| \| **AN8953** \| **0** \| **agdB** \| **1** \| \| \| **AN6473** \| **2.54E-11** \|  \| **-** \| \| \| **AN1797** \| **4.83E-11** \|  \| **5** \| \| \| **AN1866** \| **8.75E-11** \|  \| **-** \| \| \| **AN3402** \| **1.29E-09** \| **amyB** \| **1** \| \| \| **AN4339** \| **2.34E-09** \|  \| **-** \| \| \| **AN6669** \| **4.10E-09** \| **mstC** \| **-** \| \| \| **AN6103** \| **2.30E-08** \|  \| **2** \| \| \| **AN2016** \| **4.52E-08** \| **amyR** \| **-** \| \| \| **AN3882** \| **1.34E-07** \|  \| **-** \| \| \| **AN6274** \| **2.41E-06** \|  \| **-** \| \| \| **AN11864** \| **3.82E-06** \|  \| **-** \| \| \| **AN10420** \| **6.51E-06** \| **agdF** \| **1** \| \| \| **AN3388** \| **7.27E-06** \| **amyF** \| **2** \| \| \| **AN6236** \| **1.48E-05** \| **sidD** \| **5** \| \| \| **AN8637** \| **2.12E-05** \| **catA** \| **-** \| \| \| **AN9183** \| **2.39E-05** \| **bglR** \| **3** \| \| \| **AN2018** \| **2.72E-05** \| **amyA** \| **2** \| \| \| **AN7762** \| **8.03E-05** \|  \| **-** \| \| \| **AN5463** \| **9.22E-05** \|  \| **1** \| \| \| **AN3117** \| **1.34E-04** \|  \| **-** \| \| \| **AN2017** \| **1.54E-04** \| **agdA** \| **3** \| \| \| **AN10081** \| **1.57E-04** \| **xanA** \| **7** \| \| \| **AN9340** \| **1.71E-04** \| **treA** \| **1** \| \| \| **AN8670** \| **1.89E-04** \|  \| **-** \| \| \| **AN3952** \| **1.89E-04** \|  \| **-** \| \| \| **AN3515** \| **1.97E-04** \|  \| **1** \| \| \| **AN4102** \| **2.21E-04** \| **bglA** \| **-** \| \| \| **AN0473** \| **2.49E-04** \|  \| **1** \| \| \| **AN8915** \| **2.67E-04** \|  \| **-** \| \| \| **AN5429** \| **2.92E-04** \|  \| **-** \| \| \| **AN6412** \| **3.06E-04** \| **xtrA** \| **-** \| \| \| **AN2530** \| **4.00E-04** \| **hsp30** \| **-** \| \| \| **AN11778** \| **6.91E-04** \|  \| **1** \| \| \| **AN3781** \| **7.17E-04** \|  \| **-** \| \| \| **AN8956** \| **7.56E-04** \|  \| **-** \| \| \| **AN9320** \| **9.89E-04** \|  \| **-** \| \| \| **AN5823** \| **0.00119** \| **sidA** \| **-** \| \| \| **AN11985** \| **0.00124** \|  \| **7** \| \| \| **AN1864** \| **0.00175** \|  \| **-** \| \| \| **AN8414** \| **0.00177** \| **apdR** \| **2** \| \| \| **AN7357** \| **0.00232** \|  \| **3** \| \| \| **AN3566** \| **0.00243** \| **mns1C** \| **-** \| \| \| **AN7662** \| **0.00302** \| **freA** \| **2** \| \| \| **AN1865** \| **0.0031** \|  \| **-** \| \| \| **AN6237** \| **0.00337** \|  \| **-** \| \| \| **AN0732** \| **0.00361** \|  \| **3** \| \| \| **AN5781** \| **0.00364** \|  \| **-** \| \| \| **AN4313** \| **0.00364** \|  \| **-** \| \| \| **AN3679** \| **0.00364** \|  \| **-** \| \| \| **AN7532** \| **0.00457** \|  \| **7** \| \| \| **AN7011** \| **0.00549** \|  \| **-** \| \| \| **AN8638** \| **0.00549** \| **cetJ** \| **-** \| \| \| **AN8365** \| **0.00573** \|  \| **-** \| \| \| **AN0609** \| **0.00648** \| **sidI** \| **-** \| \| \| **AN2669** \| **0.00894** \|  \| **-** \| \| \| **AN7295** \| **0.00894** \|  \| **-** \| \| \| **AN0693** \| **0.00916** \|  \| **-** \| \| \| **AN3592** \| **0.00982** \| **clxA** \| **-** \| \| \| **AN1918** \| **0.0105** \| **acuF** \| **2** \| \| \| **AN5303** \| **0.0105** \|  \| **-** \| \| \| **AN0942** \| **0.0107** \| **ladA** \| **-** \| \| \| **AN3206** \| **0.0111** \|  \| **-** \| \| \| **AN9339** \| **0.0127** \| **catB** \| **-** \| \| \| **AN5330** \| **0.0139** \|  \| **-** \| \| \| **AN5378** \| **0.0166** \|  \| **3** \| \| \| **AN8279** \| **0.0181** \|  \| **-** \| \| \| **AN2923** \| **0.0182** \|  \| **-** \| \| \| **AN2386** \| **0.0186** \|  \| **-** \| \| \| **AN8777** \| **0.0193** \| **amdS** \| **-** \| \| \| **AN0310** \| **0.0202** \|  \| **-** \| \| \| **AN3196** \| **0.0211** \| **urhA** \| **3** \| \| \| **AN10626** \| **0.0217** \|  \| **-** \| \| \| **AN3996** \| **0.0226** \|  \| **7** \| \| \| **AN4920** \| **0.0271** \| **pmcB** \| **-** \| \| \| **AN1715** \| **0.0279** \|  \| **-** \| \| \| **AN6118** \| **0.0287** \| **agtA** \| **2** \| \| \| **AN11176** \| **0.0287** \|  \| **-** \| \| \| **AN4586** \| **0.0291** \|  \| **1** \| \| \| **AN0886** \| **0.0305** \| **lamB** \| **-** \| \| \| **AN10177** \| **0.0329** \|  \| **3** \| \| \| **AN7594** \| **0.034** \|  \| **1** \| \| \| **AN8903** \| **0.034** \|  \| **-** \| \| \| **AN0248** \| **0.034** \| **pdiB** \| **-** \| \| \| **AN2585** \| **0.034** \|  \| **-** \| \| \| **AN6518** \| **0.0342** \|  \| **-** \| \| \| **AN8368** \| **0.0345** \|  \| **-** \| \| \| **AN6095** \| **0.0347** \| **jenA** \| **2** \| \| \| **AN6239** \| **0.0366** \|  \| **6** \| \| \| **AN7528** \| **0.0371** \|  \| **-** \| \| \| **AN0052** \| **0.0371** \|  \| **2** \| \| \| **AN6113** \| **0.0373** \|  \| **2** \| **2** \| \| **AN8390** \| **0.0378** \|  \| **-** \| \| \| **AN0656** \| **0.0386** \|  \| **-** \| \| \| **AN2922** \| **0.0387** \|  \| **-** \| \| \| **AN3530** \| **0.041** \|  \| **-** \| \| \| **AN1659** \| **0.0426** \|  \| **-** \| \| \| **AN3938** \| **0.043** \|  \| **3** \| \| \| **AN12122** \| **0.0431** \|  \| **3** \| \| \| **AN3555** \| **0.0444** \|  \| **-** \| \| \| **AN8781** \| **0.0444** \|  \| **-** \| \| \| **AN1460** \| **0.0448** \|  \| **-** \| \| \| **AN7619** \| **0.0459** \| **calA** \| **-** \| \| \| **AN0867** \| **0.0491** \|  \| **4** \| \| |
| --- | --- | --- | --- | --- | --- | --- | --- | --- | --- | --- | --- | --- | --- | --- | --- | --- | --- | --- | --- | --- | --- | --- | --- | --- | --- | --- | --- | --- | --- | --- | --- | --- | --- | --- | --- | --- | --- | --- | --- | --- | --- | --- | --- | --- | --- | --- | --- | --- | --- | --- | --- | --- | --- | --- | --- | --- | --- | --- | --- | --- | --- | --- | --- | --- | --- | --- | --- | --- | --- | --- | --- | --- | --- | --- | --- | --- | --- | --- | --- | --- | --- | --- | --- | --- | --- | --- | --- | --- | --- | --- | --- | --- | --- | --- | --- | --- | --- | --- | --- | --- | --- | --- | --- | --- | --- | --- | --- | --- | --- | --- | --- | --- | --- | --- | --- | --- | --- | --- | --- | --- | --- | --- | --- | --- | --- | --- | --- | --- | --- | --- | --- | --- | --- | --- | --- | --- | --- | --- | --- | --- | --- | --- | --- | --- | --- | --- | --- | --- | --- | --- | --- | --- | --- | --- | --- | --- | --- | --- | --- | --- | --- | --- | --- | --- | --- | --- | --- | --- | --- | --- | --- | --- | --- | --- | --- | --- | --- | --- | --- | --- | --- | --- | --- | --- | --- | --- | --- | --- | --- | --- | --- | --- | --- | --- | --- | --- | --- | --- | --- | --- | --- | --- | --- | --- | --- | --- | --- | --- | --- | --- | --- | --- | --- | --- | --- | --- | --- | --- | --- | --- | --- | --- | --- | --- | --- | --- | --- | --- | --- | --- | --- | --- | --- | --- | --- | --- | --- | --- | --- | --- | --- | --- | --- | --- | --- | --- | --- | --- | --- | --- | --- | --- | --- | --- | --- | --- | --- | --- | --- | --- | --- | --- | --- | --- | --- | --- | --- | --- | --- | --- | --- | --- | --- | --- | --- | --- | --- | --- | --- | --- | --- | --- | --- | --- | --- | --- | --- | --- | --- | --- | --- | --- | --- | --- | --- | --- | --- | --- | --- | --- | --- | --- | --- | --- | --- | --- | --- | --- | --- | --- | --- | --- | --- | --- | --- | --- | --- | --- | --- | --- | --- | --- | --- | --- | --- | --- | --- | --- | --- | --- | --- | --- | --- | --- | --- | --- | --- | --- | --- | --- | --- | --- | --- | --- | --- | --- | --- | --- | --- | --- | --- | --- | --- | --- | --- | --- | --- | --- | --- | --- | --- | --- | --- | --- | --- | --- | --- | --- | --- | --- | --- | --- | --- | --- | --- | --- | --- | --- | --- | --- | --- | --- | --- | --- | --- | --- | --- | --- | --- | --- | --- | --- | --- | --- | --- | --- | --- | --- | --- | --- | --- | --- | --- | --- | --- | --- | --- | --- | --- | --- | --- | --- | --- | --- | --- | --- | --- | --- | --- | --- | --- | --- | --- | --- | --- | --- | --- | --- | --- | --- | --- | --- | --- | --- | --- | --- | --- | --- | --- | --- | --- | --- | --- | --- | --- | --- | --- | --- | --- | --- | --- | --- | --- | --- | --- | --- | --- | --- | --- | --- | --- | --- | --- | --- | --- | --- | --- | --- | --- | --- | --- | --- | --- | --- | --- | --- | --- | --- | --- | --- | --- | --- | --- | --- | --- | --- | --- | --- | --- | --- | --- | --- | --- | --- | --- | --- | --- | --- | --- | --- | --- | --- | --- | --- | --- | --- | --- | --- | --- | --- | --- | --- | --- | --- | --- | --- | --- | --- | --- | --- | --- | --- | --- | --- | --- | --- | --- | --- | --- | --- | --- | --- | --- | --- | --- |
| **BPU7 + induction/BPU7 - induction**   \| **ID** \| **Diff *p*-value** \| **Gene** \| **Group in gSELEX** \| \| \| --- \| --- \| --- \| --- \| --- \| \| **AN8953** \| **0** \| **agdB** \| **1** \| \| \| **AN0941** \| **5.98E-13** \| **agdE** \| **1** \| \| \| **AN3402** \| **1.18E-09** \| **amyB** \| **1** \| \| \| **AN8928** \| **2.27E-09** \| **atrA** \| **2** \| \| \| **AN6669** \| **5.86E-09** \| **mstC** \| **-** \| \| \| **AN5463** \| **6.48E-08** \|  \| **1** \| \| \| **AN1797** \| **8.99E-08** \|  \| **5** \| \| \| **AN6473** \| **1.25E-06** \|  \| **-** \| \| \| **AN10420** \| **1.46E-06** \| **agdF** \| **1** \| \| \| **AN3388** \| **2.15E-06** \| **amyF** \| **2** \| \| \| **AN3208** \| **3.04E-06** \|  \| **-** \| \| \| **AN1866** \| **4.81E-06** \|  \| **-** \| \| \| **AN9340** \| **7.23E-06** \| **treA** \| **1** \| \| \| **AN0473** \| **9.05E-06** \|  \| **1** \| \| \| **AN9183** \| **1.40E-05** \| **bglR** \| **3** \| \| \| **AN0732** \| **1.98E-05** \|  \| **3** \| \| \| **AN3117** \| **4.98E-05** \|  \| **-** \| \| \| **AN8365** \| **5.44E-05** \|  \| **-** \| \| \| **AN6103** \| **8.99E-05** \|  \| **2** \| \| \| **AN6236** \| **2.06E-04** \| **sidD** \| **5** \| \| \| **AN8956** \| **3.10E-04** \|  \| **-** \| \| \| **AN3781** \| **3.14E-04** \|  \| **-** \| \| \| **AN1637** \| **3.39E-04** \| **hxB** \| **-** \| \| \| **AN3515** \| **4.62E-04** \|  \| **1** \| \| \| **AN2016** \| **4.62E-04** \| **amyR** \| **-** \| \| \| **AN6237** \| **7.12E-04** \|  \| **-** \| \| \| **AN3725** \| **9.94E-04** \| **awh11** \| **-** \| \| \| **AN3996** \| **0.0012** \|  \| **7** \| \| \| **AN2064** \| **0.0012** \|  \| **-** \| \| \| **AN5330** \| **0.00123** \|  \| **-** \| \| \| **AN8007** \| **0.0013** \| **abnC** \| **-** \| \| \| **AN3433** \| **0.0014** \|  \| **-** \| \| \| **AN3280** \| **0.0014** \|  \| **-** \| \| \| **AN8455** \| **0.00141** \|  \| **-** \| \| \| **AN3872** \| **0.00174** \|  \| **4** \| \| \| **AN2018** \| **0.00191** \| **amyA** \| **2** \| \| \| **AN0609** \| **0.00252** \| **sidI** \| **-** \| \| \| **AN6404** \| **0.00277** \|  \| **-** \| \| \| **AN2146** \| **0.00288** \|  \| **-** \| \| \| **AN10903** \| **0.00289** \|  \| **6** \| \| \| **AN10081** \| **0.00319** \| **xanA** \| **7** \| \| \| **AN9320** \| **0.00397** \|  \| **-** \| \| \| **AN1865** \| **0.00509** \|  \| **-** \| \| \| **AN5228** \| **0.00528** \|  \| **-** \| \| \| **AN6113** \| **0.00579** \|  \| **2** \| **2** \| \| **AN2017** \| **0.00584** \| **agdA** \| **3** \| \| \| **AN3176** \| **0.00585** \| **spb4** \| **3** \| \| \| **AN2669** \| **0.0063** \|  \| **-** \| \| \| **AN4102** \| **0.00661** \| **bglA** \| **-** \| \| \| **AN5017** \| **0.00699** \|  \| **3** \| \| \| **AN0500** \| **0.00764** \|  \| **-** \| \| \| **AN4920** \| **0.00764** \| **pmcB** \| **-** \| \| \| **AN11778** \| **0.01** \|  \| **1** \| \| \| **AN10641** \| **0.0122** \|  \| **-** \| \| \| **AN2894** \| **0.0134** \|  \| **-** \| \| \| **AN2779** \| **0.0137** \|  \| **-** \| \| \| **AN6412** \| **0.0141** \| **xtrA** \| **-** \| \| \| **AN7018** \| **0.0145** \|  \| **-** \| \| \| **AN4586** \| **0.0145** \|  \| **1** \| \| \| **AN7619** \| **0.0145** \| **calA** \| **-** \| \| \| **AN0973** \| **0.0145** \| **brlA** \| **-** \| \| \| **AN8602** \| **0.0158** \|  \| **-** \| \| \| **AN2099** \| **0.0158** \|  \| **7** \| \| \| **AN5339** \| **0.0164** \|  \| **-** \| \| \| **AN7662** \| **0.0169** \| **freA** \| **2** \| \| \| **AN5402** \| **0.0201** \|  \| **-** \| \| \| **AN5340** \| **0.0222** \|  \| **1** \| \| \| **AN5664** \| **0.0254** \|  \| **-** \| \| \| **AN0709** \| **0.0281** \|  \| **-** \| \| \| **AN1754** \| **0.0301** \|  \| **-** \| \| \| **AN4855** \| **0.0301** \|  \| **2** \| \| \| **AN5056** \| **0.0301** \|  \| **-** \| \| \| **AN5324** \| **0.0315** \| **dlpA** \| **-** \| \| \| **AN1696** \| **0.0315** \|  \| **-** \| \| \| **AN0461** \| **0.0317** \|  \| **-** \| \| \| **AN2746** \| **0.0331** \|  \| **3** \| \| \| **AN5037** \| **0.0331** \|  \| **-** \| \| \| **AN0401** \| **0.0333** \|  \| **3** \| \| \| **AN8539** \| **0.0343** \| **ngn26** \| **-** \| \| \| **AN0964** \| **0.0382** \|  \| **1** \| **5** \| \| **AN11120** \| **0.0436** \|  \| **-** \| \| \| **AN0694** \| **0.0436** \|  \| **-** \| \| |
| **BPU7 - induction/Δ50 - induction**   \| **ID** \| **Diff *p*-value** \| **Gene** \| **Group in gSELEX** \| \| --- \| --- \| --- \| --- \| \| **AN3725** \| **5.19E-08** \| **awh11** \| **-** \| \| **AN2016** \| **2.25E-07** \| **amyR** \| **-** \| \| **AN0941** \| **5.08E-07** \| **agdE** \| **1** \| \| **AN8638** \| **9.39E-07** \| **cetJ** \| **-** \| \| **AN1604** \| **2.54E-06** \| **agnE** \| **-** \| \| **AN11152** \| **2.97E-06** \| **gelD** \| **-** \| \| **AN5885** \| **3.12E-06** \| **agsA** \| **7** \| \| **AN3781** \| **9.52E-06** \|  \| **-** \| \| **AN9285** \| **1.33E-05** \| **ccgA** \| **-** \| \| **AN6274** \| **1.35E-05** \|  \| **-** \| \| **AN2894** \| **1.35E-05** \|  \| **-** \| \| **AN2404** \| **1.35E-05** \|  \| **-** \| \| **AN7992** \| **2.55E-05** \|  \| **-** \| \| **AN3703** \| **3.04E-05** \|  \| **-** \| \| **AN8637** \| **4.14E-05** \| **catA** \| **-** \| \| **AN2922** \| **4.56E-05** \|  \| **-** \| \| **AN5664** \| **5.59E-05** \|  \| **-** \| \| **AN0693** \| **5.61E-05** \|  \| **-** \| \| **AN7683** \| **7.91E-05** \|  \| **-** \| \| **AN7539** \| **8.93E-05** \|  \| **-** \| \| **AN8007** \| **9.09E-05** \| **abnC** \| **-** \| \| **AN0973** \| **1.18E-04** \| **brlA** \| **-** \| \| **AN4646** \| **1.68E-04** \|  \| **-** \| \| **AN8956** \| **3.86E-04** \|  \| **-** \| \| **AN8390** \| **7.98E-04** \|  \| **-** \| \| **AN1920** \| **8.46E-04** \|  \| **-** \| \| **AN9306** \| **8.66E-04** \|  \| **-** \| \| **AN3679** \| **0.00122** \|  \| **-** \| \| **AN4299** \| **0.00151** \|  \| **-** \| \| **AN8639** \| **0.003** \|  \| **-** \| \| **AN3161** \| **0.00304** \|  \| **-** \| \| **AN8661** \| **0.00318** \|  \| **-** \| \| **AN0468** \| **0.00341** \|  \| **-** \| \| **AN6869** \| **0.00382** \|  \| **-** \| \| **AN3627** \| **0.00389** \|  \| **-** \| \| **AN6856** \| **0.00391** \|  \| **-** \| \| **AN10626** \| **0.00398** \|  \| **-** \| \| **AN8602** \| **0.00576** \|  \| **-** \| \| **AN7944** \| **0.00687** \| **ngn3** \| **-** \| \| **AN2084** \| **0.0069** \|  \| **-** \| \| **AN5004** \| **0.00771** \|  \| **-** \| \| **AN8627** \| **0.00856** \|  \| **-** \| \| **AN8591** \| **0.00939** \|  \| **6** \| \| **AN8903** \| **0.0106** \|  \| **-** \| \| **AN3520** \| **0.0106** \|  \| **-** \| \| **AN4642** \| **0.0106** \|  \| **-** \| \| **AN0500** \| **0.0127** \|  \| **-** \| \| **AN2810** \| **0.0143** \|  \| **1** \| \| **AN5324** \| **0.0145** \| **dlpA** \| **-** \| \| **AN2923** \| **0.0147** \|  \| **-** \| \| **AN0045** \| **0.0151** \|  \| **-** \| \| **AN7558** \| **0.0173** \|  \| **-** \| \| **AN5437** \| **0.0182** \|  \| **-** \| \| **AN1715** \| **0.0189** \|  \| **-** \| \| **AN2545** \| **0.019** \| **easA** \| **-** \| \| **AN5480** \| **0.0199** \|  \| **-** \| \| **AN9380** \| **0.02** \|  \| **7** \| \| **AN5665** \| **0.0226** \| **CYP531D2** \| **-** \| \| **AN8890** \| **0.0443** \|  \| **-** \| \| **AN4170** \| **0.0495** \| **creD** \| **-** \| |
| **Δ50 + induction/Δ50 - induction**   \| **ID** \| **Diff *p*-value** \| **Gene** \| **Group in gSELEX** \| \| --- \| --- \| --- \| --- \| \| **AN3210** \| **2.10E-08** \|  \| **3** \| \| **AN3781** \| **6.09E-06** \|  \|  \| \| **AN3433** \| **1.05E-04** \|  \|  \| \| **AN0395** \| **1.05E-04** \|  \|  \| \| **AN10767** \| **6.23E-04** \| **fcyB** \|  \| \| **AN6118** \| **7.77E-04** \| **agtA** \| **2** \| \| **AN0732** \| **8.90E-04** \|  \| **3** \| \| **AN5917** \| **0.00328** \|  \|  \| \| **AN8903** \| **0.00908** \|  \|  \| \| **AN8966** \| **0.0116** \|  \|  \| \| **AN6642** \| **0.0227** \| **enaA** \| **3** \| \| **AN3555** \| **0.0452** \|  \|  \| |
