# Supplementary material for: A Robust Analytical Pipeline for Genome-Wide Identification of the Genes Regulated by a Transcription Factor: Combinatorial Analysis Performed Using gSELEX-Seq and RNA-Seq
Source: PLoS One. 2016 Jul 13;11(7):e0159011. doi: 10.1371/journal.pone.0159011 (PMC4943734; doi:10.1371/journal.pone.0159011)
Supplement: S4 Table — (DOCX) [file pone.0159011.s006.docx]

**S4 Table. Overview of 41 genes detected as AmyR- and isomaltose induction-dependent DEGs.**

| **ID** | **Group in gSELEX-Seq** | | **CGGN_8_CGG motif in the promoter region (N)** | **Notes** | | **Description in AspGD [23]*** | |
| --- | --- | --- | --- | --- | --- | --- | --- |
|  |  |  |  |  |  |  |  |
| **AN0473** | **1** | | **4** |  | | **AN0473 Has domain(s) with predicted role in transmembrane transport and integral component of membrane localization.** | |
| **AN0732** | **3** | | **0** | **Was also detected as a DEG between Δ50 + induction and - induction.** | | ***amr1* Putative transporter of the major facilitator superfamily (MFS); expression upregulated after exposure to farnesol.** | |
| **AN0941** | **1** | | **2** | **Was also detected as a DEG between BPU7 - induction and Δ50 - induction, though the *p*-value is considerably high.** | | ***agdE* Protein with alpha-glucosidase activity, predicted role in maltose metabolism; transcriptionally induced by isomaltose in an amyR-dependent manner.** | |
| **AN1797** | **5** | | **0** |  | | **AN1797 Ortholog(s) have fructose transmembrane transporter activity, glucose transmembrane transporter activity, mannose transmembrane transporter activity.** | |
| **AN2017** | **3** | | **3** |  | | ***agdA* Putative alpha-glucosidase with a predicted role in maltose metabolism; transcriptionally induced by isomaltose.** | |
| **AN2018** | **2** | | **4** |  | | ***amyA* Putative alpha-amylase with a predicted role in starch metabolism.** | |
| **AN3388** | **2** | | **3** |  | | ***amyF* Putative alpha-amylase with a predicted role in starch metabolism; transcriptionally induced by isomaltose in an amyR-dependent manner .** | |
| **AN3402** | **1** | | **1** |  | | ***amyB* Putative alpha-amylase with a predicted role in starch metabolism; transcriptionally induced by isomaltose in an amyR-dependent manner.** | |
| **AN3515** | **1** | | **4** |  | | **AN3515 Ortholog(s) have alpha-glucoside:proton symporter activity, maltose:proton symporter activity, trehalose transmembrane transporter activity.** | |
| **AN3996** | **7** | | **0** |  | | **AN3996 Has domain(s) with predicted methyltransferase activity and role in metabolic process.** | |
| **AN4586** | **1** | | **0** |  | | **AN4586 Has domain(s) with predicted nucleic acid binding, zinc ion binding activity.** | |
| **AN5463** | **1** | | **1** |  | | **AN5463 Has domain(s) with predicted starch binding activity and viral capsid localization.** | |
| **AN6103** | **2** | | **3** |  | | **AN6103 Has domain(s) with predicted viral capsid localization.** | |
| **AN6113** | **2** | **2** | **1** |  | **AN6113 Has domain(s) with predicted catalytic activity and role in carbohydrate metabolic process.** | |  |
| **AN6236** | **5** | | **1** |  | | ***sidD* Nonribosomal peptide synthetase (NRPS); predicted backbone enzyme of a siderophore secondary metabolism biosynthetic gene cluster member.** | |
| **AN7662** | **2** | | **4** |  | | ***freA* Putative metalloreductase with a predicted role in iron homeostasis; regulated by iron independently of SreA.** | |
| **AN8928** | **2** | | **0** |  | | ***atrA* Putative plasma membrane ATP-binding cassette (ABC) transporter with a predicted role in multidrug resistance; transcript induced by the fungicide imazalil.** | |
| **AN8953** | **1** | | **2** |  | | ***agdB* Putative alpha-glucosidase with a predicted role in maltose metabolism; transcriptionally induced by isomaltose; induced by rapamycin-induced autophagy.** | |
| **AN9183** | **3** | | **1** |  | | ***bglR* Putative beta-glucosidase with a predicted role in polysaccharide degradation.** | |
| **AN9340** | **1** | | **0** |  | | ***treA* Alpha, alpha-trehalase with a role in trehalose hydrolysis; localized to the conidial cell wall; expression upregulated after exposure to farnesol.** | |
| **AN10081** | **7** | | **1** |  | | ***xanA* Alpha-ketoglutarate-dependent xanthine dioxygenase involved in the oxidation of xanthine to uric acid; regulated by uaY and AreA transcription factors; the 5' region of xanA is included in a helitron fragment and is duplicated in the genome.** | |
| **AN10420** | **1** | | **4** |  | | ***agdF* Putative alpha-glucosidase with a predicted role in starch metabolism; transcriptionally induced by isomaltose in an amyR-dependent manner.** | |
| **AN11778** | **1** | | **3** |  | | **AN11778 Putative exoinulinase.** | |
| **AN0609** | **-** | | **2** |  | | **sidI Triacetylfusarinine C (TAFC) biosynthetic enoyl-CoA hydratase; siderophore biosynthetic enzyme.** | |
| **AN1865** | **-** | | **0** |  | | **AN1865 Putative sugar transporter.** | |
| **AN1866** | **-** | | **0** |  | | **AN1866 Ortholog of A. nidulans FGSC A4 : AN7868, A. fumigatus Af293 : Afu2g04540, A. niger CBS 513.88 : An04g07800, A. oryzae RIB40 : AO090003000256 and Neosartorya fischeri NRRL 181 : NFIA_057620, NFIA_081290.** | |
| **AN2016** | **-** | | **1** | **Codes AmyR** | | **amyR Zn(II)2 Cys6 transcriptional activator involved in starch metabolism; responsible for induction of amylolytic genes; localizes to the nucleus in response to maltose, isomaltose, kojibiose and D-glucose.** | |
| **AN2669** | **-** | | **0** |  | | **AN2669 Has domain(s) with predicted role in response to stress and integral component of membrane localization.** | |
| **AN3117** | **-** | | **2** |  | | **AN3117 Ortholog(s) have copper-exporting ATPase activity, role in cadmium ion transport, cellular copper ion homeostasis, copper ion transport, silver ion transport and plasma membrane localization.** | |
| **AN3781** | **-** | | **0** | **Was also detected as a DEG between BPU7 - induction and Δ50 - induction/ Δ50 + induction and - induction.** | | **AN3781 Has domain(s) with predicted inorganic phosphate transmembrane transporter activity, role in phosphate ion transport and membrane localization.** | |
| **AN4102** | **-** | | **4** |  | | **bglA Putative beta-glucosidase; induced by carbon starvation-induced autophagy.** | |
| **AN4920** | **-** | | **0** |  | | **pmcB Putative calcium-transporting mitochondrial ATPase involved in calcium homeostasis.** | |
| **AN5330** | **-** | | **0** |  | | **AN5330 Has domain(s) with predicted N-acetyltransferase activity.** | |
| **AN6237** | **-** | | **0** |  | | **AN6237 Has domain(s) with predicted ATP binding, ATPase activity, ATPase activity, coupled to transmembrane movement of substances, nucleoside-triphosphatase activity, nucleotide binding activity and role in transmembrane transport.** | |
| **AN6412** | **-** | | **0** |  | | **xtrA Putative xylose transporter.** | |
| **AN6473** | **-** | | **0** |  | | **AN6473 Ortholog of A. fumigatus Af293 : Afu1g16430, Neosartorya fischeri NRRL 181 : NFIA_009010, Aspergillus versicolor : Aspve1_0315435 and Aspergillus fumigatus A1163 : AFUB_015770.** | |
| **AN6669** | **-** | | **1** |  | | **mstC High-affinity glucose transporter active in germinating conidia.** | |
| **AN7619** | **-** | | **0** |  | | **calA Secreted thaumatin-like protein; role in early conidial germination; localized to the cell wall of germinating conidia but not to germ tubes; null appears wild-type, shows severe conidial germination defects with cetA calA double mutant.** | |
| **AN8365** | **-** | | **1** |  | | **AN8365 Has domain(s) with predicted role in transmembrane transport and integral component of membrane localization.** | |
| **AN8956** | **-** | | **0** | **Was also detected as a DEG between BPU7 - induction and Δ50 - induction.** | | **AN8956 Has domain(s) with predicted inorganic phosphate transmembrane transporter activity, role in phosphate ion transport and membrane localization.** | |
| **AN9320** | **-** | | **0** |  | | **AN9320 Ortholog of Aspergillus flavus NRRL 3357 : AFL2T_11783, Aspergillus wentii : Aspwe1_0104603, Aspergillus versicolor : Aspve1_0047986 and Aspergillus sydowii : Aspsy1_0051540.** | |

*URL: http://www.aspgd.org
